# Development of an oral regimen of unithiol for the treatment of snakebite envenoming: a phase 1 open-label dose-escalation safety trial and pharmacokinetic analysis in healthy Kenyan adults

**DOI:** 10.1016/j.ebiom.2025.105600

**Published:** 2025-02-27

**Authors:** Michael Abouyannis, Yvonne K. Nyambura, Samson Ngome, Debra Riako, Jennifer Musyoki, Charles Muiruri, Benedict Orindi, Laura Else, Alieu Amara, Laura Dickinson, Rachel H. Clare, Laura-Oana Albulescu, Adam P. Westhorpe, Jeroen Kool, Ifedayo Adetifa, Francis M. Ndungu, Richard FitzGerald, Saye Khoo, David G. Lalloo, Nicholas R. Casewell, Mainga Hamaluba

**Affiliations:** a Centre for Snakebite Research & Interventions, Liverpool School of Tropical Medicine, Liverpool, UK; b KEMRI-Wellcome Research Programme, Kilifi, Kenya; c Department of Molecular and Clinical Pharmacology, University of Liverpool, Liverpool, UK; d Division of BioAnalytical Chemistry, Amsterdam Institute of Molecular and Life Sciences (AIMMS), Vrije Universiteit Amsterdam, De Boelelaan 1085, 1081 HV, Amsterdam, the Netherlands; e Centre for Analytical Sciences Amsterdam (CASA), 1098 XH, Amsterdam, the Netherlands; f Department of Infectious Diseases Epidemiology, London School of Hygiene & Tropical Medicine, London, UK; g NIHR Royal Liverpool and Broadgreen CRF, Liverpool University Hospitals NHS Foundation Trust, Liverpool, UK; h Centre for Tropical Medicine & Global Health, Nuffield Department of Medicine, University of Oxford, Oxford, UK

**Keywords:** Phase 1 clinical trial, Snakebite, Envenoming, Unithiol

## Abstract

**Background:**

Viperidae snakes are responsible for many of the 94,000 deaths caused by snakebite envenoming each year. The most pathological venom component of this globally diverse family of snakes are the zinc-dependent snake venom metalloproteinase (SVMP) enzymes, which can be inhibited by the metal chelator, unithiol. A short-course oral regimen, readily available and rapidly deployed ahead of hospital admission is needed.

**Methods:**

This open-label, phase 1 clinical trial assessed the safety of single ascending oral, multiple ascending oral, and single ascending intravenous doses of unithiol in 64 healthy adult volunteers from Kilifi County, Kenya. The multiple dose stage was informed by an interim safety and pharmacokinetic analysis, and predefined target plasma concentrations. Plasma concentrations of unithiol were measured using high-performance liquid chromatography-mass spectrometry, and safety was described by full adverse event reporting.

**Findings:**

175 individuals were screened, and 64 (median age 30 years, IQR 25–38 years) received the study drug. There were no dose limiting toxicities or serious adverse events. There were 61 solicited adverse events, 17 related unsolicited adverse events, and 53 laboratory adverse events, all of mild or moderate severity. The maximum oral dose of 1500 mg was well tolerated and associated with the following pharmacokinetic parameters: C_max_ 14.7 μg/mL, T_max_ 2.9 h, T_1/2_ 18.4 h, and AUC_0-∞_ 204.5 μg.h/mL.

**Interpretation:**

The phase 2 recommended dose (1500 mg loading dose, followed by 900 mg doses at 6-h and 24-h) has no safety concerns, and has promising pharmacokinetic properties for clinical use. Unithiol is affordable, stable at room temperature, and has the potential to be given orally in remote rural clinics. Its further development for snakebite indication is warranted.

**Funding:**

10.13039/100010269Wellcome Trust, Bloomsbury Set, and 10.13039/100016374Cures Within Reach.

**Research in context**

**Evidence before this study**

We conducted a systematic review to understand the landscape of therapeutic clinical trials for snakebite envenoming.^1^ The overwhelming majority of trials have evaluated animal derived antivenoms, which have many limitations including a narrow spectrum of snake–species activity, prohibitive production costs, high rates of anaphylactoid reactions, and the requirement for intravenous administration. Alternative therapeutics are urgently needed.

A large proportion of the world's medically important snake species cause toxicity through snake venom metalloproteinase (SVMP) activity, a group of zinc dependent enzymes that are vulnerable to inhibition through chelation. Unithiol, a chelating agent that is routinely used to treat mercury and arsenic poisoning, has recently demonstrated promising efficacy to prevent tissue damage and death in animal models of snakebite envenoming.

The optimal dose of unithiol for snakebite envenoming is uncertain, and this clinical trial was undertaken to define the safety and pharmacokinetics of oral dose escalations amongst healthy adult volunteers.

**Added value of this study**

This study demonstrated reassuring safety of increasing oral doses of unithiol in a Kenyan population. A phase 2 recommended multiple oral dosing regimen was developed. The pharmacokinetic profile of this regimen was promising, with resulting plasma concentrations above targets that have been associated with SVMP inhibition in *in vitro* and *in vivo* studies.

**Implications of all the available evidence**

The safety and pharmacokinetic profile of the phase 2 recommended dosing regimen of unithiol was promising and warrants evaluation in future trials to assess clinical efficacy. There is an urgent need to develop affordable, safe, and orally administered treatments for snakebite envenoming.

## Introduction

Snakebite predominantly affects marginalised communities in the rural tropics, causing an estimated 1.8 million envenomings and 94,000 deaths each year.^2^^,^^3^ Antivenoms, made from the sera of large mammals exposed to repeated immunisations of venom from medically important snake species, are the only approved treatments for snakebite envenoming. These products have a narrow spectrum of snake–species activity, are prohibitively expensive, frequently cause anaphylactoid reactions, require intravenous administration, and have poor tissue penetration.^4^, ^5^, ^6^ The antivenom market is fragmented and fragile, products are of varying quality, and stocks are limited to secondary care facilities that are distant to the remote rural locations where snakebite tends to occur,^1^^,^^7^, ^8^, ^9^, ^10^, ^11^ resulting in treatment delays that can contribute to poor patient outcomes.

The snake venom metalloproteinase (SVMP) enzymes are a central pathological component of the venom of many medically important snake species, particularly those of vipers, including those found across Asia (*Daboia* and *Echis* spp.), Africa (*Bitis* and *Echis* spp.), North America (*Crotalus* spp.), and South America (*Bothrops* spp.).^12^ The SVMPs are zinc-dependent enzymes and are susceptible to inactivation by chelating agents.^13^ Systematic *in vitro* and *in vivo* assessment previously identified unithiol (molecular name, DMPS [i.e., 2,3-bis(sulfanyl)propane-1-sulfonic acid]) as the most promising approved metal chelator for snakebite.^14^ Unithiol is safe,^15^ affordable, can be administered intravenously or orally,^16^^,^^17^ and is in routine clinical use for heavy metal poisoning.^18^^,^^19^ Orally administered unithiol prevented *Echis* spp. venom-induced mortality and local haemorrhage in a murine model,^14^ abrogated the dermonecrotic effects of globally diverse snake species, and had an additive effect to antivenom and varespladib.^20^

Unithiol was developed in what is now the geographic region of Ukraine, in 1956.^21^ The only previous reported healthy volunteer pharmacokinetic studies assessed single 300 mg oral doses, without dose escalation.^22^^,^^23^ The oral regimen tends to be administered at a lower dose over a period of weeks–months, to facilitate clearance of heavy metals.^24^ Conversely, people with snakebite are likely to benefit from a higher-dose short-course regimen. Unithiol has scarcely been used in snakebite endemic settings, such as Africa, and safety in these populations needs to be explored. To address these important data gaps, in a programme of work to prioritise translation of orally administered snake venom toxin inhibitors, we have evaluated the safety, tolerability, and pharmacokinetics of escalated oral doses and standard intravenous doses of unithiol, in healthy adult volunteers from a snakebite endemic region in south-east Kenya.

## Methods

### Study design

This open-label, phase 1, dose-escalation clinical trial was conducted in healthy adult volunteers at the Kenya Medical Research Institute-Wellcome Trust Research Programme (KWTRP) clinical trials facility in Kilifi County, Kenya. The primary objective was to determine the safety of oral unithiol dose escalations.^25^ The secondary objective was to define the pharmacokinetic profile of the study drug. In the first stage of the trial, four cohorts of eight participants received single ascending doses (SAD) of oral unithiol, with dose escalation decisions dependent on emerging safety data, predefined holding criteria (Appendix, p 1), and approval from an independent data safety monitoring board (DSMB). The DSMB met and ratified every dose escalation decision, except for the increase from the single oral 300 mg (the currently established dose of unithiol) to the single oral 900 mg cohort. An interim pharmacokinetic analysis was completed, which informed the multiple ascending dose (MAD) second stage. Additionally, two cohorts of eight participants received single intravenous doses of unithiol.

### Ethics

All participants provided written informed consent. The study obtained ethical approval from the Kenya Medical Research Institute Scientific Ethics Review Unit (192/4106) and the Liverpool School of Tropical Medicine Research Ethics Committee (20-032), and regulatory approval from the Pharmacy and Poisons Board of Kenya (136). The trial was conducted in accordance with regulatory requirements, including International Conference on Harmonisation-Good Clinical Practice guidelines.

### Participants

Community engagement was facilitated by the KWTRP Community Liaison Group (CLG).^26^ Once community stakeholders had been engaged, members of Ngerenya village, which is within the Kilifi Health and Demographic Surveillance System (KHDSS)^27^ study area, were approached through barazas (open air public meetings chaired by the local chief), health talks, and the mobilisation of community health volunteers. Potential participants were given ample time to consider participation prior to providing written informed consent. Screening took place at the KWTRP clinical trial facility designated site–Pwani University, Kilifi. All verbal and written information were provided in English, Giriama, and Swahili languages.

Full eligibility criteria are available (Appendix, p 2–3), but in brief, healthy adults aged 18–64-years, weighing 50–120 kg, and residing in the KHDSS study area^27^ were eligible. Women who were pregnant, breastfeeding, or not taking reliable contraception were excluded. People with asthma, HIV, abnormal clinical laboratory findings, or an abnormal electrocardiogram were excluded. Eligibility was reassessed within 24 h of dosing.

### Study procedures

Unithiol (trade name, Dimaval®) was donated by the manufacturer (Heyl Chemisch-pharmazeutische Fabrik GmbH & Co, Germany); the oral formulation was 100 mg capsules, and the solution for injection was 250 mg/5 mL ampoules.

Sixty-four eligible participants were allocated to eight dosing cohorts using stratified permuted block randomisation (the block size was 8), with stratification by age and sex (self-reported), by a blinded data manager (CM) using the blockrand package version 1.5 in R version 4.1.2. Reserve participants replaced any individuals who were withdrawn or excluded from the trial.

The first cohort (C1) received 300 mg single oral doses, an established dose with a reassuring safety profile.^22^^,^^23^^,^^28^ The pre-specified single oral dose escalations were 900 mg (C2), 1200 mg (C3), and 1500 mg (C4) (Table 1). One participant from each of these cohorts (C2–C4) was randomly allocated to be dosed and monitored for safety 1-day prior to the remaining seven participants (sentinel dosing). This was an additional risk mitigation strategy for participants receiving these higher oral doses. The maximum dose was set based on *in vitro* studies which demonstrated that a unithiol concentration of 3.1 μg/mL was sufficient to inhibit SVMP driven substrate hydrolysis by venom from a geographically diverse range of *Echis* spp.,^14^ whilst a second *in vitro* assay demonstrated that unithiol concentrations of 30.7 μg/mL were necessary to inhibit the procoagulant activity of such venoms.^14^ Based on a target plasma concentration of between 3.1 μg/mL and 30.7 μg/mL, and background pharmacokinetic data demonstrating that a 300 mg oral dose was associated with a C_max_ of 5.7 μg/mL,^23^ it was estimated, assuming dose proportional pharmacokinetics, that a 1500 mg oral dose would be sufficient. The selected intravenous doses, of 3 mg/kg and 5 mg/kg, have commonly been used in acute heavy metal poisoning,^29^^,^^30^ and background pharmacokinetic data have demonstrated that a 3 mg/kg dose produces a sufficient C_max_ of approximately 23 μg/mL.^31^

**Table 1:** Overview of the eight dosing cohorts.

Dosing cohort	Number of participants	Dose of unithiol
**Oral single ascending dose stage**		
Cohort 1 (C1)	8	300 mg oral
Cohort 2 (C2)	8	900 mg oral
Cohort 3 (C3)	8	1200 mg oral
Cohort 4 (C4)	8	1500 mg oral
**Oral multiple ascending dose stage**		
Cohort Multiple 1 (CM1)	8	0-h: 1500 mg oral 6-h: 900 mg oral 24-h: 900 mg oral
Cohort Multiple 2 (CM2)	8	0-h: 1500 mg oral 6-h: 1500 mg oral 24-h: 1500 mg oral
**Intravenous single ascending dose stage**		
Cohort Intravenous 1 (CIV1)	8	3 mg/kg intravenous
Cohort Intravenous 2 (CIV2)	8	5 mg/kg intravenous

Participants underwent regular clinical, laboratory and electrocardiogram assessments to monitor for adverse events. Data were entered on paper Case Reports Forms (CRF) that were subsequently transcribed to an electronic REDCap® database. Lab samples were tracked and clinical results reported using the Kilifi Integrated Data Management System (KIDMS). Assessments were undertaken as an inpatient for 24 h after dose administration; as scheduled clinic visits on days 2, 5, and 42; and via a final telephone follow-up at 6 months. Study participants were advised to report any substantial health events to the research clinic throughout the study period. Participants were monitored for serious adverse events and suspected unexpected serious adverse reactions. Adverse events were regarded as having a known pattern of response to the study drug (for the purpose of categorising a causal relationship) if they were listed on the Summary of Product Characteristics (SmPC)^16^^,^^17^ (Appendix, p 4). There was active reporting, at every study visit, for adverse events listed on the SmPC (‘solicited adverse events’) (Appendix, p 4), and passive reporting, throughout the 6-month follow-up period, for all other adverse events (‘unsolicited adverse events’). Adverse events were named according to the Medical Dictionary for Regulatory Activities (MedDRA®) version 26.0, and were graded according to the Division of Acquired Immunodeficiency Syndrome (DAIDS) criteria.^32^ For laboratory adverse events, the DAIDS criteria were modified to reflect local reference ranges.

Frequent serial blood sampling was undertaken for the pharmacokinetic analysis (Appendix p 5). Unithiol has two thiol (sulfhydryl) groups that are susceptible to *ex vivo* oxidation, which may lead to underestimation of plasma concentrations. Therefore, total unithiol plasma concentrations, a combination of the altered and unaltered forms, were measured. This involved pre-treating the plasma samples with a reducing agent (dithiothreitol, DTT fluorochem, UK) to convert the oxidised (altered) form back to free (unaltered) unithiol, prior to quantification.^33^ High-performance liquid chromatography-mass spectrometry (LC-MS) was conducted at the University of Liverpool Bioanalytical Facility, in accordance with Good Clinical Laboratory Practice (ISBN 1-904610-00-5, 2003). In brief, DTT pre-treated plasma was precipitated with acetonitrile (1500 μL) containing an internal standard (hexobarbital; 250 μg/mL). Unithiol (*m*/*z* 186.9 → 80.9) was eluted using a C_18_ column (Atlantis C_18_, 3 μm × 2.1 × 100 mm; Waters, MA, US) and quantification was performed using a SCIEX 4500 triple quadrupole mass spectrometer (SCIEX, Alderley Park, UK) operating in negative ionisation mode. The assay was linear over a calibration range of 0.1–40 μg/mL and recovery of unithiol was >90%.

This trial was registered on the Pan African Clinical Trials Registry (PACTR202103718625048) and the protocol was published.^25^ This trial was reported with reference to the CONsolidated Standards Of Reporting Trials Dose-finding Extension (CONSORT-DEFINE) guidance (Appendix, p 26–36).^34^

### Statistics

A formal sample size calculation was not conducted. Participants were dosed in groups of eight, which is a commonly used design in phase 1 clinical trials. Safety data were summarised using numbers and proportions, together with their associated 95% Wilson confidence intervals. The safety analysis was performed using R version 4.3.0.^35^ The pharmacokinetic non-compartmental analysis was performed using Phoenix 64 WinNonlin version 8.3 software, to calculate the following parameters: maximum concentration (C_max_), time to maximum concentration (T_max_), elimination half-life (t_1/2_), last measurable concentration (C_last_), and the area under the concentration–time curve (AUC). Oral bioavailability was estimated using the following formula: (median oral AUC_0-∞_/median intravenous AUC_0-∞_) x (median intravenous dose/median oral dose) x 100.

### Role of funders

The funders (Wellcome Trust, Bloomsbury Set, and Cures Within Reach) had no role in the study design, data analysis, or the decision to publish the results. All authors had access to the data and were responsible for the decision to submit the manuscript for publication.

## Results

### Enrolment and baseline characteristics

**Fig. 1:**
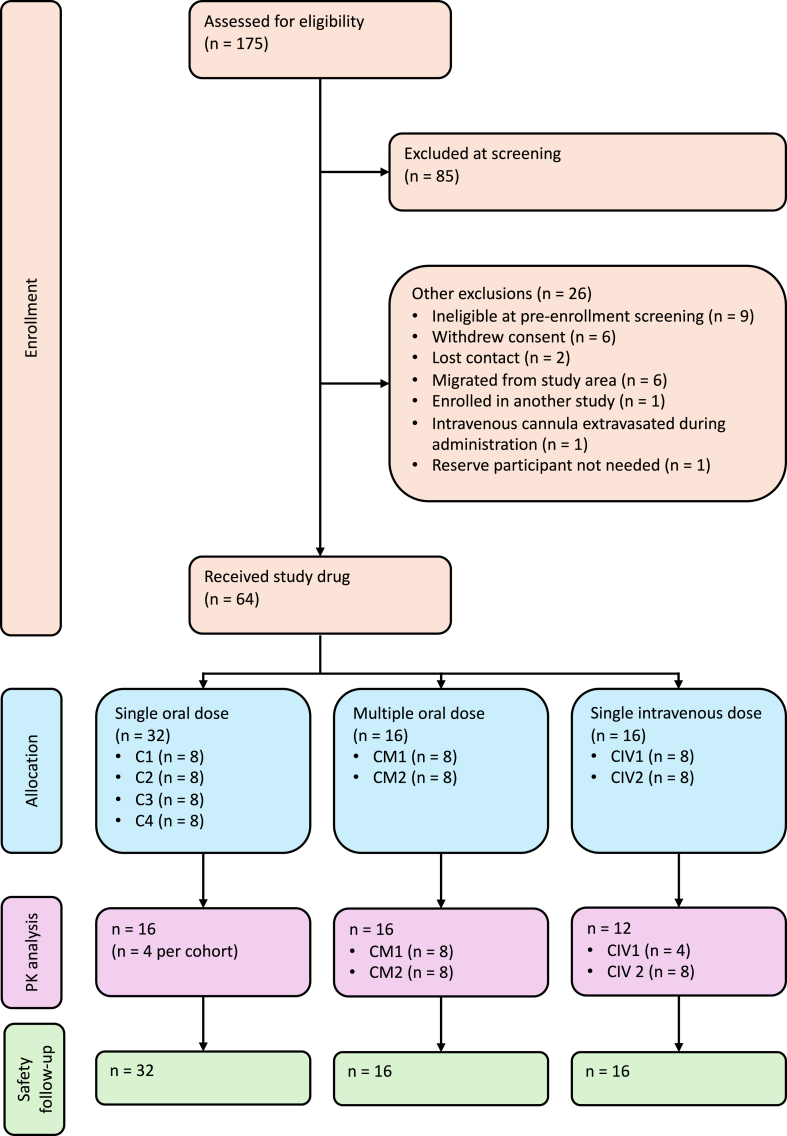
**CONSORT flow diagram of screening, allocation, and follow-up**. PK: pharmacokinetic.

Between March 2022 and February 2023, 175 individuals were screened, and 90 eligible participants were identified (Fig. 1 and Appendix p 6). Study drug was administered to 64 participants (referred to herein as the safety population). Four cohorts of eight participants received single ascending oral doses, two cohorts received multiple oral doses, and two cohorts received single intravenous doses. The median age of the safety population at enrolment was 30 years (IQR 25–38 years) and there were 55 (85.9%) male participants. The baseline characteristics of the safety population has been summarised in Table 2.

**Table 2:** Baseline characteristics of the safety population.

Dose	Number	Male (n, %)	Age, years (median, IQR)	Weight, kg (median, IQR)	Creatinine, μmol/L (median, IQR)
300 mg oral	8	7 (88%)	38 (IQR 28–52)	61 (IQR 58–65)	89 (IQR 87–95)
900 mg oral	8	8 (100%)	32 (IQR 26–36)	59 (IQR 57–65)	104 (IQR 87–106)
1200 mg oral	8	6 (75%)	28 (IQR 25–32)	58 (IQR 56–65)	88 (IQR 82–99)
1500 mg oral	8	6 (75%)	28 (IQR 24–32)	60 (IQR 59–63)	97 (IQR 86–103)
3 mg/kg intravenous	8	7 (88%)	28 (IQR 24–33)	64 (IQR 61–68)	102 (IQR 98–104)
5 mg/kg intravenous	8	7 (88%)	36 (IQR 29–43)	62 (IQR 59–64)	102 (IQR 95–106)
0-h: 1500 mg oral 6-h: 900 mg oral 24-h: 900 mg oral	8	7 (88%)	26 (IQR 22–36)	55 (IQR 53–57)	90 (IQR 85–98)
0-h: 1500 mg oral 6-h: 1500 mg oral 24-h: 1500 mg oral	8	7 (88%)	25 (IQR 24–34)	58 (IQR 54–62)	95 (IQR 95–96)
**All**	**64**	**55 (86%)**	**30 (IQR 25**–**37)**	**60 (IQR 56**–**65)**	**96 (IQR 87**–**103)**

IQR: interquartile range; kg: kilograms.

### Single ascending oral doses

The single ascending dose stage was completed as planned, with four cohorts of eight participants being escalated from a 300 mg oral dose to the 1500 mg intended maximum dose. No deaths, serious adverse events, or discontinuations occurred. There were 39 solicited adverse events, of which 38 were mild and one (abdominal pain) was moderate in severity (Table 3). A full breakdown of solicited, unsolicited and laboratory adverse events with proportions and 95% confidence intervals in available in the Supplementary materials (Appendix, p 7–9). At least one solicited adverse event was experienced by 15 (47%) of 32 participants. The solicited adverse events that most frequently occurred were abdominal pain (8 [21%] of 39 occurrences) and skin reaction (7 [18%] of 39 occurrences).

**Table 3:** Adverse events following administration of unithiol in the safety population.

	Single oral dose				Multiple oral dose		Single intravenous dose	
	C1 (n = 8)	C2 (n = 8)	C3 (n = 8)	C4 (n = 8)	CM1 (n = 8)	CM2 (n = 8)	CIV1 (n = 8)	CIV2 (n = 8)
**Number of SAEs**	0	0	0	0	0	0	0	0
**Number of deaths**	0	0	0	0	0	0	0	0
**No. of solicited AEs**	16	11	8	4	1	1	15	5
**No. of Unsolicited AEs**	2	4	5	2	1	0	3	0
**No. of Laboratory AEs**	7	9	5	2	7	8	5	12
**Solicited AEs**^b^								
Abdominal pain	3 (19%, 7–43)	2 (18%, 5–48)	2 (25%, 7–59)	1 (25%, 5–70)	0 (0%, 0–79)	0 (0%, 0–79)	4 (27%, 11–52)	1 (20%, 4–62)
Dysgeusia	2 (13%, 3–36)	0 (0%, 0–26)	0 (0%, 0–32)	0 (0%, 0–50)	0 (0%, 0–79)	0 (0%, 0–79)	1 (7%, 1–30)	1 (20%, 4–62)
Fever	1 (6%, 1–28)	0 (0%, 0–26)	0 (0%, 0–32)	1 (25%, 5–70)	0 (0%, 0–79)	0 (0%, 0–79)	0 (0%, 0–20)	0 (0%, 0–43)
Injection site pain	–	–	–	–	–	–	2 (13%, 4–38)	0 (0%, 0–43)
Decreased appetite	4 (25%, 10–49)	1 (9%, 2–38)	1 (13%, 2–47)	0 (0%, 0–50)	0 (0%, 0–79)	0 (0%, 0–79)	3 (20%, 7–45)	1 (20%, 4–62)
Nausea	2 (13%, 3–36)	1 (9%, 2–38)	2 (25%, 7–59)	1 (25%, 5–70)	0 (0%, 0–79)	0 (0%, 0–79)	1 (7%, 1–30)	0 (0%, 0–43)
Shivering	1 (6%, 1–28)	1 (9%, 2–38)	0 (0%, 0–32)	0 (0%, 0–50)	0 (0%, 0–79)	0 (0%, 0–79)	0 (0%, 0–20)	0 (0%, 0–43)
Skin reaction	0 (0%, 0–19)	5 (45%, 21–72)	2 (25%, 7–59)	0 (0%, 0–50)	1 (100%, 21–100)	1 (100%, 21–100)	3 (20%, 7–45)	1 (20%, 4–62)
Muscular weakness	3 (19%, 7–43)	1 (9%, 2–38)	1 (13%, 2–47)	1 (25%, 5–70)	0 (0%, 0–79)	0 (0%, 0–79)	1 (7%, 1–30)	1 (20%, 4–62)
Hypotension^a^	–	–	–	–	–	–	0 (0%, 0–20)	0 (0%, 0–43)
SJS	0 (0%, 0–19)	0 (0%, 0–26)	0 (0%, 0–32)	0 (0%, 0–50)	0 (0%, 0–79)	0 (0%, 0–79)	0 (0%, 0–20)	0 (0%, 0–43)

AE: adverse events; SAE: serious adverse events; SJS: Stevens-Johnson's syndrome; dash (–): were not assessed for those particular groups.

a Hypotension occurring within 15-min of intravenous administration of the study drug.

b Percentages are based on number of solicited AEs for each group.

There were 13 unsolicited adverse events with at least a possible causal relationship with the study drug (Table 3), of which 12 were mild and one (heavy menstrual bleeding) was moderate in severity. One participant had an episode of hypoaesthesia, which was described as an isolated loss of sensation in the upper left limb that started 3 days after receiving the study drug. This event was self-limiting and spontaneously resolved after 2 days, with no treatment being necessary. The anatomical distribution of this was not characteristic of a dermatome or mononeuropathy.

There were 23 laboratory adverse events reported for 17 participants (Table 3). These were mild severity events in 18 cases and moderate in 4 (1 neutropaenia, 1 thrombocytopaenia, 1 raised alanine aminotransferase [ALT], and 1 hypernatraemia), none of which required medical intervention.

Full line listings for all adverse events are available in the Supplementary materials (Appendix, p 10–18). The temporal trends of laboratory parameters for participants with transaminitis, leukopaenia or increased blood creatinine have been further detailed in the Supplementary materials, because these side effects have previously been associated with unithiol (Appendix p 19–20).^16^

### Single ascending intravenous doses

One cohort of eight participants received single 3 mg/kg intravenous infusions of unithiol (CIV1), and a second cohort received single 5 mg/kg infusions (CIV2). There were no deaths, serious adverse events, or discontinuations. There were 20 solicited adverse events, 17 of which were mild and 3 of which were moderate in severity (Table 3). The moderately severe events included one (20%) of five abdominal pain events and two (50%) of four skin reaction events. There were three unsolicited adverse events with at least a possible causal relationship with the study drug (Table 3), all of which were classified as mild cases of viral gastroenteritis. There were 17 laboratory adverse events (Table 3), which included two participants with a moderately severe (grade 2) transaminitis (increased ALT). In both these cases, the ALT spontaneously normalised without any specific management.

### Interim analysis

**Fig. 2:**
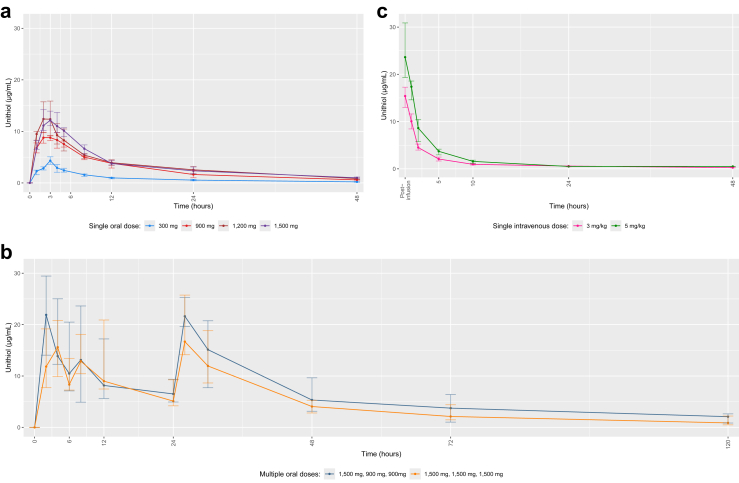
**Plasma concentrations of unithiol. a. Single oral ascending doses. b. Multiple oral ascending doses. c. Single intravenous ascending doses**. Median concentrations of unithiol over time for participants that received (a) single oral doses of unithiol (C1: 300 mg single oral [n = 4]; C2: 900 mg single oral [n = 4]; C3: 1200 mg single oral [n = 4]; C4: 1500 mg single oral [n = 4]), (b) multiple oral doses of unithiol (CM1: 1500 mg 0 h, 900 mg 6 h, 900 mg 24 h [n = 8]; CM2: 1500 mg 0 h, 1500 mg 6 h, 1500 mg 24 h [n = 8]) and (c) single intravenous doses of unithiol (CIV1: 3 mg/kg single intravenous [n = 4]; CIV2: 5 mg/kg single intravenous [n = 8]). The error bars represent the interquartile range at each time point.

During the interim analysis, the safety and pharmacokinetic data (Fig. 2 and Table 4), associated with the single ascending oral dose cohorts and the 3 mg/kg intravenous cohort were reviewed by the trial investigators. Due to challenges in conducting the pharmacokinetic analysis that risked delaying the multiple dose stage of the trial, the interim analysis was limited to four of eight participants from each cohort. The data from these 20 participants were reviewed by the DSMB and were approved as being sufficiently precise to inform the multiple dosing stage. The following two multiple-dose-regimens were proposed, and were subsequently ratified by the DSMB: CM1 (1500 mg at 0-h, 900 mg at 6-h, and 900 mg at 24-h); and CM2 (1500 mg at 0-h, 1500 mg at 6-h, and 1500 mg at 24-h). The highest tested loading dose (1500 mg), and an early 6-h timepoint for the second dose, were selected, as there was consensus that dosing should be front-loaded to provide greater plasma concentrations during the first 12-h of treatment, which is the period where the risk of venom associated local tissue damage and death is greatest.

**Table 4:** Unithiol pharmacokinetic parameters in the PK population.

a. Single oral ascending doses					
Cohort	C_max_ (μg/mL)	T_max_ (hour)	T_1/2_ (hour)	C_last_ (μg/mL)	AUC_0-∞_ (μg.h/mL)
C1 (n = 4)	4.3 (2.9–6.2)	3.0 (3.0–3.0)	13.9 (7.0–27.3)	0.2 (0.0–0.6)	44.1 (23.4–83.0)
C2 (n = 4)	10.2 (8.2–12.6)	2.8 (1.5–5.4)	12.3 (9.2–16.4)	0.6 (0.4–0.9)	138.4 (89.3–214.5)
C3 (n = 4)	13.4 (6.8–26.7)	1.9 (0.9–3.9)	14.1 (10.4–19.1)	0.7 (0.4–1.5)	171.8 (98.2–300.4)
C4 (n = 4)	14.7 (7.8–27.9)	2.9 (1.9–4.6)	18.4 (16.8–20.2)	1.0 (0.8–1.4)	204.5 (140.1–298.3)

b. Multiple oral ascending doses					
Cohort	C_max_ (μg/mL)	T_max_ (hour)	T_1/2_ (hour)	C_last_ (μg/mL)	AUC (μg.h/mL)
*Dose 1: 0–6-h*					*AUC*_*0*–*6h*_*(μg.h/mL)*
CM1 (n = 8)	19.7 (11.5–34.0)	2.2 (1.8–2.7)	–	11.8 (7.2–19.5)	79.5 (44.5–142.0)
CM2 (n = 8)	19.5 (12.2–31.2)	3.9 (3.0–5.0)	–	10.8 (6.3–18.5)	71.1 (49.6–102.0)
*Dose 2: 6–24-h*					*AUC*_*6*–*24h*_*(μg.h/mL)*
CM1 (n = 8)	13.8 (8.0–23.9)	8.5 (5.9–12.3)	–	6.3 (3.6–11.1)	160.9 (92.1–281.1)
CM2 (n = 8)	15.9 (8.5–29.6)	8.2 (6.6–10.3)	–	5.8 (3.5–9.7)	187.5 (104.7–335.7)
*Dose 3: >24-h*					*AUC*_*24*–*∞*_*(μg.h/mL)*
CM1 (n = 8)	20.6 (13.7–31.0)	26.5 (25.4–27.6)	29.3 (17.9–47.8)	1.2 (0.5–2.5)	582.9 (294.9–1152.0)
CM2 (n = 8)	18.2 (10.7–30.9)	27.0 (25.5–28.5)	41.0 (27.9–60.1)	1.1 (0.5–2.3)	509.6 (291.8–889.9)

c. Single intravenous ascending doses					
Cohort	C_5-min_ (μg/mL)	T_max_ (hour)	T_1/2_ (hour)	C_last_ (μg/mL)	AUC_0-∞_ (μg.h/mL)
CIV1 (n = 4)	13.7 (6.3–29.9)	–	15.8 (11.6–21.5)	0.3 (0.1–0.6)	63.7 (44.6–91.0)
CIV2 (n = 8)	23.5 (18.0–30.7)	–	7.4 (5.2–10.6)	0.5 (0.5–0.5)	82.2 (66.8–101.1)

C1: 300 mg single oral; C2: 900 mg single oral; C3: 1200 mg single oral; C4: 1500 mg single oral; CM1: 0-h/1500 mg oral, 6-h/900 mg oral, 24-h/900 mg oral; CM2: 0-h/1500 mg oral, 6-h/1500 mg oral, 24-h/1500 mg oral; CIV1: 3 mg/kg single intravenous; CIV2: 5 mg/kg single intravenous; C_5-min_: plasma concentration at 5-min after the initiation of the intravenous infusion; C_max_: maximum plasma concentration; T_max_: time to reach C_max_; T_1/2_: half-life; C_last_: last measurable plasma concentration; AUC_0–∞_: area under the curve from time zero extrapolated to infinity; AUC_0–6h_: area under the curve from time zero until 6 h after study drug administration; AUC_6–24h_: area under the curve from 6 h to 24 h after study drug administration; AUC_24–∞_: area under the curve from time 24 h after study drug administration extrapolated to infinity. Data are geometric mean, with the 95% confidence intervals for each parameter in parentheses.

### Multiple ascending oral doses

Two cohorts of eight participants each received the multiple oral dosing regimens (CM1 and CM2). There were no deaths, serious adverse events, or discontinuations. There were two solicited adverse events, which were both skin reactions of moderate severity (Table 3). There was one unsolicited adverse event with a possible causal relationship (Table 3), which was a moderate severity aphthous ulcer. There was one severe unsolicited event, a skin abscess that occurred 28 days after study drug administration, although this had no causal relationship. There were 15 occurrences of laboratory adverse events that affected six (38%) of the 16 participants, all of which were of mild severity (Table 3).

### Follow-up and further safety monitoring

All 64 participants underwent serial electrocardiogram monitoring, which did not identify any pathological abnormalities or any clinically significant changes in the Fridericia corrected QT interval (QTc) (Appendix, p 21). Female participants of childbearing potential underwent pregnancy testing and were asked to report any pregnancies; none occurred throughout the trial follow-up period. One participant from the CM2 cohort did not attend their scheduled face-to-face day-42 clinic visit, due to finding new employment and moving out of the study area. This participant did not have safety blood tests checked at day-42, but did engage with telephone follow-up at day-42 and at the end of study visit at 6-months, with no adverse events reported. All participants completed an end of study visit at 6-months, during which there were no ongoing adverse events.

### Pharmacokinetic analysis

The baseline characteristics of the population that contributed to the pharmacokinetic analysis (referred to as the PK population) is available in the Supplementary materials (Appendix, p 22). The standard 300 mg single dose of unithiol was associated with a geometric mean C_max_ of 4.3 μg/mL and AUC_0-∞_ of 44.1 μg.h/mL, whilst the maximum dose of 1500 mg had a C_max_ of 14.7 μg/mL and AUC_0-∞_ of 204.5 μg.h/mL (Fig. 2 and Table 4). The increase in C_max_ and area under the curve, with increasing single oral doses, appeared dose-proportional (Appendix, p 23–24). The geometric mean half-life for single oral dosing cohorts ranged between 12.3 and 18.4 h. The geometric mean T_max_ for the 1500 mg single oral dose was 2.9 h.

The CM1 and CM2 multiple oral dosing regimens were associated with a geometric mean C_max_ of 19.7 μg/mL and 19.5 μg/mL, respectively, following the first dose; 13.8 μg/mL and 15.9 μg/mL following the second dose; and 20.6 μg/mL and 18.2 μg/mL following the third dose (Table 4). The AUC_0–6h_ for CM1 and CM2 was 79.5 μg.h/mL and 71.1 μg.h/mL, respectively; the AUC_6–24h_ was 160.9 μg.h/mL and 187.5 μg.h/mL; and the AUC_24-∞_ was 582.9 μg.h/mL and 509.6 μg.h/mL.

The standard 3 mg/kg single intravenous dose was associated with a geometric mean C_5-min_ of 13.7 μg/mL and an AUC_0-∞_ of 63.7 μg.h/mL (Table 4). The 5 mg/kg single intravenous dose had a C_5-min_ of 23.5 μg/mL and an AUC_0-∞_ of 82.2 μg.h/mL.

Oral bioavailability was estimated at 60.7% when comparing the median AUC_0-∞_ between the 300 mg single oral dosing cohort and the 5 mg/kg single intravenous dosing cohort. These cohorts were selected because they both had a median administered dose of 300 mg. Oral bioavailability was also estimated for the 900 mg, 1200 mg and 1500 mg single oral dosing cohorts, which were similar, except for the 1500 mg cohort where oral bioavailability was lower at 49.6% (Appendix, p25).

### Selection of a phase 2 recommended dose

Following review of the safety and pharmacokinetic data, the CM1 regimen was selected as the phase 2 recommended dose. This regimen incorporates the maximum oral loading dose (1500 mg), to increase the probability of achieving early therapeutic plasma concentrations during the critical period immediately after a snakebite. The lower second and third doses (900 mg) were selected as the pharmacokinetic data suggest that gastrointestinal tract absorption of unithiol is saturated above this dose, as demonstrated by a limited difference in exposure between the CM1 and CM2 regimens (Table 4), as well as the lower oral bioavailability in the C4 cohort compared with the C1-3 cohorts (Appendix, p25). All underlying data are available on request via the repository, as detailed in the data sharing statement.

## Discussion

A short-course phase-2-recommended oral regimen has been developed, and shown to be well tolerated by a relevant population in a snakebite endemic region of Kenya. This oral regimen produced similar peak plasma concentrations to intravenous administration, thus potentially allowing prehospital delivery in the rural settings where snakebites tend to occur.

The median time to reach hospital following a snakebite in sub-Saharan Africa tends to be over 6-h,^10^^,^^36^ which, given a T_max_ of 2–3-h, suggests that orally administered unithiol could reach target plasma concentrations hours before antivenom administration for most patients. These first few hours are critical, as SVMP-induced coagulopathy has a rapid onset, and has usually manifested by the time a patient reaches hospital.^37^ Earlier administration of antivenom has been associated with improved clinical outcomes, including mortality.^38^^,^^39^

Increased oral doses of unithiol resulted in no safety concerns and were well tolerated in this trial. The most frequently occurring adverse events were abdominal pain, decreased appetite, skin reaction, and transaminitis, all of which spontaneously resolved and were of mild or moderate severity.

The pharmacokinetic analysis demonstrated that an oral loading dose of 1500 mg produced a C_max_ that was similar to that of a 3 mg/kg intravenous dose, suggesting that this oral dose could be a viable alternative in remote locations where intravenous administration of antivenom is not feasible. A further important finding from the pharmacokinetic analysis was the limited increase in exposure between the CM1 and CM2 regimens. The CM1 regimen was associated with slightly higher median unithiol plasma concentrations, but the overlapping error bars suggest there is no significant difference. Nevertheless, there is a suggestion that gastrointestinal absorption was saturated, and there is probably limited benefit of giving oral doses greater than that of the CM1 regimen.

The validity of our pharmacokinetic analysis is supported by our findings being similar to previously published data.^23^^,^^31^ A past study conducted in the USA, found that a single 300 mg oral dose of unithiol was associated with a C_max_ of 4.8 μg/mL; a T_max_ of 3.4 h; AUC_0–24h_ of 59.9 μg.h/mL; and a T_1/2_ of 9.9 h.^23^ These finding were similar to those from this TRUE-1 trial, which demonstrated a C_max_ of 4.3 μg/mL; a T_max_ of 3.0 h; AUC_0-∞_ of 44.1 μg.h/mL; and a t_1/2_ of 13.9 h. The longer half-life in TRUE-1 is probably due to the final sample collection for the pharmacokinetic analysis being at 48-h, whereas it was 24-h in the previous study.

The 1500 mg loading dose is 10-fold higher than the human equivalent dose of oral unithiol that demonstrated a significant reduction in mortality in a murine model of envenoming.^14^^,^^40^ The IC_50_ of unithiol that inhibits SVMP activity *in vitro* depends on the concentration and type of venom, and has recently been reported to range between 0.11 μg/mL and 1.35 μg/mL for the following geographically diverse snake species: *Bitis arietans*, *Bothrops jararaca*, *Crotalus atrox*, *Calloselasma rhodostoma*, *Dispholidus typus*, and *Echis ocellatus*.^41^^,^^42^ All participants in the CM1 group had plasma concentrations of unithiol, from 2-h until 48-h after administration, that exceeded 1.35 μg/mL, suggesting that the phase 2 recommended dose produces sufficient plasma concentrations to inhibit circulating SVMP enzymes.

Unlike antibody-based therapeutics, which are innately specific, unithiol is able to inhibit SVMP enzymes from globally diverse snake species, including *Echis*, *Bothrops*, *Bitis*, *Crotalus*, *Daboia*, *Calloselasma* and *Dispholidus* spp.^14^^,^^20^^,^^41^^,^^43^ This pan–species activity is due to unithiol targeting the zinc ion, which is a ubiquitous component of SVMP enzymes.^44^ Although structural analysis of the interaction specifically between unithiol and SVMP enzymes has not been undertaken, it is known that chelating agents act to remove the zinc ion from the active site of metalloproteinase enzymes,^45^ which neutralises their activity. An additional potential benefit of this small molecule drug for snakebite, is the prediction of superior tissue penetration when compared to that of antivenom antibodies,^46^ suggesting that unithiol may be more effective at preventing morbidity due to local envenoming.

The multiple oral dosing regimen developed in this trial differs from the standard oral regimen in that the loading dose is higher (1500 mg rather than 100–300 mg^16^^,^^23^) and the course duration is shorter (24-h rather than weeks-months^24^). Front-loading of the dose achieves high plasma concentrations during the critical period early after a snakebite. A short duration reduces the risk of certain adverse events that tend to occur with repeated doses, including Stevens-Johnson syndrome.^47^^,^^48^ The optimal duration of treatment with unithiol for snakebite envenoming will need to be confirmed in future clinical studies. Given that a single dose of antivenom usually induces a rapid and sustained resolution of venom induced consumption coagulopathy in *Echis* and *Bothrops* spp. envenoming,^49^^,^^50^ it is feasible for a short-course of unithiol to be efficacious, although differences in the pharmacokinetic–pharmacodynamic profile of antibody and small molecule-based treatments are uncertain.

A limitation of this trial was that only total unithiol plasma concentrations were calculated, which includes unithiol in its oxidised forms. A previous pharmacokinetic analysis, in healthy volunteers that received a 3 mg/kg intravenous dose, demonstrated that plasma concentrations of unaltered unithiol decline rapidly, with a half-life of 1.8-h.^31^ The two sulfhydryl (thiol) groups of unithiol, which are the active chelating components, are prone to oxidation through the formation of disulfide bonds, and thus the drug will be inactive in this form.^22^ However, animal data have demonstrated that oxidised unithiol is reduced *in vivo* back to its active form.^51^ Further study of the pharmacodynamic activity of unithiol is required to understand the potential role of the oxidised form, which will influence the duration of effect after dose administration. An exploratory objective of this trial was to develop and apply a pharmacodynamic endpoint. The assays were based on the addition of a concentration of venom to plasma samples *ex vivo*, and measurement of the degree of inhibition of SVMP activity across plasma samples with different concentrations of unithiol. Two assay formats were adapted from previous *in vitro* assays; the first sought to measure inhibition of SVMP driven hydrolysis of a fluorescent substrate, whilst the second aimed to measure inhibition of SVMP procoagulant effect on plasma. These techniques failed to provide a reliable measure of SVMP activity during the assay development stage, leading to this exploratory objective being abandoned.

Further limitations to this trial include that safety has only been assessed in a small number of participants, and that female participants in particular were poorly represented, due to the protocol requirement necessitating the use of a reliable method of contraception. This trial was open label, which may have influenced participants’ reporting of adverse events. Further safety data should be collected in future clinical studies evaluating higher oral doses. The pharmacokinetic parameters of unithiol in healthy volunteers may differ in people with snakebite, due to venom-induced physiological changes in kidney function and capillary permeability. This trial only included adults and it will be important to recruit children, who are frequently affected by snakebite,^10^ into future clinical trials.

This clinical trial sits along a targeted programme of translational research to screen, optimise, and clinically assess small molecule therapeutics for snakebite.^52^ The efficacy of a metal chelator in inhibiting venom-induced toxicity was demonstrated in 1964,^53^ yet challenges, including limited funding,^54^ have stifled clinical development. There is an urgent need to develop global partnerships, incorporate modern trial methodologies, and establish an ethical framework for conducting clinical trials of novel snakebite therapeutics. The recently established global core outcome measurement set will support the standardised reporting of clinical trial outcomes for broadly acting therapeutics, such as unithiol, across diverse snake species and geographic settings.^55^

This study has demonstrated that a high-dose short-course regimen of oral unithiol is well tolerated in a relevant population, and should be progressed to phase 2 clinical trials. Future trials should be based in low-middle income settings, where the greatest burden of snakebite exists. Trials based in settings where the biting species have venom that is primarily composed of SVMP enzymes, such as West Africa and South America, may consider exploring unithiol as an alternative to antivenom. Unithiol should also be evaluated as a prehospital administered adjunct to antivenom and other novel therapeutics. It is hoped that this trial marks the beginning of an era of modernised therapeutics in the field of snakebite, which will be essential to achieving the World Health Organization target of reducing snakebite associated morbidity and mortality by 50%.

## Contributors

Michael Abouyannis (conceptualisation, formal analysis, funding acquisition, methodology, project administration, and writing—original draft), Yvonne K. Nyambura (project administration), Samson Ngome (project administration), Debra Riako (project administration), Jennifer Musyoki (project administration), Charles Muiruri (data curation, and formal analysis), Benedict Orindi (data curation, formal analysis, and writing—review & editing), Laura Else (investigation), Alieu Amara (investigation), Laura Dickinson (formal analysis), Rachel H. Clare (investigation), Laura-Oana Albulescu (investigation), Adam P. Westhorpe (investigation), Jeroen Kool (conceptualisation), Ifedayo Adetifa (supervision, and writing—review & editing), Francis M. Ndungu (supervision, and writing—review & editing), Richard FitzGerald (conceptualisation, methodology, and writing—review & editing), Saye Khoo (funding acquisition, methodology, and writing—review & editing), David G. Lalloo (conceptualisation, funding acquisition, methodology, supervision, and writing—review & editing), Nicholas R. Casewell (conceptualisation, funding acquisition, methodology, supervision, and writing—review & editing), and Mainga Hamaluba (conceptualisation, funding acquisition, methodology, project administration, supervision, and writing—review & editing).

All authors read and approved the final version of the manuscript. The authors MA, CM, and BO accessed and verified the underlying data.

## Data sharing statement

The data management plan, DSMB charter, statistical analysis plan, pharmacokinetic analysis plan, patient information leaflet and consent form are available via the Harvard Dataverse KWTRP Research Data Repository. The underlying data are available via this repository on request: https://dataverse.harvard.edu/dataset.xhtml?persistentId=doi:10.7910/DVN/LJOYSO.

## Declaration of interests

MA, YKN, SN, DR, JM, CM, BO, LE, AA, LD, RHC, APW, IA, FMN, RF, SK, DGL, MH all declare no conflicts of interest. LOA, JK and NRC are named inventors on a patent application relating to the use of unithiol for snakebite indication. The manufacturer (Heyl Chemisch-pharmazeutische Fabrik GmbH & Co. KG) provided the study drug without charge for the purposes of this trial. The manufacturer had no role in the study design, data analysis, or the decision to publish the results.

## Appendix A Supplementary data

Supplementary Material

TRUE1_published_protocol
