# Supplementary material for: Development of an oral regimen of unithiol for the treatment of snakebite envenoming: a phase 1 open-label dose-escalation safety trial and pharmacokinetic analysis in healthy Kenyan adults
Source: eBioMedicine. 2025 Feb 27;113:105600. doi: 10.1016/j.ebiom.2025.105600 (PMC11919382; doi:10.1016/j.ebiom.2025.105600)
Supplement: Supplementary Material [file mmc1.docx]

## Appendix

### Dose escalation holding criteria

If any of the following scenarios occur within a group with reasonable possibility of a causal relationship with unithiol, dose escalation will be stopped pending safety review, prior to higher dose levels being evaluated. In the case a serious adverse event occurring, this will be reported in accordance with section 11.7 of this protocol, including reporting to the Sponsor and the DSMB within 48 hours.

- Clinically relevant signs or symptoms or intolerable adverse events of similar nature occur in 2 or more subjects in a group that in the opinion of the Investigator warrant stopping of dose escalation.
- One or more subjects report a serious adverse event considered by the Investigator to be related to the study drug.
- Two or more severe adverse events occur which are considered by the Investigator to be related to the study drug.
- Dose escalations will only occur after a minimum period of days, depending on the dose received, as defined in the published protocol.

### Eligibility criteria

### Inclusion criteria

- Capable of giving informed consent
- Male or female
- KHDSS resident
- 18-64 years old (inclusive)
- Body weight 50-120kg
- In good health, as determined by the investigator following medical history, drug history, examination, vital signs, ECG and blood tests
- Willing to be admitted to the in-patient facility for up to 5 days for dosing and intensive blood sampling
- On effective contraception as defined by: Use of effective method of contraception for duration of study (women only). We will ask the female volunteers to come with their family planning records to verify. Effective contraception is defined as a contraceptive method with failure rate of less than 1% per year when used consistently and correctly, in accordance with the product label. Examples of these include: combined oral contraceptives; injectable progestogen; implants of etenogestrel or levonorgestrel; intrauterine device or intrauterine system; male partner sterilisation at least 6 months prior to the female subject’s entry into the study, and the relationship is monogamous; male condom combined with a vaginal spermicide (foam, gel, film, cream or suppository); and male condom combined with a female diaphragm, either with or without a vaginal spermicide (foam, gel, film, cream, or suppository).

### Exclusion criteria

- Prescribed a concomitant medication other than paracetamol or an appropriate contraceptive, which in the opinion of the Investigator warrants exclusion.
- Any significant current or past history of cardiovascular, respiratory, renal or hepatic disease.
- Subjects who have taken any non-prescribed herbal medication or mineral supplement in the preceding 7 days, which in the opinion of the Investigator warrants exclusion.
- Subject with clinically significant abnormal vital signs at screening
- Abnormal laboratory findings deemed significant by the Investigator
- Subjects who are pregnant or lactating
- Decline pre-trial screening, including HIV testing
- HIV positive subjects will be excluded from the trial and, if not already receiving appropriate clinic follow-up, would be referred to a government clinic for ongoing care
- Subjects with asthma (due to possible risk of exacerbation with allergic type skin reactions to unithiol)
- Subjects that have donated blood within the past 3 months
- Subjects who in the opinion of the investigator should not participate

### Pre-dose exclusion Criteria

Following pre-dose assessments, subjects may be excluded from the dosing cohort for the following reasons. This exclusion may be temporary, and the subject could join a later dosing cohort if the Investigator identifies an abnormality that is likely to resolve within an acceptable period of time, such as an acute infection.

- Clinically significant vital signs or 12 lead ECG findings
- Clinically significant abnormal laboratory findings
- Intercurrent illness
- Deviation from study restrictions (unless in the opinion of the Investigator these deviations will not interfere with the study procedures, compromise safety or affect the study results. Any such deviations will be recorded in the source data and documented in the TMF)

## List of adverse events on the Dimaval^®^ summary of product characteristics

| Symptoms | Abdominal pain  Decreased appetite  Dysgeusia (change in taste)  Injection site pain  Muscular weakness  Nausea |
| --- | --- |
| Physical observations | Fever  Hypotension (with intravenous unithiol)  Shivering  Skin reaction (itching rash)  Stevens-Johnson’s syndrome |
| Laboratory abnormalities | Blood creatinine increased  Leukopaenia  Transaminitis |

## Timing of pharmacokinetic blood sampling

| **Dosing cohorts** | **Time** |
| --- | --- |
| C1, C2, C3, C4 | Pre-dose, 1h, 2h, 3h, 4h, 5h, 8h, 12h, 24h, and 48h |
| CIV1, CIV2 | Pre-dose, end-of-infusion, 1h, 2h, 5h, 10h, 24h, and 48h |
| CM1, CM2 | Pre-dose, 2-3h, 4h, 6h, 8-9h, 12-14h, 20-24h, 26-27h, 30-34h, 48h, 72h, and 120h |

## Reasons for ineligibility at screening

| **Eligibility criteria** | **Number** |
| --- | --- |
| Not healthy by medical history, physical examination, or laboratory findings | 48 |
| Body weight outside of 50-120 kg | 11 |
| Any medical, social condition, or occupational reason that, in the judgment of the investigator, was a contraindication to the protocol or could have interfered with the study outcomes or posed a threat to the participant's health | 7 |
| Seropositive for HIV, hepatitis B, or hepatitis C | 6 |
| Female participant not on effective contraception | 4 |
| Not resident in the study area during the trial period | 3 |
| Abnormal laboratory findings deemed significant by the investigator | 3 |
| Prescribed a concomitant medication other than paracetamol or an appropriate contraceptive | 2 |
| Taken any non-prescribed herbal medication or mineral supplement in the last 3 months | 1 |

## All adverse events following administration of unithiol in the safety population

|  | **Single oral dose** | | | | **-** | **Multiple oral dose** | | **-** | **Single intravenous dose** | | **-** |
| --- | --- | --- | --- | --- | --- | --- | --- | --- | --- | --- | --- |
|  | **C1 (n=8)** | **C2 (n=8)** | **C3 (n=8)** | **C4 (n=8)** | **Total (n=32)** | **CM1 (n=8)** | **CM2 (n=8)** | **Total (n=16)** | **CIV1 (n=8)** | **CIV2 (n=8)** | **Total (n=16)** |
| **Number of SAEs** | 0 | 0 | 0 | 0 | **0** | 0 | 0 | **0** | 0 | 0 | **0** |
| **Number of deaths** | 0 | 0 | 0 | 0 | **0** | 0 | 0 | **0** | 0 | 0 | **0** |
| **No. of solicited AEs** | 16 | 11 | 8 | 4 | **39** | 1 | 1 | **2** | 15 | 5 | **20** |
| **No. of Unsolicited AEs** | 2 | 4 | 5 | 2 | **13** | 1 | 0 | **1** | 3 | 0 | **3** |
| **No. of Laboratory AEs** | 7 | 9 | 5 | 2 | **23** | 7 | 8 | **15** | 5 | 12 | **17** |
| **Solicited AEs [1]** |  |  |  |  |  |  |  |  |  |  |  |
| Abdominal pain | 3  (19%, 7–43) | 2  (18%, 5–48) | 2  (25%, 7–59) | 1  (25%, 5–70) | **8**  **(25%, 13–42)** | 0  (0%, 0–79) | 0  (0%, 0–79) | **0**  **(0%, 0–65)** | 4  (27%, 11–52) | 1  (20%, 4–62) | **5**  **(25%, 11–47)** |
| Dysgeusia | 2  (13%, 3–36) | 0  (0%, 0–26) | 0  (0%, 0–32) | 0  (0%, 0–50) | **2**  **(6%, 2–20)** | 0  (0%, 0–79) | 0  (0%, 0–79) | **0**  **(0%, 0–65)** | 1  (7%, 1–30) | 1  (20%, 4–62) | **2**  **(10%, 3–30)** |
| Fever | 1  (6%, 1–28) | 0  (0%, 0–26) | 0  (0%, 0–32) | 1  (25%, 5–70) | **2**  **(6%, 2–20)** | 0  (0%, 0–79) | 0  (0%, 0–79) | **0**  **(0%, 0–65)** | 0  (0%, 0–20) | 0  (0%, 0–43) | **0**  **(0%, 0–16)** |
| Injection site pain | - | - | - | - | **-** | - | - | **-** | 2  (13%, 4–38) | 0  (0%, 0–43) | **2**  **(10%, 3–30)** |
| Decreased appetite | 4  (25%, 10–49) | 1  (9%, 2–38) | 1  (13%, 2–47) | 0  (0%, 0–50) | **6**  **(19%, 9–35)** | 0  (0%, 0–79) | 0  (0%, 0–79) | **0**  **(0%, 0–65)** | 3  (20%, 7–45) | 1  (20%, 4–62) | **4**  **(20%, 8–42)** |
| Nausea | 2  (13%, 3–36) | 1  (9%, 2–38) | 2  (25%, 7–59) | 1  (25%, 5–70) | **6**  **(19%, 9–35)** | 0  (0%, 0–79) | 0  (0%, 0–79) | **0**  **(0%, 0–65)** | 1  (7%, 1–30) | 0  (0%, 0–43) | **1**  **(5%, 1–24)** |
| Shivering | 1  (6%, 1–28) | 1  (9%, 2–38) | 0  (0%, 0–32) | 0  (0%, 0–50) | **2**  **(6%, 2–20)** | 0  (0%, 0–79) | 0  (0%, 0–79) | **0**  **(0%, 0–65)** | 0  (0%, 0–20) | 0  (0%, 0–43) | **0**  **(0%, 0–16)** |
| Skin reaction | 0  (0%, 0–19) | 5  (45%, 21–72) | 2  (25%, 7–59) | 0  (0%, 0–50) | **7**  **(22%, 11–39)** | 1  (100%, 21–100) | 1  (100%, 21–100) | **2**  **(100%, 34–100)** | 3  (20%, 7–45) | 1  (20%, 4–62) | **4**  **(20%, 8–42)** |
| Muscular weakness | 3  (19%, 7–43) | 1  (9%, 2–38) | 1  (13%, 2–47) | 1  (25%, 5–70) | **6**  **(19%, 9–35)** | 0  (0%, 0–79) | 0  (0%, 0–79) | **0**  **(0%, 0–65)** | 1  (7%, 1–30) | 1  (20%, 4–62) | **2**  **(10%, 3–30)** |
| Hypotension^†^ | - | - | - | - | **-** | - | - | **-** | 0  (0%, 0–20) | 0  (0%, 0–43) | **0**  **(0%, 0–16)** |
| SJS | 0  (0%, 0–19) | 0  (0%, 0–26) | 0  (0%, 0–32) | 0  (0%, 0–50) | **0**  **(0%, 0–11)** | 0  (0%, 0–79) | 0  (0%, 0–79) | **0**  **(0%, 0–65)** | 0  (0%, 0–20) | 0  (0%, 0–43) | **0**  **(0%, 0–16)** |
| **Unsolicited AEs**^††^**[2]** |  |  |  |  |  |  |  |  |  |  |  |
| Aphthous ulcer | 0  (0%, 0–66) | 0  (0%, 0–49) | 0  (0%, 0–43) | 0  (0%, 0–66) | **0**  **(0%, 0–23)** | 1  (100%, 21–100) | 0 | **1**  **(100%, 21–100)** | 0  (0%, 0–56) | 0 | **0**  **(0%, 0–56)** |
| Arthralgia | 1  (50%, 9–90) | 0  (0%, 0–49) | 0  (0%, 0–43) | 0  (0%, 0–66) | **1**  **(8%, 1–33)** | 0  (0%, 0–79) | 0 | **0**  **(0%, 0–79)** | 0  (0%, 0–56) | 0 | **0**  **(0%, 0–56)** |
| Catheter site pain | 0  (0%, 0–66) | 0  (0%, 0–49) | 1  (20%, 4–62) | 0  (0%, 0–66) | **1**  **(8%, 1–33)** | 0  (0%, 0–79) | 0 | **0**  **(0%, 0–79)** | 0  (0%, 0–56) | 0 | **0**  **(0%, 0–56)** |
| Dry mouth | 0  (0%, 0–66) | 0  (0%, 0–49) | 1  (20%, 4–62) | 0  (0%, 0–66) | **1**  **(8%, 1–33)** | 0  (0%, 0–79) | 0 | **0**  **(0%, 0–79)** | 0  (0%, 0–56) | 0 | **0**  **(0%, 0–56)** |
| Gastroenteritis viral | 0  (0%, 0–66) | 0  (0%, 0–49) | 0  (0%, 0–43) | 1  (50%, 9–90) | **1**  **(8%, 1–33)** | 0  (0%, 0–79) | 0 | **0**  **(0%, 0–79)** | 3  (100%, 44–100) | 0 | **3**  **(100%, 44–100)** |
| Headache | 0  (0%, 0–66) | 1  (25%, 4–70) | 1  (20%, 4–62) | 0  (0%, 0–66) | **2**  **(15%, 4–42)** | 0  (0%, 0–79) | 0 | **0**  **(0%, 0–79)** | 0  (0%, 0–56) | 0 | **0**  **(0%, 0–56)** |
| Heavy menstrual bleeding | 0  (0%, 0–66) | 0  (0%, 0–49) | 1  (20%, 4–62) | 0  (0%, 0–66) | **1**  **(8%, 1–33)** | 0  (0%, 0–79) | 0 | **0**  **(0%, 0–79)** | 0  (0%, 0–56) | 0 | **0**  **(0%, 0–56)** |
| Hypoaesthesia | 0  (0%, 0–66) | 1  (25%, 4–70) | 0  (0%, 0–43) | 0  (0%, 0–66) | **1**  **(8%, 1–33)** | 0  (0%, 0–79) | 0 | **0**  **(0%, 0–79)** | 0  (0%, 0–56) | 0 | **0**  **(0%, 0–56)** |
| Panic attack | 1  (50%, 9–90) | 0  (0%, 0–49) | 0  (0%, 0–43) | 1  (50%, 9–90) | **2**  **(15%, 4–42)** | 0  (0%, 0–79) | 0 | **0**  **(0%, 0–79)** | 0  (0%, 0–56) | 0 | **0**  **(0%, 0–56)** |
| Viral infection | 0  (0%, 0–66) | 1  (25%, 4–70) | 0  (0%, 0–43) | 0  (0%, 0–66) | **1**  **(8%, 1–33)** | 0  (0%, 0–79) | 0 | **0**  **(0%, 0–79)** | 0  (0%, 0–56) | 0 | **0**  **(0%, 0–56)** |
| Viral upper respiratory tract infection | 0  (0%, 0–66) | 1  (25%, 4–70) | 1  (20%, 4–62) | 0  (0%, 0–66) | **2**  **(15%, 4–42)** | 0  (0%, 0–79) | 0 | **0**  **(0%, 0–79)** | 0  (0%, 0–56) | 0 | **0**  **(0%, 0–56)** |
| **Laboratory AEs [3]** |  |  |  |  |  |  |  |  |  |  |  |
| Transaminitis | 0  (0%, 0–35) | 6  (67%, 35–88) | 0  (0%, 0–43) | 0  (0%, 0–66) | **6**  **(26%, 12–46)** | 0  (0%, 0–35) | 0  (0%, 0–32) | **0**  **(0%, 0–20)** | 2  (40%, 12–77) | 2  (17%, 5–45) | **4**  **(23%, 9–47)** |
| Haemoglobin decreased | 1  (14%, 2–51) | 0  (0%, 0–30) | 0  (0%, 0–43) | 1  (50%, 9–91) | **2**  **(9%, 2–27)** | 4  (57%, 25–84) | 2  (25%, 7–59) | **6**  **(40%, 20–64)** | 0  (0%, 0–43) | 0  (0%, 0–24) | **0**  **(0%, 0–18)** |
| Blood creatinine increased | 1  (14%, 2–51) | 0  (0%, 0–30) | 1  (20%, 4–62) | 0  (0%, 0–66) | **2**  **(9%, 2–27)** | 0  (0%, 0–35) | 0  (0%, 0–32) | **0**  **(0%, 0–20)** | 0  (0%, 0–43) | 0  (0%, 0–24) | **0**  **(0%, 0–18)** |
| Hyperkalaemia | 0  (0%, 0–35) | 0  (0%, 0–30) | 0  (0%, 0–43) | 0  (0%, 0–66) | **0**  **(0%, 0–14%)** | 0  (0%, 0–35) | 0  (0%, 0–32) | **0**  **(0%, 0–20)** | 0  (0%, 0–43) | 1  (8%, 1–35) | **1**  **(6%, 1–27)** |
| Hypernatraemia | 0  (0%, 0–35) | 2  (22%, 6–55) | 3  (60%, 23–88) | 1  (50%, 9–91) | **6**  **(26%, 12–46)** | 0  (0%, 0–35) | 0  (0%, 0–32) | **0**  **(0%, 0–20)** | 0  (0%, 0–43) | 0  (0%, 0–24) | **0**  **(0%, 0–18)** |
| Leukopaenia | 2  (29%, 8–65) | 0  (0%, 0–30) | 0  (0%, 0–43) | 0  (0%, 0–66) | **2**  **(9%, 2–27)** | 1  (14%, 2–51) | 3  (37%, 14–69) | **4**  **(27%, 11–52)** | 1  (20%, 4–62) | 3  (25%, 9–53) | **4**  **(23%, 9–47)** |
| Lymphopaenia | 2  (29%, 8–65) | 0  (0%, 0–30) | 0  (0%, 0–43) | 0  (0%, 0–66) | **2**  **(9%, 2–27)** | 2  (28%, 8–64) | 3  (37%, 14–69) | **5**  **(33%, 15–52)** | 1  (20%, 4–62) | 3  (25%, 9–53) | **4**  **(23%, 9–47)** |
| Neutropaenia | 0  (0%, 0–35) | 1  (11%, 2–43) | 1  (20%, 4–62) | 0  (0%, 0–66) | **2**  **(9%, 2–27)** | 0  (0%, 0–35) | 0  (0%, 0–32) | **0**  **(0%, 0–20)** | 1  (20%, 4–62) | 0  (0%, 0–24) | **1**  **(6%, 1–27)** |
| Thrombocytopaenia | 1  (14%, 2–51) | 0  (0%, 0–30) | 0  (0%, 0–43) | 0  (0%, 0–66) | **1**  **(4%, 1–21)** | 0  (0%, 0–35) | 0  (0%, 0–32) | **0**  **(0%, 0–20)** | 0  (0%, 0–43) | 3  (25%, 9–53) | **3**  **(18%, 6–41)** |

† Hypotension occurring within 15-minutes of intravenous administration of the study drug.

†† Only unsolicited adverse events with a possible, probable, or definite causal relationship are listed.

[1] Percentages are based on number of solicited AEs for each group.

[2] Percentages are based on number of unsolicited AEs for each group.

[3] Percentages are based on number of laboratory AEs for each group.

AE: adverse events; SAE: serious adverse events; SJS: Stevens-Johnson’s syndrome; dash (-): not assessed for those particular groups.

## Line listing of solicited adverse events

| **Cohort** | **Gender** | **Date of study drug administration** | **Solicited adverse event** | **Onset date** | **Serious** | **Severity** | **Relationship** |
| --- | --- | --- | --- | --- | --- | --- | --- |
| C1 | Male | 02/03/2022 | Abdominal pain | 07/03/2022 | No | Mild | Probable |
| C1 | Male | 02/03/2022 | Abdominal pain | 16/05/2022 | No | Mild | Possible |
| C1 | Male | 02/03/2022 | Abdominal pain | 02/03/2022 | No | Moderate | Definite |
| C1 | Male | 02/03/2022 | Change in taste | 02/03/2022 | No | Mild | Definite |
| C1 | Male | 02/03/2022 | Change in taste | 03/03/2022 | No | Mild | Definite |
| C1 | Male | 02/03/2022 | Fever | 08/03/2022 | No | Mild | Probable |
| C1 | Male | 02/03/2022 | Loss of appetite | 02/03/2022 | No | Mild | Definite |
| C1 | Male | 02/03/2022 | Loss of appetite | 03/03/2022 | No | Mild | Definite |
| C1 | Male | 02/03/2022 | Loss of appetite | 08/03/2022 | No | Mild | Probable |
| C1 | Male | 02/03/2022 | Loss of appetite | 16/05/2022 | No | Mild | Possible |
| C1 | Male | 02/03/2022 | Nausea | 08/03/2022 | No | Mild | Probable |
| C1 | Male | 02/03/2022 | Nausea | 16/05/2022 | No | Mild | Possible |
| C1 | Male | 02/03/2022 | Shivering | 08/03/2022 | No | Mild | Probable |
| C1 | Male | 02/03/2022 | Weakness | 02/03/2022 | No | Mild | Probable |
| C1 | Male | 02/03/2022 | Weakness | 07/03/2022 | No | Mild | Probable |
| C1 | Male | 02/03/2022 | Weakness | 16/05/2022 | No | Mild | Possible |
| C2 | Male | 09/03/2022 | Abdominal pain | 09/03/2022 | No | Mild | Definite |
| C2 | Male | 09/03/2022 | Abdominal pain | 17/04/2022 | No | Mild | Possible |
| C2 | Male | 09/03/2022 | Loss of appetite | 10/03/2022 | No | Mild | Probable |
| C2 | Male | 09/03/2022 | Nausea | 10/03/2022 | No | Mild | Probable |
| C2 | Male | 09/03/2022 | Shivering | 10/03/2022 | No | Mild | Probable |
| C2 | Male | 09/03/2022 | Skin reaction | 01/04/2022 | No | Mild | Possible |
| C2 | Male | 09/03/2022 | Skin reaction | 10/03/2022 | No | Mild | Definite |
| C2 | Male | 09/03/2022 | Skin reaction | 16/03/2022 | No | Mild | Possible |
| C2 | Male | 09/03/2022 | Skin reaction | 16/04/2022 | No | Mild | Possible |
| C2 | Male | 08/03/2022 | Skin reaction | 27/03/2022 | No | Mild | Possible |
| C2 | Male | 09/03/2022 | Weakness | 10/03/2022 | No | Mild | Possible |
| C3 | Female | 23/03/2022 | Abdominal pain | 12/09/2022 | No | Mild | Definite |
| C3 | Female | 23/03/2022 | Abdominal pain | 29/03/2022 | No | Mild | Probable |
| C3 | Female | 23/03/2022 | Loss of appetite | 26/03/2022 | No | Mild | Probable |
| C3 | Female | 16/03/2022 | Nausea | 16/03/2022 | No | Mild | Probable |
| C3 | Female | 23/03/2022 | Nausea | 27/03/2022 | No | Mild | Probable |
| C3 | Male | 23/03/2022 | Skin reaction | 23/04/2022 | No | Mild | No relationship |
| C3 | Female | 23/03/2022 | Skin reaction | 24/03/2022 | No | Mild | Probable |
| C3 | Female | 23/03/2022 | Weakness | 29/03/2022 | No | Mild | Probable |
| C4 | Female | 06/04/2022 | Abdominal pain | 06/04/2022 | No | Mild | Definite |
| C4 | Female | 06/04/2022 | Fever | 16/05/2022 | No | Mild | Possible |
| C4 | Female | 06/04/2022 | Nausea | 08/04/2022 | No | Mild | Probable |
| C4 | Female | 06/04/2022 | Weakness | 16/05/2022 | No | Mild | Possible |
| CM1 | Male | 19/01/2023 | Skin reaction | 14/06/2023 | No | Moderate | Unlikely |
| CM2 | Female | 02/02/2023 | Skin reaction | 04/02/2023 | No | Moderate | Definite |
| CIV1 | Female | 16/03/2022 | Abdominal pain | 17/03/2022 | No | Mild | Definite |
| CIV1 | Male | 16/03/2022 | Abdominal pain | 18/03/2022 | No | Mild | Probable |
| CIV1 | Female | 16/03/2022 | Abdominal pain | 21/03/2022 | No | Mild | Probable |
| CIV1 | Male | 16/03/2022 | Abdominal pain | 23/04/2022 | No | Moderate | Possible |
| CIV1 | Female | 16/03/2022 | Change in taste | 17/03/2022 | No | Mild | Possible |
| CIV1 | Male | 16/03/2022 | Loss of appetite | 17/03/2022 | No | Mild | Probable |
| CIV1 | Female | 16/03/2022 | Loss of appetite | 17/03/2022 | No | Mild | Possible |
| CIV1 | Female | 16/03/2022 | Loss of appetite | 21/03/2022 | No | Mild | Probable |
| CIV1 | Female | 16/03/2022 | Nausea | 17/03/2022 | No | Mild | Definite |
| CIV1 | Male | 16/03/2022 | Painful injection site | 16/03/2022 | No | Mild | Definite |
| CIV1 | Female | 16/03/2022 | Painful injection site | 17/03/2022 | No | Mild | Definite |
| CIV1 | Male | 16/03/2022 | Skin reaction | 16/03/2022 | No | Mild | Definite |
| CIV1 | Male | 16/03/2022 | Skin reaction | 16/03/2022 | No | Mild | Probable |
| CIV1 | Male | 16/03/2022 | Skin reaction | 07/04/2022 | No | Moderate | Possible |
| CIV1 | Female | 16/03/2022 | Weakness | 17/03/2022 | No | Mild | Possible |
| CIV2 | Female | 24/01/2023 | Abdominal pain | 24/01/2023 | No | Mild | Definite |
| CIV2 | Female | 24/01/2023 | Change in taste | 25/01/2023 | No | Mild | Definite |
| CIV2 | Female | 24/01/2023 | Loss of appetite | 25/01/2023 | No | Mild | Definite |
| CIV2 | Male | 24/01/2023 | Skin reaction | 09/02/2023 | No | Moderate | Definite |
| CIV2 | Female | 24/01/2023 | Weakness | 25/01/2023 | No | Mild | Definite |

The numbers of solicited adverse events that were reported at each study visit have been presented. A solicited adverse event that was reported at multiple timepoints during a single day (for example, nausea occurring at 3-hours post-dose and 4-hours post-dose in a single participant) has been reported as a single solicited adverse event. Events that were continuous over several days (e.g., nausea occurring on day-2 and continuing until day-5) have been reported as separate solicited adverse events (e.g., one nausea event on day-2 and a second nausea event on day-5).

## Line listing of unsolicited adverse events

| **Cohort** | **Gender** | **Date of study drug administration** | **Unsolicited adverse event** | **Onset date** | **Resolution date** | **Ongoing** | **Serious** | **Severity** | **Relationship** |
| --- | --- | --- | --- | --- | --- | --- | --- | --- | --- |
| C1 | Male | 02/03/2022 | Arthralgia | 08/03/2022 | 10/03/2022 | No | No | Mild | Possible |
| C1 | Male | 02/03/2022 | Gastritis | 07/03/2022 | 07/03/2022 | No | No | Mild | No relationship |
| C1 | Male | 02/03/2022 | Gastritis | 16/05/2022 | 30/05/2022 | No | No | Moderate | No relationship |
| C1 | Male | 02/03/2022 | Panic attack | 02/03/2022 | 02/03/2022 | No | No | Mild | Possible |
| C1 | Male | 02/03/2022 | Urinary tract infection bacterial | 07/03/2022 | 15/03/2022 | No | No | Moderate | No relationship |
| C1 | Male | 02/03/2022 | Viral upper respiratory tract infection | 21/03/2022 | 28/03/2022 | No | No | Moderate | Unlikely |
| C2 | Male | 09/03/2022 | Aphthous ulcer | 15/04/2022 | 24/04/2022 | No | No | Moderate | Unlikely |
| C2 | Male | 09/03/2022 | Arthralgia | 16/04/2022 | 25/04/2022 | No | No | Moderate | Unlikely |
| C2 | Male | 09/03/2022 | Headache | 09/03/2022 | 10/03/2022 | No | No | Mild | Possible |
| C2 | Male | 09/03/2022 | Hypoaesthesia | 12/03/2022 | 14/03/2022 | No | No | Mild | Possible |
| C2 | Male | 08/03/2022 | Soft tissue injury | 15/04/2022 | 20/04/2022 | No | No | Mild | No relationship |
| C2 | Male | 09/03/2022 | Viral infection | 10/03/2022 | 11/03/2022 | No | No | Mild | Probable |
| C2 | Male | 09/03/2022 | Viral upper respiratory tract infection | 10/03/2022 | 12/03/2022 | No | No | Mild | Possible |
| C2 | Male | 09/03/2022 | Viral upper respiratory tract infection | 13/04/2022 | 20/04/2022 | No | No | Mild | No relationship |
| C3 | Male | 23/03/2022 | Acarodermatitis | 23/04/2022 | 30/04/2022 | No | No | Mild | Unlikely |
| C3 | Female | 23/03/2022 | Catheter site pain | 24/03/2022 | 29/03/2022 | No | No | Mild | Possible |
| C3 | Female | 23/03/2022 | Dry mouth | 26/03/2022 | 28/03/2022 | No | No | Mild | Possible |
| C3 | Female | 23/03/2022 | Headache | 25/03/2022 | 25/03/2022 | No | No | Mild | Possible |
| C3 | Female | 23/03/2022 | Heavy menstrual bleeding | 09/04/2022 | 23/04/2022 | No | No | Moderate | Possible |
| C3 | Male | 23/03/2022 | Upper respiratory tract infection bacterial | 01/05/2022 | 07/05/2022 | No | No | Moderate | Unlikely |
| C3 | Male | 23/03/2022 | Viral upper respiratory tract infection | 23/03/2022 | 28/03/2022 | No | No | Mild | Possible |
| C4 | Male | 06/04/2022 | Abscess | 06/04/2022 | 09/04/2022 | No | No | Mild | No relationship |
| C4 | Female | 06/04/2022 | Breast abscess | 05/07/2022 | 19/07/2022 | No | No | Moderate | Unlikely |
| C4 | Female | 06/04/2022 | Gastroenteritis viral | 08/04/2022 | 08/04/2022 | No | No | Mild | Possible |
| C4 | Female | 06/04/2022 | Headache | 16/05/2022 | 18/05/2022 | No | No | Mild | Unlikely |
| C4 | Female | 06/04/2022 | Panic attack | 06/04/2022 | 06/04/2022 | No | No | Mild | Possible |
| CM1 | Female | 19/01/2023 | Aphthous ulcer | 28/01/2023 | 02/03/2023 | No | No | Moderate | Possible |
| CM1 | Female | 19/01/2023 | Headache | 21/01/2023 | 24/01/2023 | No | No | Mild | Unlikely |
| CM1 | Female | 19/01/2023 | Headache | 27/01/2023 | 02/03/2023 | No | No | Moderate | Unlikely |
| CM2 | Male | 02/02/2023 | Abscess | 02/03/2023 | 16/03/2023 | No | No | Severe | No relationship |
| CIV1 | Female | 16/03/2022 | Gastroenteritis viral | 17/03/2022 | 17/03/2022 | No | No | Mild | Possible |
| CIV1 | Male | 16/03/2022 | Gastroenteritis viral | 18/03/2022 | 19/03/2022 | No | No | Mild | Possible |
| CIV1 | Female | 16/03/2022 | Gastroenteritis viral | 18/03/2022 | 24/03/2022 | No | No | Mild | Possible |
| CIV1 | Male | 16/03/2022 | Headache | 03/04/2022 | 04/04/2022 | No | No | Mild | Unlikely |

## Line listing of laboratory adverse events

| **Cohort** | **Gender** | **Date of study drug administration** | **Laboratory adverse event** | **Laboratory value** | **Date of abnormal result** | **Serious** | **Severity** |
| --- | --- | --- | --- | --- | --- | --- | --- |
| C1 | Male | 02/03/2022 | Leukopaenia | 3 | 07/03/2022 | No | Mild |
| C1 | Male | 02/03/2022 | Leukopaenia | 3 | 14/04/2022 | No | Mild |
| C1 | Male | 02/03/2022 | Low haemoglobin | 11.4 | 07/03/2022 | No | Mild |
| C1 | Male | 02/03/2022 | Lymphopaenia | 1.1 | 07/03/2022 | No | Mild |
| C1 | Male | 02/03/2022 | Lymphopaenia | 1.19 | 14/04/2022 | No | Mild |
| C1 | Male | 02/03/2022 | Raised creatinine | 149 | 07/03/2022 | No | Mild |
| C1 | Male | 02/03/2022 | Thrombocytopaenia | 97 | 04/03/2022 | No | Moderate |
| C2 | Male | 09/03/2022 | Hypernatraemia | 145 | 14/03/2022 | No | Mild |
| C2 | Male | 09/03/2022 | Hypernatraemia | 146 | 20/04/2022 | No | Mild |
| C2 | Male | 09/03/2022 | Neutropaenia | 0.77 | 20/04/2022 | No | Moderate |
| C2 | Male | 09/03/2022 | Raised ALT | 64 | 10/03/2022 | No | Mild |
| C2 | Male | 09/03/2022 | Raised ALT | 73 | 11/03/2022 | No | Mild |
| C2 | Male | 09/03/2022 | Raised ALT | 61 | 11/03/2022 | No | Mild |
| C2 | Male | 08/03/2022 | Raised ALT | 59 | 14/03/2022 | No | Mild |
| C2 | Male | 09/03/2022 | Raised ALT | 64 | 14/03/2022 | No | Mild |
| C2 | Male | 09/03/2022 | Raised ALT | 159 | 17/03/2022 | No | Moderate |
| C3 | Male | 23/03/2022 | Hypernatraemia | 147 | 24/03/2022 | No | Mild |
| C3 | Male | 23/03/2022 | Hypernatraemia | 147 | 24/03/2022 | No | Mild |
| C3 | Female | 23/03/2022 | Hypernatraemia | 153 | 24/03/2022 | No | Moderate |
| C3 | Female | 16/03/2022 | Neutropaenia | 1.03 | 27/04/2022 | No | Mild |
| C3 | Female | 16/03/2022 | Raised creatinine | 103 | 21/03/2022 | No | Mild |
| C4 | Female | 06/04/2022 | Hypernatraemia | 146 | 07/04/2022 | No | Mild |
| C4 | Female | 06/04/2022 | Low haemoglobin | 9.9 | 11/04/2022 | No | Mild |
| CM1 | Male | 19/01/2023 | Leukopaenia | 3.1 | 21/01/2023 | No | Mild |
| CM1 | Female | 19/01/2023 | Low haemoglobin | 9.8 | 02/03/2023 | No | Mild |
| CM1 | Male | 19/01/2023 | Low haemoglobin | 11.3 | 21/01/2023 | No | Mild |
| CM1 | Female | 19/01/2023 | Low haemoglobin | 10 | 24/01/2023 | No | Mild |
| CM1 | Male | 19/01/2023 | Low haemoglobin | 10.8 | 24/01/2023 | No | Mild |
| CM1 | Male | 19/01/2023 | Lymphopaenia | 1.14 | 21/01/2023 | No | Mild |
| CM1 | Male | 19/01/2023 | Lymphopaenia | 1.18 | 24/01/2023 | No | Mild |
| CM2 | Male | 02/02/2023 | Leukopaenia | 3.2 | 07/02/2023 | No | Mild |
| CM2 | Male | 02/02/2023 | Leukopaenia | 2.9 | 16/03/2023 | No | Mild |
| CM2 | Male | 02/02/2023 | Leukopaenia | 3 | 16/03/2023 | No | Mild |
| CM2 | Male | 02/02/2023 | Low haemoglobin | 11.3 | 05/02/2023 | No | Mild |
| CM2 | Male | 02/02/2023 | Low haemoglobin | 10.7 | 07/02/2023 | No | Mild |
| CM2 | Male | 02/02/2023 | Lymphopaenia | 1.15 | 03/02/2023 | No | Mild |
| CM2 | Female | 02/02/2023 | Lymphopaenia | 1.39 | 03/02/2023 | No | Mild |
| CM2 | Male | 02/02/2023 | Lymphopaenia | 1.13 | 16/03/2023 | No | Mild |
| CIV1 | Male | 16/03/2022 | Leukopaenia | 3.2 | 21/03/2022 | No | Mild |
| CIV1 | Male | 16/03/2022 | Lymphopaenia | 1.09 | 27/04/2022 | No | Mild |
| CIV1 | Male | 16/03/2022 | Neutropaenia | 1.05 | 27/04/2022 | No | Mild |
| CIV1 | Male | 16/03/2022 | Raised ALT | 114 | 17/03/2022 | No | Moderate |
| CIV1 | Male | 16/03/2022 | Raised ALT | 96 | 18/03/2022 | No | Moderate |
| CIV2 | Male | 24/01/2023 | Hyperkalaemia | 5.5 | 07/03/2023 | No | Mild |
| CIV2 | Male | 24/01/2023 | Leukopaenia | 2.8 | 07/03/2023 | No | Mild |
| CIV2 | Male | 24/01/2023 | Leukopaenia | 3.1 | 25/01/2023 | No | Mild |
| CIV2 | Male | 24/01/2023 | Leukopaenia | 3.2 | 26/01/2023 | No | Mild |
| CIV2 | Male | 24/01/2023 | Lymphopaenia | 0.89 | 25/01/2023 | No | Mild |
| CIV2 | Male | 24/01/2023 | Lymphopaenia | 1.13 | 26/01/2023 | No | Mild |
| CIV2 | Male | 24/01/2023 | Lymphopaenia | 1.17 | 29/01/2023 | No | Mild |
| CIV2 | Male | 24/01/2023 | Raised ALT | 65 | 26/01/2023 | No | Mild |
| CIV2 | Male | 24/01/2023 | Raised ALT | 122 | 29/01/2023 | No | Moderate |
| CIV2 | Male | 24/01/2023 | Thrombocytopaenia | 105 | 25/01/2023 | No | Mild |
| CIV2 | Male | 24/01/2023 | Thrombocytopaenia | 112 | 26/01/2023 | No | Mild |
| CIV2 | Male | 24/01/2023 | Thrombocytopaenia | 121 | 29/01/2023 | No | Mild |

## Plots of alanine aminotransferase, total white cell count, and serum creatinine time course amongst participants with an abnormal result

Temporal trends of ALT, WCC, creatinine values for participants with a value above the upper limit of normal at any time after receiving the study drug through to the time of their final blood test. The timepoints are as follows: pre-screen (the day before study drug administration), dosing day, day-1, day-2, day-5, day-42, and any unscheduled blood tests throughout the follow-up period. Each line represents an individual participant, and these are grouped by colour according to dosing cohort. C1: 300 mg single oral; C2: 900 mg single oral; C3: 1,200 mg single oral; C4: 1,500 mg single oral; CM1: 0-hour/1,500 mg oral, 6-hour/900 mg oral, 24-hour/900 mg oral; CM2: 0-hour/1,500 mg oral, 6-hour/1,500 mg oral, 24-hour/1,500 mg oral; CIV1: 3 mg/kg single intravenous; CIV2: 5 mg/kg single intravenous

## Overview of pre-dose and post-dose QTc-interval data by dosing cohort

|  | **Pre-dose^a^** | **Post-dose^b^** |
| --- | --- | --- |
| **Cohort** | **Fridericia QTc mean (SD); range** | **Fridericia QTc mean (SD); range** |
| C1 | 385.8 (26.7); 354-425 | 381 (31.9); 349-440 |
| C2 | 388.5 (25); 343-412 | 390.5 (32.8); 353-457 |
| C3 | 391.2 (30.4); 340-447 | 388 (35.2); 308-423 |
| C4 | 372 (20.8); 344-400 | 380.1 (22); 348-417 |
| CIV1 | 391.9 (34.6); 331-439 | 380.5 (29.2); 351-430 |
| CIV2 | 371 (24.9); 332-418 | 373.2 (25); 338-431 |
| CM1 | 396.3 (37.5); 331-450 | 397.8 (32.4); 324-464 |
| CM2 | 377.7 (25.6); 344-425 | 361.5 (19.8); 341-412 |

a – the mean, standard deviation and range of QTc intervals has been presented for electrocardiograms recorded prior to administration of unithiol, by dosing cohort.

b - The mean, standard deviation and range of QTc intervals has been presented for electrocardiograms recorded within the first 24-hours after administration of unithiol, by dosing cohort.

C1: 300 mg single oral; C2: 900 mg single oral; C3: 1,200 mg single oral; C4: 1,500 mg single oral; CM1: 0-hour/1,500 mg oral, 6-hour/900 mg oral, 24-hour/900 mg oral; CM2: 0-hour/1,500 mg oral, 6-hour/1,500 mg oral, 24-hour/1,500 mg oral; CIV1: 3 mg/kg single intravenous; CIV2: 5 mg/kg single intravenous; SD: standard deviation.

## Baseline characteristics of the PK population

| **Dose** | **Number** | **Male (n, %)** | **Age, years (median, IQR)** | **Weight, kg (median, IQR)** | **Creatinine, µmol/L (median, IQR)** |
| --- | --- | --- | --- | --- | --- |
| 300 mg oral | 4 | 3 (75 %) | 46 (IQR 34-53) | 59 (IQR 58-70) | 87 (IQR 86-88) |
| 900 mg oral | 4 | 4 (100 %) | 30 (IQR 26-36) | 58 (IQR 57-60) | 96 (IQR 87-104) |
| 1,200 mg oral | 4 | 3 (75 %) | 28 (IQR 26-31) | 57 (IQR 56-60) | 88 (IQR 86-94) |
| 1,500 mg oral | 4 | 3 (75 %) | 29 (IQR 26-33) | 61 (IQR 60-63) | 96 (IQR 86-103) |
| 3 mg/kg intravenous | 4 | 4 (100 %) | 24 (IQR 22-26) | 64 (IQR 62-67) | 100 (IQR 98-104) |
| 5 mg/kg intravenous | 8 | 7 (88 %) | 36 (IQR 29-43) | 62 (IQR 59-64) | 102 (IQR 95-106) |
| 0-hour: 1,500 mg oral  6-hour: 900 mg oral  24-hour: 900 mg oral | 8 | 7 (88 %) | 26 (IQR 22-36) | 55 (IQR 53-57) | 90 (IQR 85-98) |
| 0-hour: 1,500 mg oral  6-hour: 1,500 mg oral  24-hour: 1,500 mg oral | 8 | 7 (88 %) | 25 (IQR 24-34) | 58 (IQR 54-62) | 95 (IQR 95-96) |
| **All** | **44** | **55 (86%)** | **28 (IQR 25-39)** | **59 (IQR 56-63)** | **95 (IQR 87-102)** |

IQR: interquartile range; kg: kilograms.

## Dose normalised plasma concentrations of unithiol

**Single oral ascending doses**

**Single intravenous ascending doses**

Dose normalised median concentrations of unithiol over time for participants that received single doses of unithiol (C1: 300 mg single oral; C2: 900 mg single oral; C3: 1,200 mg single oral; C4: 1,500 mg single oral; CIV1: 3 mg/kg single intravenous; CIV2: 5 mg/kg single intravenous). The plasma concentrations were normalised by dividing by the proportional difference in dose from 300 mg for the oral doses, and from 3 mg/kg for the intravenous doses (i.e., the plasma concentrations for the 1,500 mg dose group were divided by 1,500/300 ≡ 5; and the 5 mg/kg intravenous group by 5/3 ≡ 1.66. The error bars represent the interquartile range at each time point.

## Dose normalised unithiol pharmacokinetic parameters in the PK population

**Single oral ascending doses**

| **Cohort** | **Dose normalised C_max_ (µg/mL/mg)** | **Dose normalised AUC_0-∞_ (µg.h/mL/mg)** |
| --- | --- | --- |
| C1 (n=4) | 0.014 (0.010-0.021) | 0.147 (0.078–0.277) |
| C2 (n=4) | 0.011 (0.009–0.014) | 0.154 (0.099–0.238) |
| C3 (n=4) | 0.011 (0.006–0.022) | 0.143 (0.082–0.250) |
| C4 (n=4) | 0.010 (0.005–0.019) | 0.136 (0.093–0.199) |

**Single intravenous ascending doses**

| **Cohort** | **Dose normalised C_5-min_ (µg/mL/mg)** | **Dose normalised AUC_0-∞_ (µg.h/mL/mg)** |
| --- | --- | --- |
| CIV1 (n=4) | 0.071 (0.033–0.155) | 0.330 (0.231–0.472) |
| CIV2 (n=8) | 0.130 (0.100–0.170) | 0.455 (0.370–0.560) |

Data are geometric mean, with the 95% confidence intervals for each parameter in parentheses. Dose normalisation was calculated by dividing the geometric mean C_max_, C_5-min_, AUC_0-∞_, and the corresponding 95% confidence intervals by the dose administered, in mg units (i.e., for the 300 mg cohort, these parameters were divided by 300). For the intravenous cohorts, which received weight-based doses, the median actual dose administered was used as the denominator (this was 193.1 mg for the CIV1 cohort and 301.0 mg for the CIV2 cohort).

C1: 300 mg single oral; C2: 900 mg single oral; C3: 1,200 mg single oral; C4: 1,500 mg single oral; CIV1: 3 mg/kg single intravenous; CIV2: 5 mg/kg single intravenous; C_5-min_: plasma concentration at 5-minutes after the initiation of the intravenous infusion; C_max_: maximum plasma concentration; T_max_: time to reach C_max_; T_1/2_: half-life; AUC_0-∞_: area under the curve from time zero extrapolated to infinity.

## Estimated oral bioavailability of unithiol

| **Cohort** | **Route of administration** | **Median dose (mg)** | **Median AUC_0-∞_ (µg.h/mL)** | **Oral bioavailability compared with CIV1 cohort (%)** | **Oral bioavailability compared with CIV2 cohort (%)** |
| --- | --- | --- | --- | --- | --- |
| C1 | Oral | 300 | 49.8 | 47.1 | 60.7 |
| C2 | Oral | 900 | 147.1 | 46.4 | 59.7 |
| C3 | Oral | 1,200 | 191.5 | 45.3 | 58.3 |
| C4 | Oral | 1,500 | 203.5 | 38.5 | 49.6 |
| CIV1 | Intravenous | 190 | 66.9 | - | - |
| CIV2 | Intravenous | 300 | 82.1 | - | - |

Oral bioavailabilty was estimated using the following formula: (median oral AUC_0-∞_ /median intravenous AUC_0-∞_) x (median intravenous dose/median oral dose) x 100

AUC_0-∞_: area under the curve from time zero extrapolated to infinity; C1: 300 mg single oral; C2: 900 mg single oral; C3: 1,200 mg single oral; C4: 1,500 mg single oral; CM1: 0-hour/1,500 mg oral, 6-hour/900 mg oral, 24-hour/900 mg oral; CM2: 0-hour/1,500 mg oral, 6-hour/1,500 mg oral, 24-hour/1,500 mg oral; CIV1: 3 mg/kg single intravenous; CIV2: 5 mg/kg single intravenous.

## CONSORT Dose-finding Extension (CONSORT-DEFINE) guidance

| **Category and section** | **Standard CONSORT 2010**  **checklist item** | | **CONSORT-DEFINE checklist item**  **for EPDF Trials** | | **Addressed on Page No^¤^** |
| --- | --- | --- | --- | --- | --- |
|  | **Item No** | **CONSORT 2010** | **Item**  **No** | **CONSORT-DEFINE** |  |
| **Title and abstract** | | | | | |
|  | 1a | Identification as a randomised trial in the title | 1a† | Identification as an early phase dose-finding (eg, first-in-human, dose escalation or de-escalation, phase 1, phase 1/2, expansion, dose titration) and, if applicable, randomised trial in the title or abstract | 1-2 |
|  | 1b | Structured summary of trial design, methods, results, and conclusions (for specific guidance, see CONSORT for abstracts) | 1b | Structured summary of trial design, methods, results, and conclusions (for specific guidance, see CONSORT-DEFINE for abstracts) | 2 |
| **Introduction** | | | | | |
| Background  and objectives | 2a | Scientific background and explanation of rationale | 2a.1† | Description of research question(s) and justification for undertaking the trial, including summary of relevant clinical studies (published and unpublished) examining benefits and harms for each intervention | 5-6 |
|  |  |  | 2a.2* | Summary of key findings from relevant non-clinical or preclinical research | 5 |
|  |  |  | 2a.3* | Summary of findings from previously generated preclinical and translational studies to support any planned biomarker substudies (where applicable) | NA |
|  | 2b | Specific objectives or hypotheses | 2b† | Specific objectives (eg, relating to safety, activity, pharmacokinetics, pharmacodynamics, recommended dose(s)) | 6 |
| **Methods** | | | | | |
| Trial design | 3a | Description of trial design (such as parallel, factorial) including allocation ratio | 3a.1† | Description of trial design elements, such as dose escalation or de-escalation strategy, number of treatment groups, allocation ratio if relevant, and details of any prespecified trial adaptations | 7-8 |
|  |  |  | 3a.2* | Trial design schema to show the flow of major transition points (eg, dose escalation to dose expansion, phase 1 to phase 2, single ascending dose to multiple ascending dose) | Table 1  Figure 1 |
|  |  |  | 3a.3* | Statistical methods or rationale underpinning the trial design | 9-10 |
|  |  |  | 3a.4* | Starting dose(s) with rationale | 8 |
|  |  |  | 3a.5* | Range of planned dose levels with rationale | 8 |
|  |  |  | 3a.6* | Presentation of planned dose levels (eg, as a diagram, table, or infographic), where applicable | Table 1 |
|  |  |  | 3a.7* | Skipping of dose level(s), if applicable | NA |
|  |  |  | 3a.8* | Planned cohort size(s) (eg, fixed, flexible, adaptive) | 7-9 |
|  |  |  | 3a.9* | Dose allocation method within a dose level (including sequence and interval between dosing of participants, eg, sentinel or staggered dosing) | 7-8 |
|  |  |  | 3a.10* | Dose expansion cohort(s), if applicable, with rationale | NA |
|  |  |  | 3a.11* | Criteria for progression to the next part of the trial (eg, phase 1 to phase 2, single ascending dose to multiple ascending dose), where applicable | 6 |
|  | 3b | Important changes to methods after trial commencement (such as eligibility criteria), with reasons | 3b† | Important changes to the design or methods after trial commencement (eg, insertion of unplanned additional doses) outside the scope of the prespecified adaptive design features, with reasons | NA |
| Participants | 4a | Eligibility criteria for participants | 4a |  | 7 |
|  | 4b | Settings and locations where the data were collected | 4b |  | 6 |
| Interventions | 5 | The interventions for each group with sufficient details to allow replication, including how and when they were actually administered | 5a† | Interventions for each dose level (within each group) with sufficient details to allow replication, including administration route and schedule showing how and when they were actually administered | 7-8  Table 1 |
|  |  |  | 5b* | Criteria for dose discontinuation, dose modifications, and dosing delays of allocated interventions for a given trial participant (eg, dose change in response to harms, participant request, or improving or worsening disease) | NA |
| Outcomes | 6a | Completely defined prespecified primary and secondary outcome measures, including how and when they were assessed | 6a† | Primary and secondary outcomes, including the specific measurement variable, analysis metric, method of aggregation, and time point for each outcome. Explanation of the clinical relevance of chosen outcomes is strongly recommended. Any other outcomes used to inform prespecified adaptations should be described with the rationale | 6 |
|  | 6b | Any changes to trial outcomes after the trial commenced, with reasons | 6b† | Any unplanned changes to trial outcomes after the trial commenced, with reasons | 17 |
| Sample size | 7a | How sample size was determined | 7a† | Estimated number of participants (minimum, maximum, or expected range) needed to address trial objectives and how it was determined, including clinical and statistical assumptions supporting any sample size and operating characteristics | 9 |
|  | 7b | When applicable, explanation of any interim analyses and stopping guidelines | 7b† | Prespecified interim decision making criteria or rules that guided the trial adaptation process (eg, dosing decision to (de-)escalate); prespecified and actual timing and frequency of interim data reviews and the information to inform trial adaptations | 6 |
| Randomization (if applicable) | | | | | |
| Sequence generation | 8a | Method used to generate the random allocation sequence | 8a |  | 7 |
|  | 8b | Type of randomisation; details of any restriction (such as blocking and block size) | 8b† | Type of randomisation; details of any restrictions (such as blocking and block size); any prespecified adaptive assignment rules or algorithm leading to adjustments in the allocation ratio, including timing and frequency of updates; any changes to the allocation rule following trial adaptation decisions | 7 |
| Allocation concealment mechanism | 9 | Mechanism used to implement the random allocation sequence (such as sequentially numbered containers), describing any steps taken to conceal the sequence until interventions were assigned | 9 |  | 7 |
| Implementation | 10 | Who generated the random allocation sequence, who enrolled participants, and who assigned participants to interventions | 10 |  | 7 |
| Blinding | 11a | If done, who was blinded after assignment to interventions (eg, participants, care providers, and how | 11a |  | NA |
|  | 11b | If relevant, description of the similarity of interventions | 11b |  | NA |
| Statistical methods | 12a | Statistical methods used to compare groups for primary and secondary outcomes | 12a.1† | Statistical methods for primary and secondary outcomes and any other outcomes used to make prespecified adaptations | 9-10 |
|  |  |  | 12a.2* | For the implemented adaptive design features, statistical methods used for estimation (eg, safety, dose(s), treatment effects) and to make inferences | NA |
|  | 12b | Methods for additional analyses, such as subgroup analyses and adjusted analyses | 12b† | Statistical methods for additional analyses (eg, subgroup and adjusted analyses, pharmacokinetics or pharmacodynamics, biomarker correlative analyses) | 9-10 |
|  |  |  | 12c* | Analysis population(s) (eg, evaluable population for dose-finding, safety population) | 10 + 13 |
|  |  |  | 12d* | Strategies for handling intercurrent events occurring after treatment initiation (eg, how dosing adjustments were handled) that can affect either the interpretation or the existence of the measurements associated with the clinical question of interest, and any methods to handle missing data | NA |
| **Results** | | | | | |
| Participant flow (a diagram is strongly recommended) | 13a | For each group, the numbers of participants who were randomly assigned, received intended treatment, and were analysed for the primary outcome | 13a† | For each group, the number of participants who were assigned to each dose level at each interim analysis (eg, for dosing decisions), received intended treatment, and were analysed for the primary outcome and, if applicable, any other outcomes used to inform prespecified adaptations | Figure 1 |
|  | 13b | For each group, losses and exclusions after randomisation, together with reasons | 13b† | For each group, losses and exclusions after allocation to each dose level, together with reasons | Figure 1 |
| Recruitment | 14a§ | Dates defining the periods of recruitment and follow-up | 14a§ |  | 8 + 10 |
|  | 14b§ | Why the trial ended or was stopped | 14b§ |  | NA |
|  |  |  | 14c* | Trial adaptation decisions made (including on what basis they were made, and when) in light of the prespecified decision making criteria and observed accrued data | 12 |
| Baseline data | 15 | A table showing baseline demographic and clinical characteristics for each group | 15† | Baseline demographic and clinical characteristics across each dose level within each group, where appropriate | Table 2 |
| Numbers analysed | 16 | For each group, number of participants (denominator) included in each analysis and whether the analysis was by original assigned groups | 16† | For each group, the number of participants (denominator) included in each analysis across each dose level, and whether the analysis was by original assigned interventions | Figure 1 |
| Outcomes and estimation | 17a | For each primary and secondary outcome, results for each group, and the estimated effect size and its precision (such as 95% confidence interval) | 17a† | For each primary and secondary outcome, results for each dose level within each group, and the estimated effect size and its precision, if applicable | 10-14 |
|  | 17b§ | For binary outcomes, presentation of both absolute and relative effect sizes is recommended | 17b§ |  | NA |
|  |  |  | 17c* | Report interim results used to inform interim decision making such as dose escalation, de-escalation, or staying at the same dose | 12 |
| Ancillary analyses | 18 | Results of any other analyses performed, including subgroup analyses and adjusted analyses, distinguishing prespecified from exploratory | 18 |  | NA |
| Harms | 19 | All important harms or unintended effects in each group (for specific guidance, see CONSORT for harms (1)) | 19† | All important harms (eg, adverse events or effects, toxicities) reported by dose level in each group (for specific guidance, see CONSORT for harms (2)) | Table 3 |
| **Discussion** | | | | | |
| Limitations | 20 | Trial limitations, addressing sources of potential bias, imprecision, and, if relevant, multiplicity of analyses | 20 |  | 17 |
| Generalizability | 21 | Generalisability (external validity, applicability) of the trial findings | 21 |  | 17 |
| Interpretation | 22 | Interpretation consistent with results, balancing benefits and harms, and considering other relevant evidence | 22 |  | 17-18 |
| **Other information** | | | | | |
| Registration | 23 | Registration number and name of trial registry | 23 |  | 9 |
| Protocol | 24 | Where the full trial protocol can be accessed, if available | 24 |  | 9 |
| Funding | 25 | Sources of funding and other support (such as supply of drugs), role of funders | 25 |  | 10 + 19 |
| Data monitoring |  |  | 26a* | Composition of any decision making or safety review committee or group; summary of its role and reporting structure; statement of whether it is independent from the sponsor and competing interests; and reference to where further details can be found (such as in a charter or protocol) | 19-20 |
|  |  |  | 26b* | Description of who had access to interim results and made the interim and final decision to terminate the trial (or part(s) of the trial, eg, end of dose escalation), and measures to safeguard the confidentiality of interim information | 12 |
| Dissemination |  |  | 27* | Specify, if applicable, whether and when results (such as safety and/or activity) were reported externally (eg, through scientific presentations, journal publication, or the trial website) while the trial (or part(s) of the trial) was still ongoing | NA |

CONSORT=CONsolidated Standards Of Reporting Trials; DEFINE=Dose-finding Extension; EPDF=early phase dose-finding.
