# Supplementary material for: Development of an oral regimen of unithiol for the treatment of snakebite envenoming: a phase 1 open-label dose-escalation safety trial and pharmacokinetic analysis in healthy Kenyan adults
Source: eBioMedicine. 2025 Feb 27;113:105600. doi: 10.1016/j.ebiom.2025.105600 (PMC11919382; doi:10.1016/j.ebiom.2025.105600)
Supplement: TRUE1_published_protocol [file mmc2.pdf]

Check for updates

STUDY PROTOCOL

TRUE-1: Trial of Repurposed Unithiol for snakebite Envenoming phase 1 (safety, tolerability, pharmacokinetics and pharmacodynamics in healthy Kenyan adults) [version 1; peer review: 3 approved]

Michael Abouyannis[1,2], Richard FitzGerald[3], Mwanajuma Ngama[2], Hope Mwangudzah[2], Yvonne K. Nyambura[2], Samson Ngome[2], Debra Riako[2], Lawrence Babu[2], Frida Lewa[2], Laura Else[4], Sujan Dily Penchala[4], Benedict Orindi[2], Noni Mumba[2], Betty Kalama[2], Francis M. Ndungu[2], Ifedayo Adetifa[2,5], Saye Khoo[3,4], David G. Lalloo[1], Nicholas R. Casewell[1,6], Mainga Hamaluba[2,7]

[1]Centre for Snakebite Research & Interventions, Liverpool School of Tropical Medicine, Liverpool, UK
[2]KEMRI-Wellcome Research Programme, Kilifi, Kenya
[3]NIHR Royal Liverpool and Broadgreen CRF, Liverpool University Hospitals NHS Foundation Trust, Liverpool, UK
[4]Department of Molecular and Clinical Pharmacology, University of Liverpool, Liverpool, UK
[5]Department of Infectious Diseases Epidemiology, London School of Hygiene & Tropical Medicine, London, UK
[6]Centre for Drugs & Diagnostics, Liverpool School of Tropical Medicine, Liverpool, UK
[7]Centre for Tropical Medicine & Global Health, Nuffield Department of Medicine, University of Oxford, Oxford, UK

v1 **First published:** 14 Mar 2022, **7**:90 https://doi.org/10.12688/wellcomeopenres.17682.1
Latest published: 14 Mar 2022, **7**:90 https://doi.org/10.12688/wellcomeopenres.17682.1

Abstract

Background: Snakebites affect over 5 million people each year, and over 100,000 per year die as a result. The only available treatment is antivenom, which has many shortcomings including high cost, intravenous administration, and high risk of adverse events. One of the most abundant and harmful components of viper venoms are the zinc-dependent snake venom metalloproteinases (SVMPs). Unithiol is a chelating agent which is routinely used to treat heavy metal poisoning. *In vivo* experiments in small animal models have demonstrated that unithiol can prevent local tissue damage and death caused by a certain viper species. This phase I clinical trial will assess the safety of ascending doses of unithiol with a view for repurposing for snakebite indication.

Methods: This open label, single agent, phase I clinical trial of a repurposed drug has a primary objective to evaluate the safety of escalating doses of unithiol, and a secondary objective to describe its pharmacokinetics. In total, 64 healthy Kenyan volunteers from Kilifi County will be dosed in consecutive groups of eight, with dose escalation decisions dependent on review of safety data by an

Open Peer Review

Approval Status ✓ ✓ ✓

	1	2	3
**version 1** 14 Mar 2022	✓ view	✓ view	✓ view

1. **Eric Lavonas**, Denver Health, Denver, USA
Denver Health, Denver, USA
University of Colorado School of Medicine, Aurora, USA

2. **Matthew Lewin**, California Academy of Sciences, San Francisco, USA

3. **Matthew Coldiron**, Epicentre - Médicins Sans Frontières, Paris, France

independent data safety monitoring board. Four groups will receive ascending single oral doses, two will receive multiple oral doses, and two will receive single intravenous doses. Follow-up will be for 6-months and includes full adverse event reporting. Pharmacokinetic analysis will define the Cmax, Tmax, half-life and renal elimination.
Conclusions: This clinical trial will assess the safety and tolerability of a promising oral therapeutic in a relevant setting where snakebites are prevalent. Unithiol is likely to be safer than antivenom, is easier to manufacture, has activity against diverse snake species, and can be administered orally, and thus shows promise for repurposing for tropical snakebite.
Pan African Clinical Trials Registry: PACTR202103718625048 (3/3/2021)

Keywords
Snakebite, envenoming, small molecule, chelator, phase I, adaptive, repurpose, clinical trial.

Any reports and responses or comments on the article can be found at the end of the article.

KEMRI wellcome

This article is included in the KEMRI | Wellcome Trust gateway.

Corresponding author: Michael Abouyannis (michael.abouyannis@lstmed.ac.uk)

Author roles: Abouyannis M: Conceptualization, Funding Acquisition, Investigation, Methodology, Project Administration, Writing – Original Draft Preparation; **FitzGerald R**: Conceptualization, Methodology, Supervision, Writing – Original Draft Preparation; **Ngama M**: Investigation, Project Administration; **Mwangudzah H**: Investigation, Project Administration; **Nyambura YK**: Investigation, Project Administration; **Ngome S**: Investigation, Project Administration; **Riako D**: Investigation, Project Administration; **Babu L**: Data Curation, Project Administration; **Lewa F**: Data Curation, Project Administration; **Else L**: Investigation, Methodology, Project Administration; **Dily Penchala S**: Investigation, Methodology, Project Administration; **Orindi B**: Data Curation, Methodology, Software; **Mumba N**: Project Administration; **Kalama B**: Project Administration; **Ndungu FM**: Methodology, Supervision, Writing – Review & Editing; **Adetifa I**: Methodology, Supervision, Writing – Review & Editing; **Khoo S**: Funding Acquisition, Methodology, Supervision, Writing – Review & Editing; **Lalloo DG**: Conceptualization, Funding Acquisition, Methodology, Supervision, Writing – Review & Editing; **Casewell NR**: Conceptualization, Funding Acquisition, Methodology, Project Administration, Supervision, Writing – Review & Editing; **Hamaluba M**: Conceptualization, Funding Acquisition, Methodology, Project Administration, Supervision, Writing – Original Draft Preparation

Competing interests: No competing interests were disclosed.

Grant information: This work was supported by Wellcome [109105, https://doi.org/10.35802/109105; a Wellcome Trust fellowship to Michael Abouyannis]. Co-funding for this trial was provided by a ReGRoW Repurposing Research Grant from Cures Within Reach (CWR), and Research England's Bloomsbury SET Impact Connector Programme.
The funders had no role in study design, data collection and analysis, decision to publish, or preparation of the manuscript.

Copyright: © 2022 Abouyannis M *et al*. This is an open access article distributed under the terms of the Creative Commons Attribution License, which permits unrestricted use, distribution, and reproduction in any medium, provided the original work is properly cited.

How to cite this article: Abouyannis M, FitzGerald R, Ngama M *et al.* **TRUE-1: Trial of Repurposed Unithiol for snakebite Envenoming phase 1 (safety, tolerability, pharmacokinetics and pharmacodynamics in healthy Kenyan adults) [version 1; peer review: 3 approved]** Wellcome Open Research 2022, **7**:90 https://doi.org/10.12688/wellcomeopenres.17682.1

First published: 14 Mar 2022, **7**:90 https://doi.org/10.12688/wellcomeopenres.17682.1

Abbreviations

20WBCT	20-minute whole-blood clotting time
ALT	Alanine aminotransferase
AUC	Area under the curve
CGMRC-C	Centre for Geographic Medicine Coast
Cmax	Maximum plasma concentration
CRF	Case Report Form
CSRI	The Centre for Snakebite Research & Interventions
DMPS	2,3-bis(sulfanyl)propane-1-sulfonic acid (unithiol)
EDTA	Ethylenediaminetetraacetic acid
FBC	Full blood count
GCP	Good clinical practice
GDPR	General data protection regulation
KEMRI	Kenya Medical Research Institute
KHDSS	The Kilifi Health and Demographic Surveillance System
LSTM	Liverpool School of Tropical Medicine
SERU	Scientific and Ethics Review Unit
SPIRIT	Standard Protocol Items: Recommendations for Interventional Trials
SVMP	Snake venom metalloproteinase toxins
Tmax	Time to maximum concentration

Introduction

The burden of snakebite envenoming

Globally, snakebite envenoming is estimated to affect 1.8–2.7 million people, with annual mortality predicted between 80,000 and 140,000[1,2]. Despite this high mortality rate, snakebite envenoming has attracted minimal research funding, and therapeutic options have changed little over the past 50 years[3,4].

Snake venom metalloproteinase toxins

Snake venom metalloproteinase toxins (SVMPs) are abundant in viperid snake venoms and have an important role in causing:

- Haemorrhage via hydrolysis of endothelial cell wall components including type IV collagen, hyaluronic acid, and proteoglycans.
- Consumption coagulopathy via the activation of blood clotting factors.
- Local tissue damage, which is a prominent feature of envenoming in sub-Saharan Africa.

Limitations of antivenom

The mainstay of snakebite treatment is antivenom, which consists of polyclonal antibodies purified from immunised mammals, such as horses. Antivenom efficacy is variable and high costs have impacted on availability, particularly in sub-Saharan Africa[5].

When good quality antivenom directed against the relevant biting species is available, it has been shown to effectively reverse the consumption coagulopathy induced by SVMPs. However, few clinical trials of antivenom in sub-Saharan Africa have been conducted, and none have compared antivenom to placebo[6–10]. In Nigeria, ‘‘EchiTAb Plus-ICP’’ (ET-Plus) equine antivenom restored the 20-minute whole-blood clotting time (20WBCT) in 83% of trial participants[6]. Despite access to effective antivenom, in Nigeria snakebites are estimated to contribute to 1,900 deaths and 2,400 amputations, annually[11]. Unfortunately, antivenom has limited efficacy for treating venom-induced local tissue damage, probably because the onset of local tissue damage occurs before most people can access treatment[12,13]. The costs, low availability, risk of anaphylaxis and cold chain requirements of antivenom have restricted its use to secondary care settings. The risk of allergic reaction varies significantly between antivenom products. In a clinical trial conducted in Sri Lanka, the adverse reaction rate was 75%, and 43% suffered a severe adverse reaction[14].

SVMP inhibitory action of metal chelators

In the 1950s, *in vitro* experiments found that incubating venom with ethylenediaminetetraacetic acid (EDTA) completely inactivated its proteolytic activity[15,16]. Rabbits injected with venom that had been pre-incubated with EDTA were found to develop less necrosis[17]. In 1978, the importance of zinc in the haemorrhagic and proteolytic activities of venom components was described, and it was found that removing zinc with a metal chelator (1,10-phenanthroline) inhibited these activities[18]. More recently, research in mice has shown that administering $CaNa_2EDTA$ inhibits haemorrhagic, proteolytic and dermonecrotic effects of *Bothrops asper* venom[19]. In mice administered with a 2.5x LD_{50} dose of *Echis ocellatus* venom pre-incubated with EDTA, lethality was abolished. Furthermore, EDTA outperformed heterologous antivenom in terms of mice survival and survival times[20]. *In vitro* and mouse model research has since investigated the efficacy of various metal chelators to inhibit the pathological activity of various saw-scaled viper (*Echis* spp.) venoms[21]. Of those tested, unithiol (also known as sodium D,L-2,3-dimercapto-1-propanefulfonic acid [DMPS], trade name Dimaval®) showed the most promise as it is already in routine clinical use, has activity against a range of snake species, and significantly reduces envenoming associated mortality *in vivo*[21]. As well as preventing mortality, unithiol was demonstrated to effectively inhibit the formation of local haemorrhagic lesion at the site of venom delivery[21]. When unithiol was co-administered with antivenom *in vivo*, there was an additive effect with the combination treatment reducing mortality more than either product alone[21].

Summary of existing safety data for unithiol

Safety data in humans is available for unithiol, as this drug is routinely used for the treatment of heavy metal poisoning. Unithiol is generally regarded as a safe and well tolerated therapy.

Skin reactions

Skin reactions are the most common side effect reported for unithiol. They are invariably of low severity and of minor and short

impact to the individual. The rash is reversible on stopping the medication. These reactions are usually allergic in nature and tend to increase in likelihood with subsequent doses of unithiol.

Very rarely, unithiol can cause Stevens-Johnson syndrome. There are two published cases of Stevens-Johnson syndrome occurring during treatment with unithiol[22,23]. Given that unithiol has been in clinical use for several decades and has been given to many thousands of people, the risk of this complication is regarded as extremely low. The report by Chisolm *et al.* does not provide any clinical details[22]. In the report by Van Der Linde *et al.*, a widespread rash associated with blistering of the oral mucosa occurred in an 11-year old boy treated for mercury poisoning, with oral unithiol[23]. No cutaneous epidermal detachment occurred, and the child made a complete recovery following cessation of unithiol. In these case reports, the skin reaction did not manifest until 8–14 days after commencing unithiol at a dose of 200 mg three times per day oral.

Skin reactions associated with parenteral use of unithiol have only been described at very high doses. Attempts at administering parenteral 100 mg/kg to humans were conducted in the Soviet Union in the 1960s[24]. The study by Sanotskiĭ (available in Russian only), is referred to by Aposhian's review[25], which describes necrosis and ulceration at the site of subcutaneous or intravenous injection following these doses, which are 20 times higher than the currently recommended intravenous dose. These adverse skin reactions are not described for lower doses of parenteral unithiol.

Allergic and other immune mediated reactions

Severe allergic reactions to unithiol are extremely rare. Two cases of anaphylaxis and 21 cases of 'hypersensitivity' are reported in the Periodic Safety Update Report. The Dimaval® solution for injection summary of product characteristics suggests caution in administering the drug to people with allergic asthma, due to a risk of provoking an exacerbation[26]. The Periodic Safety Update Report identifies 6 reports of 'asthma' in association with use of unithiol.

Cardiovascular effects

Rapid infusion of unithiol solution via the intravenous route has been associated with transient asymptomatic hypotension in a single published article. In a previous pharmacokinetic study of unithiol, two of five participants developed transient falls in systolic blood pressure, of 20 mmHg[27]. These episodes were not associated with any symptoms, any change in heart rate, or any fall in diastolic blood pressure. Unithiol is not cardiotoxic and is not associated with any cases of clinically significant cardiac arrhythmia.

Mineral deficiency

Although the mechanism of action of unithiol would suggest that mineral deficiency would be an expected side effect (such as magnesium or zinc deficiency), this is not borne-out in published or post-marketing data. Data from Periodic Safety Update Reports have identified only a single case defined as 'non-serious decreased electrolytes.' No published reports of clinically significant mineral deficiency were identified.

Renal effects

Unithiol is not nephrotoxic.

Liver effects

Unithiol is not hepatotoxic.

Effects of higher doses of unithiol

Animal toxicity data have suggested that high doses of unithiol can be tolerated. In rats, 150 mg/kg administered orally 5 times per week for 63-weeks was tolerated with no observable effects[28]. In beagles, 45 mg/kg administered orally for 6-months was tolerated with no observable effects[29]. When a dose of 75 mg/kg was administered intravenously twice daily to beagles, anaemia as well as reduced iron content in the liver and spleen was noted[29]. In rabbits receiving 500 mg/kg administered orally twice daily for 6–10 days, no clinical events or changes in blood parameters were noted[30].

Published reports have also described giving high doses of unithiol to humans. Very high doses of parenteral unithiol (100 mg/kg) were given to people in the report by Sanotskiĭ in the 1960s (available in Russian only)[24]. This study is referred to in Aposhian's review[25], which describes necrosis and ulceration at the site of subcutaneous or intravenous injection. No other adverse events were described, although it is unclear if formal follow-up and adverse event reporting were followed.

The Dimaval® product monograph includes a report of the accidental administration of 100 mg/kg parenterally[30]. Skin necrosis at the injection site occurred, but no other morbidity was highlighted[30].

In a study by Stantschew *et al.* in 1974 (article in German), 216 adult workers with chronic mercury exposure were administered unithiol 1,000 mg intramuscularly. No adverse events were reported[31].

In a case report by Heinrich-Ramm *et al.*, a 21-year male with arsenic over-dose was administered the following regimen of unithiol:

- 250 mg/hour intravenous for 1 day
- 125 mg/hour intravenously on day 2
- 62.5 mg/hour intravenously on days 3–5

The total dose of unithiol received was 15.2 grams. A mild increase in transaminases were reported, but the patient was otherwise well and made a good recovery from the arsenic poisoning[32]. It is uncertain whether the transaminase rise was due to arsenic poisoning or unithiol.

Summary of existing pharmacokinetic data for unithiol

In total, four studies on unithiol pharmacokinetics have been conducted by a research group at The University of Arizona. Key finding from these studies are summarised in Table 1.

Absorption

In volunteers given 3x 100 mg capsules of unithiol, the drug was detected in blood between 0.5 and 4 hours after ingestion. Maximum concentrations were seen at 4 hours[33].

In 4 male volunteers, oral bioavailability was 39% (range 19–62%)[27].

Metabolism

Unithiol is rapidly and extensively metabolised to disulphide forms, which are not thought to be effective chelators. The predominant urine metabolites of unithiol are as follows: cyclic polymeric unithiol disulphides (97%), unithiol-cysteine mixed disulphide (2.5%) and acyclic unithiol disulphide (0.5%)[34]. In volunteers receiving intravenous unithiol, only 12% of unaltered drug was detected in blood after 15 minutes[27].

Distribution

Disulphide metabolites of unithiol are confined to the plasma, suggesting they are plasma protein bound[33]. In a study of three healthy adults who received a single oral dose of unithiol, at 5-hours 62.5% of the total plasma unithiol was protein bound[34]. Most (84%) of the protein bound unithiol was in the form of unithiol-albumin complex, and this is thought to be via disulphide linkage. The remaining non-protein bound drug consisted of unithiol disulphides (36.6%) and unaltered unithiol (0.9%). Amongst five healthy volunteers given intravenous unithiol, the volume of distribution varied from 2.7 to 15.4 L/kg[27].

Elimination

Unithiol is subject to renal elimination. In volunteers given intravenous unithiol (3 mg/kg), the elimination half-life was 1.8 hours. Of the total unithiol dose, 10% is found in the urine in an un-altered form, whereas 74% is excreted as disulphide metabolites[27].

Justification for this clinical trial

There is an urgent need to develop improved therapeutics for the treatment of snakebite. Every year, over 100,000 people die from snake envenoming, and the burden is particularly high in sub-Saharan Africa and Asia. The World Health Organization has targeted a 50% reduction in morbidity and mortality by 2030, and this trial could be a key step toward achieving this ambitious goal. Unithiol shows promise as a venom inhibitor based on robust pre-clinical measures of efficacy. The advantages of unithiol over antivenom are that it is safe, orally available, demonstrates cross-species specificity and is cheaper to produce.

Although unithiol has been safely administered to people for many decades, this will be the first study to explore its use as a therapeutic for snakebite. Unlike in heavy metal poisoning, the aim will be to rapidly achieve higher plasma levels, yet it will be possible to stop treatment after only 2 or 3 days. Limited pharmacokinetic data exists for unithiol, and this will be the first trial to investigate the pharmacokinetic effects of multiple doses. This will also be the first trial to evaluate high oral doses, as all previous 'high dose' studies have relied on parenteral administration. Oral administration will be important for using this drug in rural tropical settings. As snakebite tends to occur in rural settings, most victims initially present to a rural clinic where an early oral dose of unithiol could be given prior to transfer. Intravenous administration will be important for

Table 1. Summary of published human pharmacokinetic data.

Paper	Participants	Dose of unithiol	Main findings
Maiorino 1991[33]	10 male volunteers, aged 24”34 years, weighing 68”98 kg	300mg oral single dose	Absorbed unithiol is rapidly metabolised to disulphide forms. Cmax 11.9 μM, Tmax 3.7 hours, AUC 148 μM hours, half-life 9.1 hours (For total unithiol).
Hurlbut 1994[27]	5 volunteers (4 male, 1 female), aged 24 to 32 years, weighing 49”93 kg	3 mg/kg intravenous single dose	Unithiol is rapidly transformed to disulphide forms (>80% within 15 minutes). Excretion is mostly in urine in disulphide form. Elimination half-life 1.8 hours for parent drug; 20 hours for total unithiol.
Maiorino 1996[34]	4 male volunteers, aged 23 to 27 years, weighing 86”91 kg	300mg oral single dose	Majority of circulating unithiol (>60%) is plasma protein bound. Remaining drug is predominantly in disulphide form (>30%); remainder is unbound parent drug (<1%). Binds to albumin via disulphide complex. Majority of excreted drug is altered and in a disulphide form. Protein bound unithiol theorised to act as a reservoir of the drug and may prolong its activity.
Maiorino 1996[35]	11 factory workers with occupational mercury exposure (7 male and 4 female), mean age 34 years.	300 mg oral single dose	Identified increased mercury excretion and propose using unithiol as a challenge test for identifying mercury poisoning – this approach has since been discredited. Not relevant to present study.

AUC, area under the curve; Cmax, maximum plasma concentration; Tmax, time to maximum concentration.

those cases that present directly to a hospital facility with the capabilities of giving intravenous medications. Considering the dose-responsiveness of unithiol *in vitro*, as well as the need to rapidly achieve therapeutic levels, intravenous administration may be more efficacious.

Protocol

Approvals and trial registration

This trial was registered on the Pan African Clinical Trials Registry on the 3rd of March 2021 (PACTR202103718625048).

This manuscript corresponds to the approved version 2 protocol of the TRUE-1 trial, dated 5th of November 2020. This was approved by the Kenya Medical Research Institute Scientific and Ethics Review Unit on the 13th of January 2021 and by the Liverpool School of Tropical Medicine Research Ethics Committee on the 18th of February 2021. Regulatory approval for this protocol was provided by the Kenya Ministry of Health Pharmacy and Poisons Board on the 7th of August 2021.

This protocol has been prepared in accordance with Standard Protocol Items: Recommendations for Interventional Trials (SPIRIT) guidelines.

Trial design

This phase 1 open label trial of unithiol in healthy Kenyan adults has a primary outcome of determining safety and tolerability of unithiol. Single ascending doses of oral unithiol will be administered with dose escalation decisions dependent on emerging safety data. Two doses of intravenously administered unithiol will also be administered. Following an interim pharmacokinetic analysis and review of safety data from the single dosing stage, the trial will proceed to a multiple ascending dose stage. Participants will be followed-up for a period of 6-months with full reporting of adverse events and serious adverse events. Safety data will be reviewed by the data safety and monitoring board (DSMB) prior to dose escalations.

Trial objectives

Primary objective: To determine safety and tolerability of unithiol in healthy Kenyan adults.

Secondary objective: To define the pharmacokinetic profile of unithiol in healthy Kenyan adults.

Exploratory objective: To explore *ex vivo* efficacy of unithiol at varying doses to inhibit the pathogenic activity of *Echis ocellatus* and other venoms that rely on SVMPs

Study site

This single site study will be conducted in Kilifi County, Kenya. Participants will be recruited from Ngerenya, Chasimba and Kilifi township and will reside within the Kilifi Health and Demographic Surveillance System (KHDSS)[36]. Screening, dosing, and follow-up visits will be conducted at a clinical trial facility at Pwani University, Kilifi.

Recruitment

Members of the KHDSS will be approached using established recruitment strategies such as barazas, health talks, mobilization of community health volunteers and fieldworkers. Information giving sessions will be conducted by appropriately trained study personnel. Individuals who are interested in taking part will have ample time to read the patient information sheet. Contact details will be collected if available in accordance with our data collection policies. Those interested in the trial will be given written patient information in the appropriate language and invited to KEMRI-CGMRC or an appropriate facility for further information giving and informed consent.

The aim will be to enrol a diverse population from the KHDSS, including rural areas and the Kilifi township. Eligible consenting subjects will be sequentially screened until the required numbers are met. Ideally, equal numbers of male and female subjects will be enrolled (i.e., there will be a cap for each gender) to maintain a representative sample. Female subjects will be asked to demonstrate they are taking appropriate contraception from the screening visit (or the date of access to contraception for participants agreeing to referral to the health facility for this purpose) until 2 months after receiving the study drug (equivalent to two menstrual cycles).

Subjects will be allocated to a dosing cohort using stratified permuted block randomization. Stratification will be according to sex and age.

Study population

Healthy Kenyan adults will be invited to volunteer for this phase I clinical trial. The study team will aim to enrol people representative of a rural Kenyan population, within the constraints of the inclusion/exclusion criteria. There will be no restrictions based on income, education, and no requirement to speak English language. Ideally, there will be equal numbers of male and female subjects within each cohort.

Inclusion criteria

- Capable of giving informed consent
- Male or female
- KHDSS resident
- 18–64 years old (inclusive)
- Body weight 50–120kg
- In good health, as determined by the investigator following medical history, drug history, examination, vital signs, electrocardiogram (ECG), and blood tests
- Willing to be admitted to the in-patient facility for up to 5 days for dosing and intensive blood sampling
- On effective contraception as defined by: Use of effective method of contraception for duration of study

(women only). We will ask the female volunteers to come with their family planning records to verify. Effective contraception is defined as a contraceptive method with failure rate of less than 1% per year when used consistently and correctly, in accordance with the product label. Examples of these include: combined oral contraceptives; injectable progestogen; implants of etenogestrel or levonorgestrel; intrauterine device or intrauterine system; male partner sterilisation at least 6 months prior to the female subject's entry into the study, and the relationship is monogamous; male condom combined with a vaginal spermicide (foam, gel, film, cream or suppository); and male condom combined with a female diaphragm, either with or without a vaginal spermicide (foam, gel, film, cream, or suppository).

Exclusion criteria

- Prescribed a concomitant medication other than paracetamol or an appropriate contraceptive, which in the opinion of the Investigator warrants exclusion.
- Any significant current or history of cardiovascular, respiratory, renal, or hepatic disease.
- Subjects who have taken any non-prescribed herbal medication or mineral supplement in the preceding 7 days, which in the opinion of the Investigator warrants exclusion.
- Subject with clinically significant abnormal vital signs at screening
- Abnormal laboratory findings deemed significant by the Investigator
- Subjects who are pregnant or lactating
- Decline pre-trial screening, including human immunodeficiency virus (HIV) testing
- HIV positive subjects will be excluded from the trial and, if not already receiving appropriate clinic follow-up, would be referred to a government clinic for ongoing care
- Subjects with asthma (due to possible risk of exacerbation with allergic type skin reactions to unithiol)
- Subjects that have donated blood within the past 3 months
- Subjects who in the opinion of the investigator should not participate

Pre-dose exclusion criteria

Following pre-dose assessments, subjects may be excluded from the dosing cohort for the following reasons. This exclusion may be temporary, and the subject could join a later dosing cohort if the Investigator identifies an abnormality that is likely to resolve within an acceptable period, such as an acute infection.

- Clinically significant vital signs or 12 lead ECG findings
- Clinically significant abnormal laboratory findings
- Intercurrent illness
- Deviation from study restrictions (unless in the opinion of the Investigator these deviations will not interfere with the study procedures, compromise safety, or affect the study results. Any such deviations will be recorded in the source data and documented in the trial master file (TMF).

Screening

Subjects will be consented and then screened during a 3-month period prior to being dosed. Participants that are not dosed within 1-month of being screened will be re-screened. It is anticipated that screening will be held once before the single dosing stage and secondly prior to the multiple dosing stage. Additional screening dates may be needed depending on the time taken to dose escalate. The screening visit will be conducted by a clinician at KEMRI-CGMRC or an appropriate facility. The patient information sheet will be given, and the information will be provided verbally and understanding confirmed prior to seeking written consent.

Following provision of written consent, the following information and procedures will be recorded and performed as part of the screening assessments:

- Demographics including gender, race/ethnic origin, age
- Review of any recent symptoms (including cough, weight loss, fever, night sweats, diarrhoea, dysuria)
- A comprehensive medical history including a systematic inquiry, a family history, and a history of allergies
- Prescribed and non-prescribed medication received in the past 3 months
- Confirmation of contraception use in female subjects of childbearing age*
- Vital signs, including blood pressure, pulse rate, and axillary body temperature
- Physical examination
- Height, weight, and mid upper arm circumference

* Continuous use of an effective method of contraception until 2 months after receiving the final dose of unithiol is required for female participants of childbearing age (unless permanently sterile following hysterectomy). For those with no contraception, they will be referred for contraception at the relevant health facility. For female participants, we will ask them to attend with their family planning records for verification. Effective contraception is defined as a contraceptive method with failure rate of less than 1% per year when used consistently and correctly, in accordance with the product label. Examples of these include combined oral contraceptives, injectable progestogen, implants of etenogestrel or levonorgestrel,

intrauterine device or intrauterine system, male condom combined with a vaginal spermicide (foam, gel, film, cream, or suppository), and male condom combined with a female diaphragm, either with or without a vaginal spermicide (foam, gel, film, cream, or suppository).

The following investigations will be undertaken:

- Resting 12 lead ECG
- For female subjects aged 18 to 64 years: Pregnancy test
- Full blood count
- Sodium, potassium, urea, and creatinine
- Alanine aminotransferase (ALT) and bilirubin
- Urine dip (protein, blood, or glucose)
- Random blood glucose
- Viral serology (HBSAg, Hepatitis C virus serology, HIV1 & 2 antigen antibody)
- Malaria film and malaria rapid test

Once all the above information has been collected, the Investigator will decide if it is appropriate for the subject to be enrolled into the study. Data on all consenting screened subjects will be recorded and the reason for participant exclusions will be documented.

The investigational medical product (IMP)

The IMP will be Dimaval® oral capsules and solution for injection sourced from Heyl (the manufacturer).

Product names

International non-proprietary name: unithiol

Commercial Name: Dimaval®

Synonyms: DMPS, sodium (DL)-2,3-dimercaptopropane-1-sulphonate, sodium 2,3–66 dimercaptopropanesulphonate

IUPAC name: Sodium D,L-2,3-dimercapto-1-propanesulphonic acid

CAS No.: 4076-02-2

Chemical formula: H2C(SH)-HC(SH)-H2CSO-3Na.H2O

Relative molecular mass: 228.28 (monohydrate)

Conversions: 1g = 4.4mmol; 1mmol = 228.3mg; 1g/L = 4.4mmol/L; 1mmol/L = 0.228g/L

Formulations

The oral form of Dimaval will consist of 100mg capsules. According to the summary of product characteristics, each capsule contains 108.56 mg (RS)-2,3-bis(sulfanyl)propane-1-sulfonic acid, sodium salt 1 H_2O corresponding to 100 mg DMPS sodium salt[26].

The Dimaval solution for injection consists of 5mL volume ampoules containing 250 mg of DMPS-Na. According to the summary of product characteristics, each ampoule contains 271.4 mg (RS)-2,3-Bis(sulfanyl)propane-1-sulfonic acid, sodium salt 1 H_2O (DMPS sodium salt 1 H_2O) corresponding to 250 mg (RS)-2,3-Bis(sulfanyl)propane-1-sulfonic acid, sodium salt (DMPS-Na)[37].

Dose

Participants will be dosed in cohorts of 8. Cohorts for single oral doses are abbreviated as ‘C’, single intravenous dose cohorts as ‘CIV’ and multiple oral dosing cohorts as ‘CM.’ There will be four single ascending oral dosing cohorts (C1-C4), two intravenous dosing cohorts (CIV1 and CIV2) and two multiple dosing cohorts (CM1 and CM2). The anticipated dose escalations are summarised in Table 2. The dosing regimen in Table 2

Table 2. Summary of dosing regimens.

**Single dosing *(and CIV1)***		
**Cohort name**	**Number of subjects**	**Dose**
C1	8	300 mg oral
C2	8	900 mg oral
C3	8	1200 mg oral
C4	8	1500mg oral
CIV1	8	3 mg/kg intravenous single dose
**Multiple dosing *(and CIV2)***		
CM1	8	300–1200 mg oral qds for 2 to 3 days
CM2	8	1500 mg oral (or highest tolerated dose from single dosing) qds for 2 to 3 days
CIV2	8	Depends on emerging data (5–10 mg/kg intravenous) single dose

Additional dosing cohorts may be added depending on emerging safety data. Eight cohorts and the above dose escalations represent the maximum dose increases that would occur assuming reassuring safety data.

represents the anticipated dose escalations that will be followed should the DSMB have no safety concerns. The highest single oral dose of unithiol that will be administered is 1,500 mg. Any increase above 1,500 mg would require approval from the Sponsor and the ethics and regulatory committees. Following review by the DSMB, dose levels may be repeated, smaller increments in dose escalation may be adopted or dose levels may be lower than the preceding dose level and, therefore, additional cohorts may be included. The DSMB will make these decisions based on adverse event data.

Justification for the proposed dose, route of administration, dosage regimen and treatment period

The starting dose for the oral single ascending dose stage will be 300 mg. This is a routine dose recommended for the treatment of heavy metal poisoning[35,38]. The maximum dose that will be administered during the oral single ascending dose stage will be 1,500 mg. This dose has been selected based on pre-clinical in-vitro assays that have shown that high concentrations of unithiol are required to inhibit the pro-coagulant effects of snake venom[21]. In this experiment, 150 µM of unithiol was required to prevent consumption coagulopathy. Given that previous in-human pharmacokinetic analysis has identified a C_{max} of 25 µM following administration of 300 mg oral[33], it has been estimated that a dose of 1,500 mg would be required to achieve a peak plasma concentration sufficient to inhibit coagulopathy. Published reports of dose levels of unithiol equivalent or higher than 1,500 mg oral suggest that this dose is safe[24,31,32].

The dose that will be given to the first intravenous dosing cohort (CIV1) will be 3 mg/kg. This has been selected as it is at the lower range of the routinely recommended dose for acute heavy metal poisoning (3–5 mg/kg)[27,39]. Previous pharmacokinetic analyses have shown that 3 mg/kg intravenous achieves an initial plasma concentration of 100 µM[27]. At least one further intravenous dosing cohort will be included. The dose administered will be decided by the DSMB based on emerging data from the first stage of the trial. This dose is expected to be 5 mg/kg, although a dose of up to 10 mg/kg may be selected, depending on emerging safety and pharmacodynamic data.

For the multiple dosing cohorts (CM1 and CM2), the study team will select doses that show the optimum profile for treating snakebite, based on safety, tolerability and any available pharmacokinetic and pharmacodynamic data. If the pharmacodynamic data shows a dose response relationship and is deemed to demonstrate sufficient reliability, the investigators will aim to select CM1 based on a minimum dose associated with adequate *ex vivo* inhibition of SVMPs. The CM2 dose will be selected by the investigators based on the highest dose (between 300 and 1,500mg oral) that is safe and tolerated during single dosing. The preliminary pharmacokinetic data from the single dosing stage will be modelled to identify CM1 and CM2 doses and dose intervals, that will not cause accumulation of the study drug. Ideally, a regimen of no more than four times per day dosing would be preferred (unlike in heavy metal poisoning where 1–2 hourly dosing is used), to optimize adherence to the regimen.

Dose escalation schedule

In every cohort, subjects will be dosed at least 30 minutes apart. In cohorts C2, C3 and C4, 1 participant will receive a sentinel dose prior to the remaining 7 participants being dosed. Minimum time-periods of observation between dose escalations are summarised in Figure 1a and Figure 1b.

C2 begins with a sentinel dose and the remaining 7 subjects will be dosed at least 24 hours after this. C3 will begin with a sentinel dose and the remaining 7 subjects will be dosed 72 hours after this. Cohort C4 will be dosed a minimum of 1 week after C3 and will also commence with a sentinel dose followed by 3 days until the remaining 7 subjects are dosed. The CIV cohort will be dosed in tandem with C3. Dose escalation decisions will not be dependent on the results of the pharmacodynamic assay, as this analysis is exploratory and has not been validated. Between the single dosing stage and the multiple dosing stage, preliminary pharmacokinetic data will be made available. Multiple dosing will only proceed once the DSMB have reviewed adequate pharmacokinetic data from the single dosing stage to be satisfied that the proposed dosing regimen will not lead to accumulation of the study drug. A minimum of one week after the final dose is given in CM1, dosing of CM2 will commence. The flowchart (Figure 1) summarises the process of dose escalation.

Sample size determination

As this is a phase I clinical trial, with a primary aim to evaluate the safety of the escalating unithiol doses, a sample size calculation has not been conducted. Participants will be dosed in groups of eight, which is a commonly used design in phase I clinical trials[40].

The number of cohorts will be guided by the safety and tolerability of unithiol. Additional cohorts may be added if adverse events occur that, following review by the DSMB, warrant smaller increments in dose escalation or expansion at a dose level.

Randomisation procedure

Subjects will be allocated to a dosing cohort using stratified permuted block randomization. Stratification will be according to sex and age. Following the screening visit (and prior to attendance at the in-patient facility), participants will be asked to attend to receive the results of the clinical investigations, to be informed of their eligibility and to be informed of their allocation following randomization. Throughout the study, a minimum of four participants (randomly selected) will be on stand-by for each dosing cohort – to replace any participants that cannot be dosed. There is no placebo group and therefore this is not a randomized controlled trial. Participants in dosing cohorts that involve sentinel dosing will undergo a second step of randomization, to allocate individuals for sentinel dosing. The study team member responsible for randomizing subjects

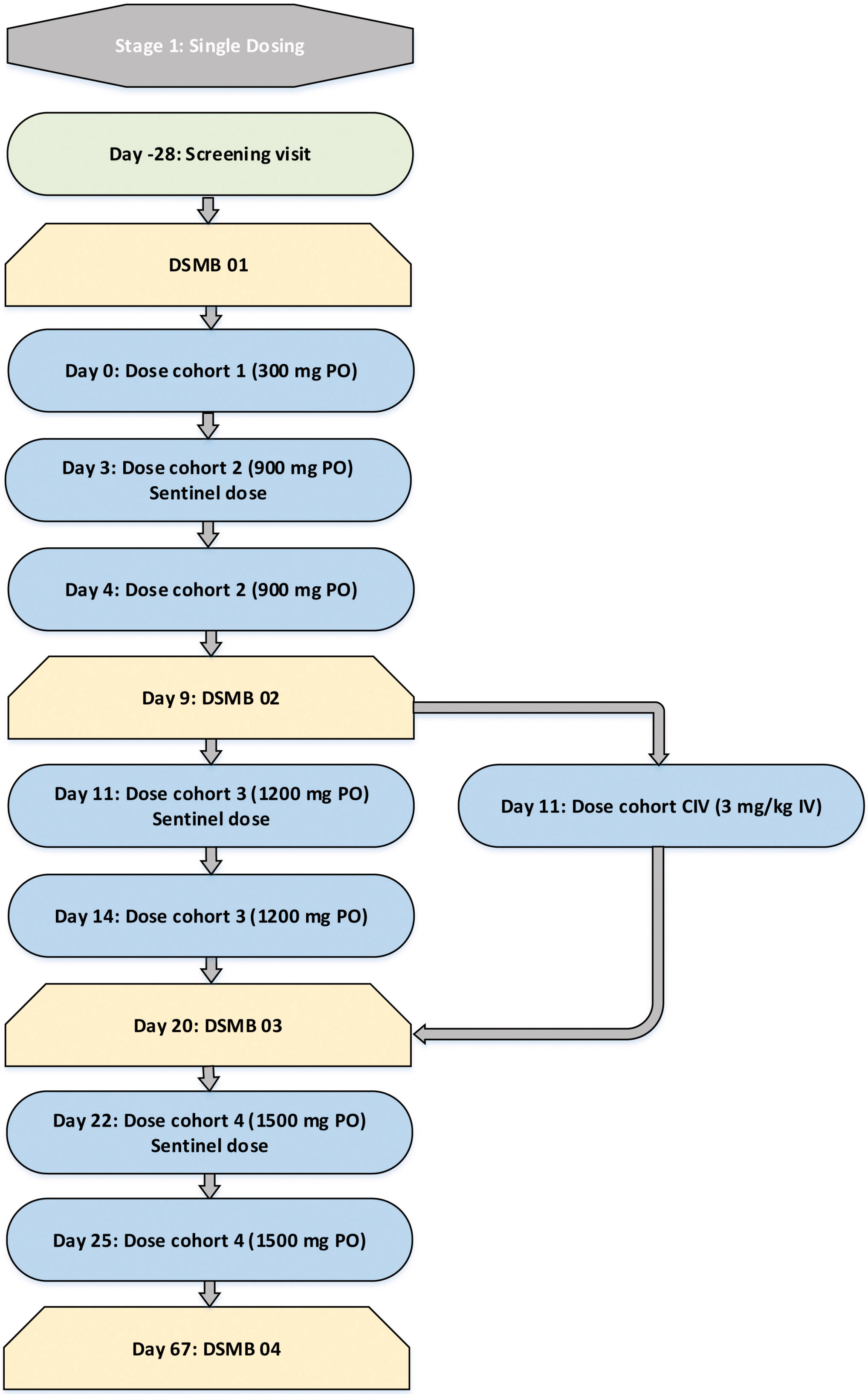

Figure 1a. **Single Dosing Escalation Strategy.**

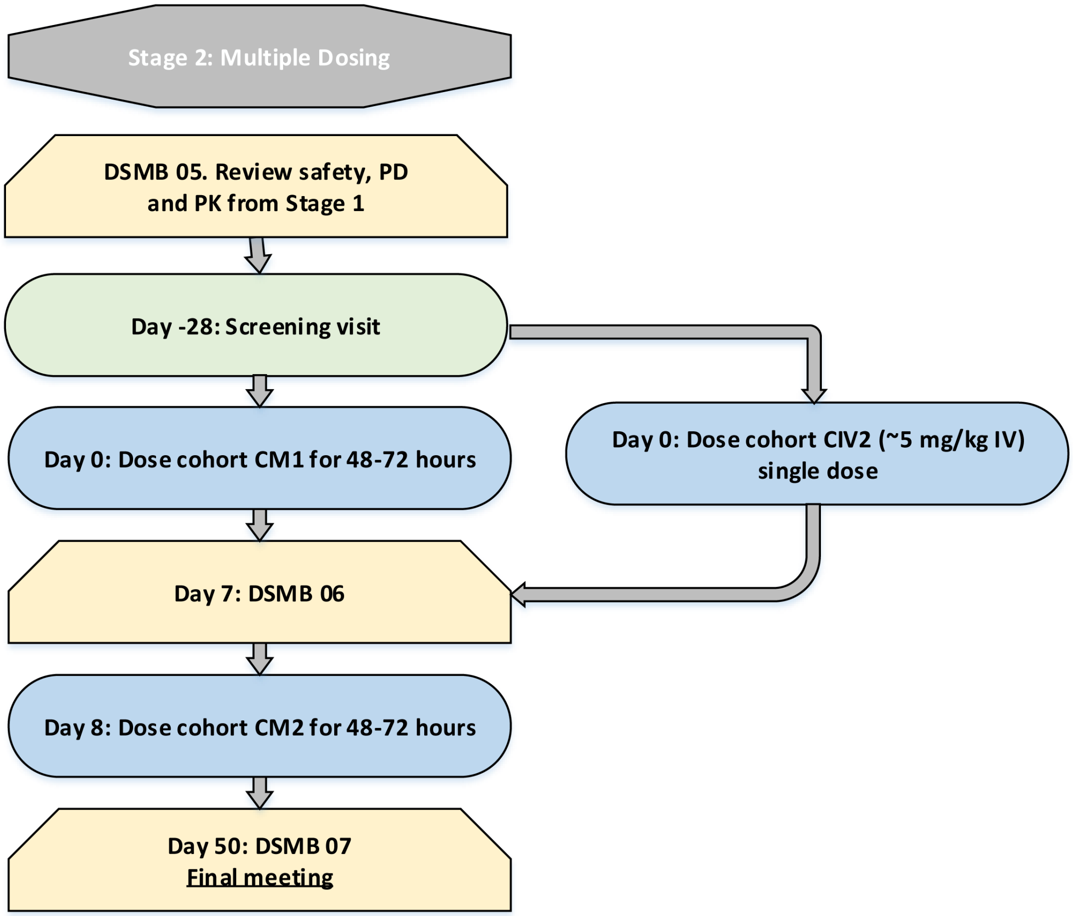

Figure 1b. Multiple dosing escalation strategy. DSMB, Data and Safety Monitoring Board; IV, intravenous administration; PO, oral administration. Days represent the minimum period between dose escalations. Longer intervals may be required. Time interval until DSMB meetings represent the earliest possible meeting and later meetings may occur.

will be blinded to the subject's identity and any information related to the subject except for their sex and age. Once randomization is complete, cohort allocation will be unconcealed. The study team and the participants will not be blinded.

Enrolment into the trial will begin the night before dosing. The participant will be reviewed by a study team member in the in-patient facility. Any concerns or questions the participant may have will be answered. Any participants that do not arrive or cannot be dosed due to becoming ineligible (e.g., due to illness) will be replaced, as detailed below.

Trial procedures

Table 3a and Table 3b summarise the daily study procedures for the single and multiple dosing stages, respectively (additional assessments or blood draws may be added based on emerging data or if clinically indicated). Participants will be admitted to an in-patient facility the night prior to dosing and monitored until 24 hours after their last dose of unithiol and will be reviewed as an out-patient on days 2, 5 and 42 (with telephone follow-up at 6-months).

Procedures for collection of clinical samples

Timing and volume of clinical samples

Figure 2a, Figure 2b and Figure 2c summarize the blood and urine sampling protocol for the single and multiple dose stages, respectively. The timing of blood and urine samples may be adjusted, and any such changes will be provided to the approval committees via a written notification. This would be because of emerging safety, pharmacokinetic or pharmacodynamic data.

Justification of frequency and volume of blood sampling

Frequent blood sampling is required to understand the pharmacokinetic profile of unithiol. As this drug has a short-half-life, less frequent drug monitoring would lead to inaccurate estimates of the C_{max} and AUC (area under the curve). For the pharmacodynamic analysis, it is important for us to understand the duration of efficacy. As unithiol is rapidly metabolised and excreted, the duration of venom inhibition may be short. By understanding this duration, better decisions of optimal dose frequency can be obtained. Blood volumes are based on required amounts for each assay, with the need for storage in duplicate being accounted for. Frequency of sampling and blood volumes

Table 3a. Study schedule – single dosing regimen.

Activity	Screening (Day -28 to Day -2)	Randomisation (Day -27 to Day -2)	Day -1	Day 0	Day 1	Day 2	Day 5	Day 42	6-month telephone visit
In-patient stay			■	■	■				
Informed consent	■								
Inclusion/exclusion criteria	■	■	■						
Demographic data (including smoking history)	■								
Medical history	■								
Viral serology	■								
Pregnancy test[a]	■		■				■	■	
Provide results to investigations		■							
Randomization		■							
**Study residency:**									
Check in			■						
Check out					■ [b]				
Non-residential visit						■	■	■	
Study drug administration				■					
**Safety and tolerability:**[c]									
Adverse event recording[d]	■	■	■	■	■	■	■	■	■
Vital signs (including blood pressure and pulse rate)	■		■	■	■	■	■	■	
Axillary body temperature	■		■	■	■	■	■	■	
12 lead ECG[e]	■				■				
Clinical laboratory evaluations (including haematology, clinical chemistry, and urinalysis)	■		■	■	■	■	■	■	
Body weight (and height at first visit)	■		■	■	■				
Physical examination	■		■	■	■	■	■	■	
Concomitant medication	■		■	■	■	■	■	■	
**Blood sampling**									
Pharmacokinetics:			■	■	■	■			
Pharmacodynamics:			■	■	■				

Table 3b. **Study Schedule – Multiple dosing regimen.**

Activity	Screening (Day -28 to Day -2	Randomisation (Day -27 to -2)	Day -1	Day 0	Day 1	Day 2	Day 3	Day 5	Day 42	6-month telephone visit
In-patient stay			■	■	■	■	■			
Informed consent	■									
Inclusion/exclusion criteria	■	■	■							
Demographic data (including smoking history)	■									
Medical history	■									
Viral serology	■									
Pregnancy test[a]	■		■					■	■	
Provide results to investigations		■								
Randomization		■								
**Study residency:**										
Check in			■							
Check out							■ [b]			
Non-residential visit								■	■	
**Study drug administration[f]**				■	■	■				
**Safety and tolerability:[c]**										
Adverse event recording[d]	■	■	■	■	■	■	■	■	■	■
Vital signs (including blood pressure and pulse rate)	■		■	■	■	■	■	■	■	
Axillary body temperature	■		■	■	■	■	■	■	■	
12 lead ECG[e]	■						■			
Clinical laboratory evaluations (including haematology, clinical chemistry, and urinalysis)	■		■	■	■	■	■	■	■	
Body weight (and height at first visit)	■		■	■	■	■	■			
Physical examination	■		■	■	■	■	■	■	■	
Concomitant medication	■		■	■	■	■	■	■	■	
**Blood sampling**										
Pharmacokinetics:			■	■	■	■	■	■		
Pharmacodynamics:			■	■	■	■	■	■		

ECG, electrocardiogram.

[a] Females aged 18 to 64 years. Performed in urine.

[b] In house stay until 24 hours post-dose.

[c] The timings of all measurements to be performed during the study may be subject to change based on the ongoing review of the safety, tolerability, pharmacokinetic and pharmacodynamic results.

[d] Serious adverse events will be recorded from enrolment, and adverse events will be recorded from dose administration until the final follow up Visit.

[e] Resting 12 lead ECG: at Screening; and Day 1 post dose and prior to discharge from inpatient facility. Additional ECG recordings will be taken at the discretion of the responsible clinician.

[f] Doses anticipated to be administered four times per day for up to 72 hours.

SINGLE ORAL DOSING COHORTS		Screening	Admission	Follow-up												Total volume of blood samples	Volume blood per participant
Procedure	Day	-28	D-1	D0	D0	D0	D0	D0	D0	D0	D0	D1	D2	D5	D 42		
	Time (hours)	-	-	0	1	2	3	4	5	8	12	24	48				
Drug intake				X													
Venepuncture (mL)	Full blood count	4	4									4	4	4	4	24	219.5
	Biochemistry	5	5									5	5	5	5	30	
	Hepatitis B and C																
	PD sample			8	8	8	8	8	8	8	8	8	8			80	
	PK sample			8.5	8.5	8.5	8.5	8.5	8.5	8.5	8.5	8.5	8.5			85	
	Urine PK		x	Continuous collection												NA	
	Urine βHCG	x	x											x	x	NA	
	HIV test (RDT)	Drop														0.1	
	Malaria RDT	Drop	Drop													0.2	
	Malaria film	Drop	Drop													0.2	
Total blood volume per day (mL)		9.3	9.2	132								25.5	25.5	9	9		
Day		D-28	D-1	D0								D1	D2	D5	D42		
Blood collections per day		1	1	8								1	1	1	1		

Figure 2a. Blood and urine sampling times and volumes during inpatient stay and out-patient follow-up – single oral dosing.

may increase if adverse events occur or if the emerging pharmacokinetic or pharmacodynamic data demonstrate unanticipated findings. Nevertheless, the total blood volumes will not exceed 500 mL per 3 months, as per 2001 Kenya Ministry of Health Blood Transfusion Guidelines.

Interim visits

Subjects will be admitted to the inpatient facility the day prior to dosing and monitored there by clinical study staff for a minimum of 24 hours following the last dose of unithiol. The rationale for this time window post dosing is based on the short half-life of unithiol, which is 4 hours, as well as the pre-existing safety data[33].

Subjects will be asked to visit the KEMRI-CGMRC or an appropriate facility on days 2, 5 and 42 for single dosing and days 5 and 42 for multiple dosing. They will be monitored for adverse events and have samples collected at these visits (Table 3 and Figure 2). The final planned review will be via telephone at 6 months (with the option for a face-to-face review, if necessary). If a participant cannot be contacted by telephone, arrangement for a home visit will be made, to ensure that the participant remains safe and well.

Unscheduled visits

If the participant is unwell, they will be strongly encouraged to seek treatment with the study team and given emergency contact numbers to facilitate this. Out of hours' participants will be advised to contact the study clinician and in the event of an emergency, to attend Kilifi County Hospital. Subjects will be required to be resident in KHDSS during the study. Medical emergency plans will be in place with the possibility to admit patients to the Aga Khan Hospital in Mombasa, should intensive care be required. Transport related to unscheduled (and scheduled) visits will be reimbursed.

End of treatment visit

The final visit for each cohort will be at 6 months. As per Table 3 and Figure 2, this will include adverse event recording. If adverse events have occurred, the final visit may be later, at the discretion of the study team. If adverse events are ongoing, follow-up will be continued until the event resolves or until the condition has stabilised.

Follow-up visit(s)

Each subject will participate in a single treatment period only, and they will be in an in-patient facility starting from the day

SINGLE IV DOSING COHORTS		Screening	Admission		Follow up									Total volume of blood samples	Volume blood per participant
Procedure	Day	D-28	D-1	D0	D0	D0	D0	D0	D0	D1	D2	D5	D 42		
	Time (hours)	-	-	Pre-dose	End of infusion	1	2	5	10	24	48				
Drug intake				X											
Venepuncture (mL)	Full blood count	4	4							4	4	4	4	24	186.5
	Biochemistry	5	5							5	5	5	5	30	
	Hepatitis B and C														
	PD sample			8	8	8	8	8	8	8	8			64	
	PK sample			8.5	8.5	8.5	8.5	8.5	8.5	8.5	8.5			68	
	Urine PK		x		Continuous collection									NA	
	Urine βHCG	x	x									x	x	NA	
	HIV test (RDT)	Drop												0.1	
	Malaria RDT	Drop	Drop											0.2	
	Malaria film	Drop	Drop											0.2	
Total blood volume per day (mL)		9.3	9.2	99						25.5	25.5	9	9		
Day		D-28	D-1	D0						D1	D2	D5	D42		
Blood collections per day		1	1	6						1	1	1	1		

Figure 2b. Blood and urine sampling times and volumes during inpatient stay and out-patient follow-up – single intravenous dosing.

before dosing until 24 hours after last dose. The multi-dosing regimen is for a period of up to 72 hours and participants will therefore be monitored for 72 to 96 hours. Out-patient visits at KEMRI-CGMRC or the in-patient facility will be arranged on day 2, 5 and day 42 post-dose for the single dosing group. The multiple dosing group will be reviewed as an out-patient on day 5 and day 42. All participants will undergo telephone follow-up at 6 months. Depending on emerging data, additional visits (including additional telephone consultations) may be required.

Study restrictions

Dose escalation holding criteria

If any of the following scenarios occur within a group with reasonable possibility of a causal relationship with unithiol, dose escalation will be stopped pending safety review, prior to higher dose levels being evaluated. In the case a serious adverse event occurring, this will be reported in accordance with section 11.7 of this protocol, including reporting to the Sponsor and the DSMB within 48 hours.

- Clinically relevant signs or symptoms or intolerable adverse events of similar nature occur in 2 or more subjects in a group that in the opinion of the Investigator warrant stopping of dose escalation
- One or more subjects report a serious adverse event considered by the Investigator to be related to the study drug
- Two or more severe adverse events occur which are considered by the Investigator to be related to the study drug
- Dose escalations will only occur after a minimum period of days, depending on the dose received (as specified in Figure 1a and Figure 1b).

Cohort dosing holding criteria

If any of the following scenarios occur within a group with reasonable possibility of a causal relationship with unithiol, further dosing within a cohort will be stopped pending review by the Data Safety Monitoring Committee.

MULTIPLE DOSING COHORTS		Screening	Admission	Follow-up															Total volume of blood samples	Volume blood per participant
Procedure	Day	-28	D-1	D0	D0	D0	D0	D0	D0	D1	D1	D1	D1	D1	D2	D3	D5	D42		
	Time (hours)	-	-	0	2	4	6	12	18	24	30	32	34	36	48	72	-	-		
Drug intake				X			X	X	X	X	X									
Venepuncture (mL)	Full blood count	4	4							4					4	4	4	4	28	228.5
	Biochemistry	5	5							5					5	5	5	5	35	
	Hepatitis B and C																			
	PD sample			8	8	8	8	8		8	8	8	8	8					80	
	PK sample			8.5	8.5	8.5	8.5	8.5		8.5	8.5	8.5	8.5	8.5					85	
	Malaria film	drop	drop																0.2	
	Malaria RDT	drop	drop																0.2	
	HIV test (RDT)	drop																	0.1	
Urine (mL)	Urine PK		x	Continuous collection															NA	NA
	Urine βHCG	x	x														x	x	NA	
Day		D -28	D -1	Day 0						Day 1					D2	D3	D5	D42		
Total blood volume per day (mL)		9.3	9.2	82.5						91.5					9	9	9	9		
Blood collections per day		1	1	5						5					1	1	1	1		

Figure 2c. Blood and urine sampling times and volumes during inpatient stay and out-patient follow-up – multiple oral dosing. βHCG, Beta-human chorionic gonadotrophin; D, day; NA, not applicable; RDT, rapid diagnostic test; PD, pharmacodynamics; PK, pharmacokinetics. Figure 2c is based on an assumed dosing duration of 30-hours. This may vary depending on emerging data and could extend up to a maximum of 72-hours.

- Clinically relevant signs or symptoms or intolerable adverse events of similar nature occur in 2 or more subjects in a group that in the opinion of the Investigator warrant stopping of dose escalation
- One or more subjects report a serious adverse event considered by the Investigator to be related to the study drug*
- Two or more severe adverse events occur which are considered by the Investigator to be related to the study drug

* If a serious adverse event were to occur during sentinel dosing, then dosing of other subjects would be held until review by the DSMB.

Should the threshold for a holding rule be met, there would be a study hold then an *ad-hoc* DSMB meeting would be convened before further dosing. All safety follow-ups would continue as planned.

Concomitant medication

This will be assessed at the time of the first screening visit and the participants will be asked if they have taken any medication within the past 3 months. Medications taken within 3 months of the screening study will not necessarily result in exclusion, rather this will be at the discretion of the principal investigator (PI). The study team will emphasize to participants the importance of the medical team overseeing clinical care during the study period. Participants will be encouraged to contact the study team for all medical attention during the study. If any medication is required, the name, strength, frequency of dosing and reason for use will be documented in the participants source data. Participants will be asked to phone the study team should they require medical review or change in contraception.

Appropriate contraceptive medication is an acceptable concomitant medication and is an inclusion criterion for women of childbearing age (as per section 6.1.2 Screening). Should a participant require a prescription for contraception they would be referred to KCH services or their local health Centre/dispensary.

Patient obligations

Diet

Standard meals will be provided for all subjects whilst they are resident at the trial unit. Dietary requirements will be confirmed at the screening visit. Dietary intake is unlikely to affect elimination of unithiol as the drug is renally excreted and is not subject to metabolism by liver enzymes.

During periods of intensive pharmacokinetic blood sampling (the day of dosing), meals will be identical for each group. Otherwise, meals will not be restricted.

Alcohol
Subjects will adhere to the following alcohol restrictions:

- Alcohol is not permitted from 36 hours before check-in until discharge from the inpatient facility. Participants will be made aware of this. There will not be active efforts to police this restriction.

- Alcohol is not permitted 36 hours prior to non-residential visits.

- Alcohol intake is unlikely to have a significant effect on the assessment of safety or pharmacodynamic or pharmacodynamic during this trial. There may be a modest (diuresis induced) increase in renal excretion of unithiol following alcohol intake.

Smoking
No restrictions. Standard health and safety procedures (such as not smoking in the health facility) will be in place.

Exercise
No restrictions.

Blood donation
Subjects must not donate blood from enrolment until 3 months after the final follow-up visit.

Contraception
Use of an effective method of contraception for the duration of study (and until 2 months after last receiving the study drug) for female participants aged 18–64 years (unless permanently sterile following hysterectomy). For those not on effective contraception, referral to an appropriate family planning service will be offered. For female participants, we will ask them to attend with their family planning records for verification. Effective contraception is defined as a contraceptive method with failure rate of less than 1% per year when used consistently and correctly, in accordance with the product label. Examples of these include combined oral contraceptives, injectable progestogen, implants of etenogestrel or levonorgestrel, intrauterine device or intrauterine system, male condom combined with a vaginal spermicide (foam, gel, film, cream, or suppository), and male condom combined with a female diaphragm, either with or without a vaginal spermicide (foam, gel, film, cream, or suppository).

Prior/concomitant therapy
The study team will emphasize to participants the importance of the medical team overseeing clinical care during the study period. Participants will be encouraged to contact the study team for all medical attention during the study. If any medication is required, the name, strength, frequency of dosing and reason for use will be documented in the participants source data. Participants will be asked to phone the study team should they require medical review or change in contraception.

Prohibited medication
Participants will be encouraged to seek medical care from the study team during their follow-up period. Participants will be discouraged from taking over the counter medications and medications from other providers except in urgent situations, where delay to treatment could be harmful. Receipt of a concomitant medication for an acute illness would normally not lead to exclusion from the trial.

Participant withdrawals

Subject withdrawal criteria
Subjects will be withdrawn for multiple reasons including some of those listed below:

- Any clinically relevant signs, symptoms, or adverse events that in the opinion of the Investigator warrant subject withdrawal

- Non-compliance with the study restrictions, as considered applicable by the Investigator

- Loss to follow up (applies to a subject who consistently does not return for protocol study visits, is not reachable by telephone or any other means of communication and/ is not able to be located).

- At the discretion of the Investigator, if inter-current illnesses occur that may invalidate the study data, if the subject was enrolled in violation of the study protocol, or if a significant protocol violation occurs (note, asymptomatic malaria infection is not anticipated to require exclusion from the study, although subjects will be monitored closely)

- Subject decision

- On the advice of DSMB

Managing withdrawals
- If a subject is withdrawn, the reason will be recorded in the case report form (CRF). If withdrawal is the result of a serious adverse event (AE), the investigator will offer to arrange for appropriate specialist management of the problem and the ethical committee, DSMB and regulatory authority will be informed in a timely manner. The extent of follow up will be determined by a medically qualified investigator but will be at least for the whole study period. Subjects withdrawn prematurely for any reason will not receive further doses of unithiol, although they will be encouraged to come back to the clinic for safety evaluation. Additionally, participants will not receive the IMP if they do not meet the eligibility criteria.

- If a participant withdraws from the study, blood samples collected before his/her withdrawal from the trial will be used/stored unless the participant specifically requests otherwise. In all cases of subject withdrawal, apart from those of complete consent withdrawal, long-term safety data collection for participants, including some procedures such as safety blood investigations, will continue so far as the participant is willing to consent. Where

participants withdraw consent for follow up, this will be respected and follow up will be discontinued.

- If a volunteer withdraws/is withdrawn from the study after receiving unithiol, they will be encouraged to continue to complete the recommended period of safety monitoring. If the subject requests to leave after taking unithiol but before being discharged from the inpatient facility, they will be offered telephone follow-up.

Replacing withdrawn participants
For each dosing protocol (single dosing and multiple dosing), a minimum of four participants (randomly selected) will be on stand-by – to replace any subject withdrawals or non-attendances. The stand-by subjects will have undergone screening and written consent and will be asked to be available during the day before and the day of dosing and will be reimbursed for lost wages accordingly. Subjects that complete dosing and monitoring at the inpatient facility, but who do not attend out-patient follow-up (Day 2, 5 and 42) will not be replaced.

Storage, packaging, dispensing, and administration of the investigational medicinal product
Storage
The capsules and solution for injection will be stored in a pharmacy with temperature regulation as this should not exceed 25 °C. The capsules will be kept dry as per recommendations on summary of product characteristics. The shelf-life for the commercially available pharmaceutical preparation Dimaval® is 3 years for the capsules and the ampoules. The expiry date is stated on each package.

Packaging and labelling
Packaging and labelling of the investigational medical product will comply with the Kenya Pharmacy and Poisons Board (PPB) labelling requirements, as per their guidelines for the conduct of clinical trials in Kenya.

Dispensing procedures
The study drug will be stored in the pharmacy at KCH according to the manufacturer's specifications. The drug, dose and subject identity will be confirmed by one pharmacist or pharmacy technician and one study team member.

Dose administration
Oral unithiol will be administered with 240mL of water and will be swallowed as whole tablet(s). Food will not be consumed for at least one-hour after dosing, as per product literature.

Intravenous dosing will be infused over a minimum period of 5 minutes, as per a previous study[27], with the subject lying flat with blood pressure monitoring during the infusion, at 5 minutes and at 15 minutes (more frequent monitoring will be provided if the responsible clinician identifies a clinical need). The solution for infusion will be prepared according to the product literature.

Treatment compliance
All doses of the IMP will be administered under direct observation by a trained study team member. Participants can withdraw from the study if they no longer wish to receive the medication.

Overdosage
Overdose of unithiol will be prevented as subjects will not have access to the medication, except for the individual doses they are to receive. The IMP will be stored securely in a locked drug cabinet. Doses will be confirmed by two study team members before administration.

In the rare event of overdose, participants will be managed with necessary supportive measures (including intravenous fluids) and the PI, in discussion with clinical investigators, hospital staff and the DSMB will decide whether transfer to a facility with intensive care and dialysis/haemofiltration facilities is necessary.

Rescue medication
There is no antidote to unithiol. Dialysis does remove the drug.

Assessment of pharmacokinetics and pharmacodynamics
Pharmacokinetics
The secondary outcome is to define the pharmacokinetic profile of unithiol. Although most of the pharmacokinetic analysis will be completed after all the subjects have completed follow-up in the trial, preliminary pharmacokinetic data will be made available prior to commencing the multiple dosing stage of the trial. The purpose of this preliminary data will be to predict a dose and dosing interval that will provide consistent plasma drug levels and not pose a risk of accumulation of the study drug following multiple dosing. Multiple dosing will only proceed once the DSMB have reviewed adequate pharmacokinetic data from the single dosing stage to be satisfied that the proposed dosing regimen will not lead to accumulation of the study drug.

This will be undertaken in collaboration with the University of Liverpool. Samples will be shipped to the Liverpool Bioanalytical Facility (GCP Laboratories, William Henry Duncan Building, 6 West Derby Street, University of Liverpool, L7 8TX) for the purposes of measuring drug concentrations. This is necessary to fulfil regulatory requirements for pharmacokinetic analysis. Drug concentrations will be quantified by validated liquid chromatography-mass spectrometry.

Pharmacodynamics
An exploratory outcome is to assess the efficacy of unithiol to inhibit the pathogenic actions of venom, *ex vivo*. If the pharmacodynamic assay produces results that are deemed reliable, then pharmacodynamic data from the single dosing stage could be used to guide the dosing regimen for the multiple dosing stage. If the pharmacodynamic assay does not produce useful results, then dose and dosing frequency decisions will be made based on safety and preliminary pharmacokinetic data.

Ex vivo assays will be developed for the purposes of establishing a dose-response and estimating a pharmacodynamic profile. These assays will rely on spiking subjects' plasma with venom *ex vivo* and measuring activity of snake venom metalloproteinases. These assays are being developed for the purposes of this study, and a published validated assay does not exist. It would not be appropriate to assign these assays as primary outcome variables as they are experimental and represent an indirect estimate of efficacy.

These assays will continue to be refined during this study. They primarily represent a tool to compare venom metalloproteinase inhibition between subjects receiving different doses of unithiol, rather than a direct measure of efficacy. Where possible, these assays will be conducted and refined in the laboratory facilities at KEMRI-CGMRC. Split samples will also be shipped to Liverpool School of Tropical Medicine Centre for Snakebite Research & Interventions (LSTM CSRI).

Study governance: local safety monitor and data safety monitoring board

Local safety monitor

- The local safety monitor (LSM) will be a clinician resident in Kilifi (therefore likely to be linked to or a staff member of KWTRP) but independent of the study team. The LSM will act as a semi-independent assessor of participants experiencing important safety events at the request of the DSMB and will provide their observations to the DSMB and/or local clinicians or study team members where appropriate.

Data and safety monitoring board

- The DSMB will include a minimum of 3 members with at least 1 clinician with expertise of the local context and 1 statistician.

- The DSMB charter has been uploaded the Harvard Dataverse[41]

- At least one of the following investigators will attend DSMB meetings: Dr Abouyannis, Dr Hamaluba or Professor Lalloo (either remotely or in-person). The DSMB will have access to all relevant data on administration of unithiol. The independent local clinician will have expert knowledge of the Kenyan context. The DSMB will be convened at the start of the study before unithiol is administered, to review the protocol and their responsibilities. Planned DSMB meeting will occur before every dose escalation, although the DSMB can hold additional *ad-hoc* meetings if they deem it necessary. A local safety monitor (LSM) will also be appointed prior to this to provide independent safety assessments of participants. The DSMB will review:

- all SAEs/SUSARs (serious adverse events/suspected unexpected serious adverse reactions)

- SAEs will be reviewed alongside an updated summary of all severe adverse events reported to that date.

- Clinically relevant signs or symptoms or intolerable adverse events of similar nature occurring in 2 or more subjects in a group

- Summaries of all adverse events will be reported to the sponsor and DSMB. However, investigators or the local safety monitor can request the DSMB to review any non-serious non-severe adverse events that raise concern. The Investigators will be responsible for reporting a summary of safety data to the Scientific and Ethics Review Unit (SERU) and PBB, as per their guidelines.

Assessment of safety

Adverse events (AEs)

Both solicited and unsolicited AEs will be recorded on the participant's CRF until the end of the 6-month follow-up period. The diagnosis, date and time of onset, outcome, severity, and relationship to unithiol administration will be established. Details of any treatment or concomitant interventions will be recorded.

If any pregnancies occur with 2 months of receiving unithiol, the participant will be referred for appropriate antenatal care. Unithiol has a short half-life and, as participants will undergo pregnancy testing, it is very unlikely that any pregnancies will occur whilst unithiol is detectable in the circulation. Nevertheless, if this were to occur during the trial the pregnant participant would be followed-up until term and (as a minimum) their baby would be examined by a paediatrician at 6-weeks following birth. Any congenital anomaly or birth defect would be reported as a serious adverse event.

During the in-patient admission, the study clinician will evaluate for solicited adverse events. Solicited AEs will also be elicited at every follow-up visit (day 2, day 5, day 42 and 6 months). Assessing the severity of adverse events will be the responsibility of a clinical or medical officer.

Solicited adverse events

Solicited adverse events are those that have been associated with unithiol and are listed in the summary of product characteristics. These are summarised in Table 4.

Definitions and monitoring of AEs

The definitions of adverse events (AE), adverse reactions (AR), serious adverse events (SAE), serious adverse reactions (SAR), and suspected unexpected serious adverse reactions (SUSAR) are available in Table 5.

Assessing the relationship between an adverse event and the investigational medicinal product

The causal relationship between an adverse event and the study drug is defined in Table 6.

Severity grading of adverse events

All adverse events will be severity graded using the definition shown in Table 7. Severity grading of abnormal vital signs will be categorised as shown in Table 8.

Follow-up of adverse events

Adverse events likely to be related to the study drug, whether serious or not, which persist at the end of the trial will be followed up by the investigator until their resolution or

Table 4. **Solicited adverse events.**

Category	Solicited adverse events
Symptoms	Nausea, weakness, loss of appetite, dysgeusia (change in taste), painful injection site, abdominal pain
Physical observations	Shivering, fever, skin reactions (itching rash), Stevens-Johnson's syndrome, hypotension (with intravenous unithiol)
Laboratory abnormalities	Leucopaenia, transaminitis, kidney injury (raised urea and creatinine)

Table 5. **Definitions of adverse events.**

Term	Definition
Adverse Event (AE)	Any untoward medical occurrence in a patient or clinical investigation subject occurring in any phase of the clinical study whether or not considered related to the study drug. This includes an exacerbation of pre-existing conditions or events, intercurrent illnesses, or drug interactions. Anticipated day-to-day fluctuations of pre-existing conditions, that do not represent a clinically significant exacerbation, will not be considered AEs. Discrete episodes of chronic conditions occurring during a study period will be reported as adverse events to assess changes in frequency or severity.<br>Unsolicited adverse events will be documented in terms of a medical diagnosis(es). When this is not possible, the AE will be documented in terms of signs and symptoms observed by the investigator or reported by the subject.<br>Pre-existing conditions or signs and/or symptoms (including any which are not recognized at study entry but are recognized during the study period) present in a subject prior to the start of the study will be recorded on the Medical History form within the subject's case report form (CRF).
Adverse Reaction (AR)	An untoward and unintended response in a participant to an investigational medicinal product which is related to any dose administered to that participant.<br>The phrase "response to an investigational medicinal product" means that a causal relationship between a trial medication and an AE is at least a reasonable possibility, i.e., the relationship cannot be ruled out.<br>All cases judged by either the reporting medically qualified professional or the Sponsor as having a reasonable suspected causal relationship to the trial medication qualify as adverse reactions. It is important to note that this is entirely separate to the known side effects listed in the SmPC. It is specifically a temporal relationship between taking the drug, the half-life, and the time of the event or any valid alternative aetiology that would explain the event.
Serious Adverse Event (SAE)	A serious adverse event is any untoward medical occurrence that:<br>• results in death<br>• is life-threatening<br>• requires inpatient hospitalisation or prolongation of existing hospitalisation<br>• results in persistent or significant disability/incapacity<br>• consists of a congenital anomaly or birth defect<br>Other 'important medical events' may also be considered serious if they jeopardise the participant or require an intervention to prevent one of the above consequences.<br>NOTE: The term "life-threatening" in the definition of "serious" refers to an event in which the participant was at risk of death at the time of the event; it does not refer to an event which hypothetically might have caused death if it were more severe.<br>"Hospitalisation" signifies that the subject has been detained (usually involving an overnight stay) at a hospital for observation and/or treatment above that routinely offered at the inpatient trial facility. When in doubt as to whether hospitalisation occurred or was necessary, the adverse event will be considered as serious. Hospitalisation for elective surgery or routine clinical procedures, which are not the result of an adverse event, will not be considered adverse events and should be recorded on a clinical assessment form and added to the CRF.
Serious Adverse Reaction (SAR)	An adverse event that is both serious and, in the opinion of the reporting Investigator, believed with reasonable probability to be due to one of the trial treatments, based on the information provided.
Suspected Unexpected Serious Adverse Reaction (SUSAR)	An adverse reaction, the nature or severity of which is not anticipated based on the applicable product information is considered as an unexpected adverse drug reaction. Where the adverse reaction is also considered to have a possible, probable, or definite relationship with the drugs given, and also meets the criteria for a serious adverse reaction, it is termed a Suspected Unexpected Serious Adverse Reaction (SUSAR). These events are subject to expedited reporting as for SAEs.

To avoid confusion or misunderstanding of the difference between the terms 'serious' and 'severe,' the following note of clarification is provided: 'severe' is often used to describe intensity of a specific event, which may be of relatively minor medical significance. 'Seriousness' is the regulatory definition supplied above.

Detailed guidance can be found here:

http://ec.europa.eu/health/files/eudralex/vol-10/2011_c172_01/2011_c172_01_en.pdf

Table 6. Categorizing a causal relationship.

Category		Definition
0	No Relationship	No temporal relationship to study product ***and***<br>Alternate aetiology (clinical state, environmental or other interventions); ***and***<br>Does not follow known pattern of response to study product
1	Unlikely	Unlikely temporal relationship to study product ***and***<br>Alternate aetiology likely (clinical state, environmental or other interventions) ***and***<br>Does not follow known typical or plausible pattern of response to study product.
2	Possible	Reasonable temporal relationship to study product; ***or***<br>Event not readily produced by clinical state, environmental or other interventions; ***or***<br>Similar pattern of response to this form of therapeutic
3	Probable	Reasonable temporal relationship to study product; ***and***<br>Event not readily produced by clinical state, environment, or other interventions ***or***<br>Known pattern of response seen with study product
4	Definite	Reasonable temporal relationship to study product; ***and***<br>Event not readily produced by clinical state, environment, or other interventions; ***and***<br>Known pattern of response seen with study product

Table 7. Severity grading of adverse events.

Grade	Definition
0	None
1	Mild: Transient or mild discomfort (< 48 hours); no medical intervention/therapy required
2	Moderate: Mild to moderate limitation in activity - some assistance may be needed; no or minimal medical intervention/therapy required
3	Severe: Marked limitation in activity, some assistance usually required; medical intervention/therapy required, hospitalization possible

Table 8. Severity grading of abnormal vital signs.

Parameter	Grade 1 (mild)	Grade 2 (moderate)	Grade 3 (severe)
Fever	37.6°C - 38.0°C	38.1°C – 39.0°C	>39.0°C
Tachycardia (bpm)*	101 – 115	116 – 130	>130
Bradycardia (bpm)**	50 – 54	40 – 49	<40
Systolic hypertension (mmHg)	141 – 159	160 – 179	≥180
Diastolic hypertension (mmHg)	91 – 99	100 – 109	≥110
Systolic hypotension (mmHg)***	85 – 89	80 – 84	<80

*Taken after ≥10 minutes at rest

**When resting heart rate is between 60 – 100 beats per minute. Use clinical judgement when characterising bradycardia among some healthy subject populations, for example, conditioned athletes.

***Use clinical judgement, particularly in subjects with low body weight. Consider checking lying and standing blood pressure to test for a postural drop of >20 mmHg if safe to do so. Do not record as an adverse event if hypotension is asymptomatic (e.g., absence of light-headed feeling)

stabilisation, or until causality is determined to be unrelated to trial interventions. All AEs will be managed as per Kenyan national clinical guidelines.

Moreover, any serious adverse event likely to be related to unithiol and occurring after trial termination should be reported by the investigator according to the procedure described below.

Outcome of any non-serious adverse event occurring within 6 months of the last dose of unithiol (i.e., unsolicited adverse event) or any SAE reported during the entire study will be assessed as:

- Recovered/resolved
- Not recovered/not resolved
- Recovering/resolving
- Recovered with sequelae/resolved with sequelae
- Fatal (SAEs only)

Subjects who have moderate or severe on-going adverse events that are not unithiol linked will be referred to an appropriate government hospital/health facility on completion of the study and will be advised to consult a physician if the event is not considered to be related to the study drug. If it is related to the study drug, a follow-up visit will be arranged to manage the problem and to determine the severity and duration of the event. If appropriate, specialist review using government services will be arranged.

Documenting AEs

Solicited and unsolicited AEs will be recorded on the participant's CRF. The diagnosis, date and time of onset, outcome, severity, and relationship to dosing will be established. Details of any treatment or concomitant interventions will be recorded.

These will be recorded from the time of written informed consent until 6 months post cessation of trial treatment.

The following information will be collected:

- Full details in medical terms and case description
- Event duration (start and end dates, if applicable)
- Action taken
- Outcome
- Seriousness criteria
- Causality (i.e., relatedness to trial drug / investigation), in the opinion of the investigator
- Whether the event would be considered expected or unexpected.

Reporting serious adverse events (SAEs) and/or unexpected AEs

- Every SAE occurring throughout the trial must be reported by telephone, e-mail, or fax to the sponsor (LSTM) and the DSMB within 48 hours, even if the investigator considers the SAE not related to the study drug. The investigator will then complete a hard copy SAE report as soon as possible and submit this to the sponsor, DSMB, SERU and PPB in accordance with their respective guidelines.
- Any relevant information concerning the adverse event that becomes available after the SAE report form has been sent (outcome, precise description of medical history, results of the investigation, copy of hospitalisation report, etc.) will be forwarded to the sponsor, DSMB, SERU and PPB in a timely manner, the anonymity of the subjects shall be respected when forwarding this information.
- The DSMB may ask for the study to be stopped, or for an extended study hold to be applied while further data and information are sought. The DSMB will make its recommendation to the Sponsor, who will have ultimate responsibility for acting on the recommendation.
- Any study related SUSAR or serious adverse event related to participation in the study must be reported by telephone, e-mail, or fax to the sponsor (LSTM) and the DSMB within 48 hours. A hard copy SUSAR report will be completed as soon as possible and submitted to the sponsor, DSMB, SERU and PPB in accordance with their respective guidelines.
- The SUSAR and SAE reports will be submitted to PPB through the online system at www.pv.pharmacyboardkenya.org. The sponsor pledges to inform the authorities of any trial discontinuation and specify the reason for discontinuation.

Emergency procedures

Equipment and drugs

The inpatient facility will be stocked with the appropriate equipment and drugs to manage emergencies that may arise whilst testing an IMP. This will include giving sets, intravenous fluids, oxygen, ventilation equipment and adrenaline.

Follow-up of participants

Every reasonable effort will be made to follow-up subjects who have adverse events. Any subject who has an ongoing adverse event at the follow-up visit will be followed up, if possible, until resolution.

Notification of deaths

All deaths will be reported to the sponsor, SERU, and PPB, irrespective of whether the death is related to disease progression, the IMP, or an unrelated event.

Pregnancy

All pregnancies within the study period (either the subject or the subject's partner) will be reported to the Principal Investigator and Sponsor within 24 hours of notification. The pregnancy will be followed to term at minimum, and the baby will be examined at 6 weeks after delivery (with further reviews to be arranged at the discretion of the responsible paediatrician).

Procedures for reporting any protocol violation(s)

If any changes to the study are necessary during the study a formal amendment will be presented to the sponsor prior to

submission to the relevant ethical and regulatory agencies for approval unless to eliminate an immediate hazard(s) to study participant without prior ethics approval. Any unforeseen and unavoidable deviations from the protocol will be documented and filed as a protocol non-compliance in the Trial Master File, with explanation.

Statistics

The statistical analysis plan has been uploaded to the Harvard Dataverse[41]. The study is not powered to detect statistically significant differences between groups, although a cut-off p-value ≤0.05 will be applied to any comparisons.

Pharmacokinetics

Pharmacokinetic outputs will include drug concentrations at each dose level during the first 24-hours and at each follow-up visit. Except for preliminary data to guide multiple dosing regimens that avoid accumulation, pharmacokinetic analysis will be completed after all participants have been dosed. Pharmacokinetic analysis will be undertaken at the University of Liverpool, UK. Area under the curve will be calculated from the plasma drug concentration time curve during the initial 24-hours. The maximal concentration (Cmax), time to maximum concentration (Tmax) and the half-life (t1/2) will be calculated.

Pharmacodynamics

Pharmacodynamic outputs will vary between assay and are exploratory. For the fluorogenic substrate assay the area under the fluorescence-time curve will be compared between dosing groups using ANOVA and an appropriate post-hoc test (e.g., Tukey's). The time to plateau and time to mid-point of fluorescence (Km) of the fluorescence-time curves will also be compared with this approach. These same measures will be calculated for the plasma clotting assay. Further assays will be developed, and analysis will be planned as appropriate.

Demographics and baseline characteristics

Demographic data will include age, gender, and race. Baseline data will include medical history, drug history, height, weight, full blood count, renal function, liver function and electrocardiogram. These will be presented using tables and descriptive statistics. Continuous variables will be described using means, standard deviations, medians, and interquartile ranges, as appropriate. Categorical data will be presented as counts and percentages. Missing data will be presented.

Safety analyses

Adverse event frequency, severity grade (mild, moderate, or severe), and relationship to IMP (no relationship, unlikely, possible, probable, and definite) will be described and reported to the DSMB before any dose escalations. These will be presented by organ system involved and clinically appropriate terminology will be used. Changes to alanine aminotransferase (ALT), plasma creatinine, haemoglobin, white cell count and platelet count will be presented to the DSMB at every safety review. Grading of laboratory values will use the Division of AIDS (DAIDS) Table for Grading the Severity of Adult and Paediatric Adverse Events version 2.1 July 2017[42]. These scales are not based on a coastal Kenyan population. We have modified these for several of our SERU approved trials (R21 (SERU 3711), S4V01 (SERU 3796, CHMI-SIKA (SERU 3190)) to ensure grading reflects normative values in our population.

Amendments to the statistical analysis plan

Minor amendments to the statistical analysis will be made by the investigator/supervisor/statistician. Any changes that significantly affect the protocol or reporting of adverse events will require approval from the sponsor and research ethics committees and regulatory authority.

Data management

The data management plan has been uploaded to the Harvard Dataverse (https://doi.org/10.7910/DVN/LJOYSO). The PI will have overall responsibility for managing the data. A designee to the PI will be responsible for receiving, entering, cleaning, querying, analysing, and storing all data from the study. Responsibility for this may be delegated to the study data management team.

Data capture methods

Data will be entered into the participant's paper CRF and subsequently transferred to an electronic database for analysis. A clinical quality management plan (CQMP) will be developed prior to the trial commencing. This will outline the processes for evaluating appropriate use of consent forms, CRFs and any study specific procedures (SSPs).

A laboratory analytical plan (LAP) will be developed prior to the study commencing. Lab samples will be tracked, and clinical results will be reported using the electronic system.

Archiving

The investigator must keep the consent forms and trial master file for at least 10 years after the completion or discontinuation of the trial. The anonymized electronic databases will be maintained beyond this period.

Missing data

Missing data will be recorded as such using appropriate coding that is compatible with statistical software. Missing data that impacts on the validity of the results will be clearly reported in scientific publications of this work. Records will be kept detailing the reason for any missing data and occurrence of missing data will be kept to a minimum.

Patient data protection/confidentiality

All study data for analysis will be de-identified. Participants will be identified via their unique study number. Paper records containing study data will be stored in a locked filing cabinet in a lockable study office. Electronic data will be stored on laptops approved by KEMRI-CGMRC IT department and will be password protected with encrypted hard-drives. Data will be backed up on the KEMRI-CGMRC cloud storage system, which is secured with password protection and approved for use for clinical research data. Participant information will only be shared with those directly involved in conducting this trial.

The master list of participant identifiable information will not be removed from the KEMRI-CGMRC site where it will be stored in a locked cabinet in a lockable office.

Archiving and record retention
Hard copies of documents will be kept for a period of 10 years following completion of the trial. Data and clinical samples will be kept for 15 years at KWTRP. Samples sent to the UK will be kept for a maximum of 10 years and then either destroyed or returned to KWTRP.

Data sharing
- Data will be shared with LSTM Centre for Snakebite Research & Interventions (CSRI) and the University of Liverpool, and this will be stated on the consent form and patient information leaflet.
- Liverpool School of Tropical Medicine is the Sponsor
- Prior to transfer of data, participants' names and other identifiable information will be removed.
- Data will only be accessed by individuals with a direct role in conducting this research.
- Access by others would require appropriate approvals including from SERU.
- Data published in peer-reviewed journal and presented at scientific meetings will be available open access. Participants will not be identifiable from the data presented.

Study monitoring
Regular monitoring will be performed according to International Council for Harmonisation (ICH) Good Clinical Practice (GCP) and a Monitoring Plan. Monitors will check whether the clinical trial is conducted, and data are generated, documented, and reported in compliance with the protocol, GCP and the applicable regulatory requirements. The site team led by the PI will be responsible for local submissions to the regulators and all the staff will have good clinical practice training prior to study start. The PI will give direct access to study documents for monitoring and auditing.

Quality control and quality assurance
The study team at the trial site will conduct regular quality checks to ensure that the trial is being conducted, data is being recorded, analysed, and accurately reported according to the protocol, trial standard operating procedures (SOPs) and the clinical quality management plan, and in compliance with ICH GCP. Audits will be conducted in the laboratories according to an agreed audit schedule.

Ethics
Approval will be sought from the CGMRC Centre Scientific Committee (CSC, Kilifi KEMRI Scientific & Ethics Review Unit (SERU), LSTM research and ethic committee (REC) and the Pharmacy and Poisons Board. Written informed consent will be obtained from each participant.

Human Subjects
As this trial will involve human subjects, the following guidelines will be observed:

"First, do no harm."

The Investigators are aware that this phase I trial exposes risk to healthy participants. Unithiol is a medication which has been used in humans for several decades and has reassuring safety data. Higher doses than have previously been administered will be used. The animal data suggests low toxicity at higher dose levels, as does the limited data from small observational reports in humans. Additional precautions will be undertaken during administration of higher dose levels, including sentinel dosing. Close monitoring will be in place for the 24-hours after dosing, and after this time plasma levels of unithiol are anticipated to be negligible. As data exist describing the adverse event profile of unithiol in humans, participants will be made aware of the solicited adverse events and early presentation to the study will be encouraged and supported if they occur. Trial insurance through the Sponsor will be in place and will cover the costs of treating any adverse events at appropriate government health facilities. As well as the risks that have been described above and in section 4 (safety data), there will be certain inconveniences. Subjects will be expected to reside at the in-patient facility during dosing and will be asked to attend follow-up appointments. Remuneration will be provided in accordance with KEMRI reimbursement guidelines. Volunteers will be made aware, during consenting, of their responsibilities and the inconveniences involved in becoming a study participant.

Direct benefit to study subjects or the community
There are limited direct benefits to those involved. The screening process may identify health conditions that would otherwise have gone unnoticed (such as high blood pressure or diabetes), and thus allow for earlier treatment. There may be some perceived benefits although this will just be close clinical monitoring during the trial.

There may be benefit to the local community if this compound were found to be effective in treating snakebite, although it should be noted that the research so far has suggested the strongest benefit would be seen for victims of a snake that reside in West Africa. West Africa is home to a type of snake (*Echis ocellatus* or saw-scaled viper) that causes many bites that result in uncontrolled bleeding. The experiments of unithiol in mice showed that the drug worked best against the venom of this snake. Further experiments of this drug for snakes that exist in Kenya are being undertaken. The target of this drug (SVMPs) is predominant in most viper species, and it is feasible that this drug may have efficacy for species relevant to Kenya, such as the puff adder.

Conducting the trial in Kilifi will provide a platform for further trials of new treatments for snakebite to be tested.

Community considerations
KEMRI-Wellcome has a dedicated team which coordinates all engagements with the programme. A CAST (Communication Advice for Studies) group will be formed and will develop a community engagement plan. The cast group will be composed of community liaison group (CLG) and study team members.

Kilifi North and South sub-County Hospital Management Teams (SCHMT) and Pwani University will be approached and informed of the study protocol.

Members of the Kilifi County living in Ngerenya, Chasimba and Kilifi township will be approached through community engagement activities. KWTRP personnel will raise awareness of the trial with permission of community leaders. There will be sensitization through the health management teams and village elders. Following this barazas will be held. At community meetings, focused pertinent information will be provided including the purpose, risks, duration, monetary compensation, exclusion criteria and inclusion criteria of the trial. Interested individuals will have ample time to read the PIS prior to consent. Those interested in the trial will be given written patient information in the appropriate language.

Informed consent
Potential participants will be provided with verbal and written information during community meetings. They will be encouraged to ask questions about the study. Potential participants that attend the screening visit will be given further information about the clinical trial, including the risks and their responsibilities. Potential participants will be given time to consider their decision and the opportunity to ask any questions. They will be aware that enrolment is voluntary and that they can withdraw. Participants that choose to withdraw at screening will be compensated for out-of-pocket expenses and their transport reimbursed according to the actual cost based on public transport. Consent forms will be available in English, Giriama and Swahili. Consent will be provided in writing and participants will maintain the right to withdraw at any time. Participants will be made aware that once they have taken the IMP that they would be advised to remain under close observation for their own safety. For participants that withdraw from the study after taking the IMP, all reasonable efforts will be made to maintain their safety, including telephone follow-up.

Compensation
As participants will be resident for at least 24-hours and have at least three further visits as out-patients, out of pocket expenses and travel costs will be incurred. These financial costs will be remunerated by the trial, according to KEMRI reimbursement guidelines. For each day engaged in the study, the participant will receive 500 KES [£3.50] (except for days where they will be accommodated for an overnight stay at the inpatient facility). Reserve participants that are not asked to attend for dosing will be reimbursed 500 KES [£3.50], as it is expected that they have made themselves available for the day of dosing. Per day and night spent as a resident, the participant will receive 2000 KES [£14]. These costs adhere to KEMRI guidelines and will be updated if they change in the period before trial is conducted. Costs of travel will vary depending on distance travelled and will be provided based on actual cost of using public transport.

Safety
All monitoring, clinical examination and sample collection will be undertaken by appropriately trained nursing and clinical staff. Subjects will be dosed and monitored in a clinical environment within an inpatient facility. A physician will be available throughout the study period. Costs of managing acute illness caused by the study drug will be met by the study and appropriate insurance will be in place for participants. Participants will be followed up for a minimum of 6 months. If there are ongoing features of adverse events at 6 months, follow-up will be extended where appropriate.

Reporting dissemination and notification of results
Results will be published in an open-access journal. Individual results with clinical relevance will be provided to participants in real-time. Summaries of the outcomes of the trial will be provided during community meetings in the areas from which participants are recruited. It is not anticipated that substantial information in this form will be available until at least the second year of the trial, and this will be made clear during initial meetings. Where possible, participants will receive feedback on the progress of the trial during their follow-up visits, including at the planned telephone consultation 6 months after dosing.

Study status
The study has started. Participant screening is ongoing.

Conclusions
Snakebite envenoming is an important cause of morbidity and mortality, contributing to more deaths per year than any other neglected tropical disease. There is an urgent need to identify new therapeutics for snakebite envenoming. Current antivenoms are prohibitively expensive, complex to manufacture, can only be administered intravenously, have a high risk of acute allergic reactions, and are restricted by high species specificity. Unithiol has the potential to overcome each of these limitations. SVMPs are an important component of many snake venoms, including: *Echis*, *Bitis*, *Daboia*, *Bothrops*, and *Crotalus*. Unithiol and other small molecule venom inhibitors therefore have the potential to inhibit a globally diverse range of snake venoms[43].

Before unithiol can be assessed in a phase II efficacy trial, further information is needed on an appropriate oral dose. Unithiol was developed as a treatment for heavy metal poisoning, for which treatment often consists of a short course of in-patient intravenous therapy, followed by a prolonged course of low dose oral therapy. The safety and tolerability of oral doses above 300 mg has not been confirmed, although reports from the use of high intravenous doses suggest it may be safe. For the treatment of snakebite, administration of therapeutic oral doses

is important, as many patients with snakebite will make initial contact with a healthcare facility at the local clinic level. There can often be a long delay before patients reach a secondary or tertiary care facility that stocks antivenom, and a potential advantage of unithiol is that it can be stocked and administered at the local clinic level.

This phase I clinical trial will assess the safety and tolerability of escalating oral doses of unithiol. A multiple dose regimen of oral unithiol, that offers consistent and safe plasma concentrations, will be defined. To better understand the pharmacokinetics of unithiol, two dosing groups will receive intravenous unithiol. It is anticipated that this trial will provide important information for developing a dosing regimen that can be progressed to a phase II clinical trial in patients with snakebite. This will be the first phase I clinical trial of an oral therapeutic for snakebite envenoming and represents an important step forward in improving therapeutic options for people with this neglected and dangerous disease.

Sponsor
The Liverpool School of Tropical Medicine in the Sponsor for this study. For further information, please contact the LSTM research governance department:

lstmgov@lstmed.ac.uk

+44(0)151 705 3100

Liverpool School of Tropical Medicine

Pembroke Place Liverpool

L3 5QA UK

Data availability
Underlying data
No underlying data are associated with this article.

Extended data
Harvard Dataverse KWTRP Research Data Repository: TRUE-1: Trial of Repurposed Unithiol for snakebite Envenoming Phase 1 (Safety, Tolerability, Pharmacokinetics and Pharmacodynamics in Healthy Kenyan Adults). https://doi.org/10.7910/DVN/LJOYSO[41].

This package contains the following extended data:
- Data Management Plan TRUE1_15102021.pdf
- DSMB charter TRUE1_14042021.pdf
- Statistical analysis plan TRUE1_31032021.pdf
- TRUE-1 PIL ICF v3_01012021.pdf

Data are available under the terms of the Creative Commons Attribution 4.0 International license (CC-BY 4.0).

References

1. Kasturiratne A, Wickremasinghe AR, de Silva N, *et al.*: **The Global Burden of Snakebite: A Literature Analysis and Modelling Based on Regional Estimates of Envenoming and Deaths.** *PLoS Med.* 2008; **5**(11): e218.
PubMed Abstract | Publisher Full Text | Free Full Text

2. Chippaux JP: **Snake-bites: appraisal of the global situation.** *Bull World Health Organ.* 1998; **76**(5): 515–524.
PubMed Abstract | Free Full Text

3. **Snake bite—the neglected tropical disease.** *Lancet.* 2015; **386**(9999): 1110.
PubMed Abstract | Publisher Full Text

4. Reid HA, Thean PC, Martin WJ: **Specific antivenene and prednisone in viper-bite poisoning: Controlled trial.** *Br Med J.* 1963; **2**(5369): 1378–1380.
PubMed Abstract | Publisher Full Text | Free Full Text

5. Habib AG, Brown NI: **The snakebite problem and antivenom crisis from a health-economic perspective.** *Toxicon.* 2018; **150**: 115–123.
PubMed Abstract | Publisher Full Text

6. Abubakar IS, Abubakar SB, Habib AG, *et al.*: **Randomised controlled double-blind non-inferiority trial of two antivenoms for saw-scaled or carpet viper (Echis ocellatus) envenoming in Nigeria.** *PLoS Negl Trop Dis.* 2010; **4**: e767.
PubMed Abstract | Publisher Full Text | Free Full Text

7. Habib AG, Onayade AA, Yakubu A, *et al.*: **First Clinical Experiences with a New Ovine Fab Echis ocellatus Snake Bite Antivenom in Nigeria: Randomized Comparative Trial with Institute Pasteur Serum (Ipser) Africa Antivenom.** *Am J Trop Med Hyg.* 1997; **56**(3): 291–300.
PubMed Abstract | Publisher Full Text

8. Warrell D, Davidson N, Omerod L, *et al.*: **Bites by the saw-scaled or carpet viper (Echis carinatus): trial of two specific antivenoms.** *Br Med J.* 1974; **4**(5942): 437–440.
PubMed Abstract | Publisher Full Text | Free Full Text

9. Warrell DA, Warrell MJ, Edgar W, *et al.*: **Comparison of Pasteur and Behringwerke antivenoms in envenoming by the carpet viper (Echis carinatus).** *Br Med J.* 1980; **280**(6214): 607–609.
PubMed Abstract | Publisher Full Text | Free Full Text

10. Abouyannis M, Aggarwal D, Lalloo DG, *et al.*: **Clinical outcomes and outcome measurement tools reported in randomised controlled trials of treatment for snakebite envenoming: A systematic review.** *PLoS Negl Trop Dis.* 2021; **15**(8): e0009589.
PubMed Abstract | Publisher Full Text | Free Full Text

11. Habib AG, Kuznik A, Hamza M, *et al.*: **Snakebite is Under Appreciated: Appraisal of Burden from West Africa.** *PLoS Negl Trop Dis.* 2015; **9**(9): e0004088.
PubMed Abstract | Publisher Full Text | Free Full Text

12. Rojnuckarin P, Chanthawibun W, Noiphrom J, *et al.*: **A randomized, double-blind, placebo-controlled trial of antivenom for local effects of green pit viper bites.** *Trans R Soc Trop Med Hyg.* 2006; **100**(9): 879–884.
PubMed Abstract | Publisher Full Text

13. Sellahewa KH, Gunawardena G, Kumararatne MP: **Efficacy of Antivenom in the Treatment of Severe Local Envenomation by the Hump-Nosed Viper (Hypnale hypnale).** *Am J Trop Med Hyg.* 1995; **53**(3): 260–262.
PubMed Abstract | Publisher Full Text

14. Asita de Silva H, Pathmeswaran A, Ranasinha CD, *et al.*: **Low-Dose Adrenaline, Promethazine, and Hydrocortisone in the Prevention of Acute Adverse Reactions to Antivenom following Snakebite: A Randomised, Double-Blind, Placebo-Controlled Trial.** *PLoS Med.* 2011; **8**(5): e1000435.
PubMed Abstract | Publisher Full Text | Free Full Text

15. Deutsch HF, Diniz CR: **Some proteolytic activities of snake venoms.** *J Biol Chem.* 1955; **216**(1): 17–26.
PubMed Abstract

16. Philpot VB, Deutsch HF: **Inhibition and activation of venom proteases.**

Biochim Biophys Acta. 1956; **21**(3): 524–530.
PubMed Abstract | Publisher Full Text

17. Goucher CR, Flowers HH: **The chemical modification of necrogenic and proteolytic activities of venom and the use of EDTA to produce Agkistrodon piscivorus, a venom toxoid.** *Toxicon.* 1964; **2**: 139–147.
PubMed Abstract | Publisher Full Text

18. Bjarnason JB, Tu AT: **Hemorrhagic toxins from Western diamondback rattlesnake (Crotalus atrox) venom: isolation and characterization of five toxins and the role of zinc in hemorrhagic toxin e.** *Biochemistry.* 1978; **17**(16): 3395–3404.
PubMed Abstract | Publisher Full Text

19. Rucavado A, Escalante T, Franceschi A, *et al.*: **Inhibition of local hemorrhage and dermonecrosis induced by Bothrops asper snake venom: effectiveness of early *in situ* administration of the peptidomimetic metalloproteinase inhibitor batimastat and the chelating agent $CaNa_2EDTA$.** *Am J Trop Med Hyg.* 2000; **63**(5–6): 313–319.
PubMed Abstract | Publisher Full Text

20. Ainsworth S, Slagboom J, Alomran N, *et al.*: **The paraspecific neutralisation of snake venom induced coagulopathy by antivenoms.** *Commun Biol.* 2018; **1**: 34.
PubMed Abstract | Publisher Full Text | Free Full Text

21. Albulescu LO, Hale M, Ainsworth S, *et al.*: **Preclinical validation of a repurposed metal chelator as a community-based therapeutic for hemotoxic snakebite.** *bioRxiv.* 2019; 717280.
Publisher Full Text

22. Chisolm JJ Jr: **BAL, EDTA, DMSA and DMPS in the Treatment of Lead Poisoning in Children.** *J Toxicol Clin Toxicol.* 1992; **30**(4): 493–504.
PubMed Abstract | Publisher Full Text

23. Van der Linde AA, Pillen S, Gerrits GP, *et al.*: **Stevens-Johnson syndrome in a child with chronic mercury exposure and 2,3-dimercaptopropane-1-sulfonate (DMPS) therapy.** *Clin Toxicol (Phila).* 2008; **46**(5): 479–481.
PubMed Abstract | Publisher Full Text

24. Sanotskiĭ VA, Zotova MG, Efimov VI, *et al.*: **[On the possibility of intravenous use of unithiol in high doses].** *Farmakol Toksikol.* 1967; **30**(4): 480–482.
PubMed Abstract

25. Aposhian HV: **DMSA and DMPS--water soluble antidotes for heavy metal poisoning.** *Annu Rev Pharmacol Toxicol.* 1983; **23**: 193–215.
PubMed Abstract | Publisher Full Text

26. Heyl: **Dimaval Solution Summary of Product Characteristics**. 2003; [cited 21 Feb 2020].
Reference Source

27. Hurlbut KM, Maiorino RM, Mayersohn M, *et al.*: **Determination and metabolism of dithiol chelating agents. XVI: Pharmacokinetics of 2,3-dimercapto-1-propanesulfonate after intravenous administration to human volunteers.** *J Pharmacol Exp Ther.* 1994; **268**(2): 662–668.
PubMed Abstract

28. Planas-Bohne F, Gabard B, Schäffer EH: **Toxicological studies on sodium 2,3-dimercaptopropane-1-sulfonate in the rat.** *Arzneimittelforschung.* 1980; **30**(8): 1291–1294.
PubMed Abstract

29. Szinicz L, Wiedemann P, Häring H, *et al.*: **Effects of repeated treatment with sodium 2,3-dimercaptopropane-1-sulfonate in beagle dogs.** *Arzneimittelforschung.* 1983; **33**(6): 818–821.
PubMed Abstract

30. **Dimaval®: Scientific Product Monograph.** Chromium | Selenium. In: Scribd. [cited 8 Jul 2019].
Reference Source

31. Stantschew S: **[Determination and elimination of mercury depots in persons exposed to mercury].** *Z Gesamte Hyg.* 1983; **29**(7): 388–390.
PubMed Abstract

32. Heinrich-Ramm R, Schaller H, Horn J, *et al.*: **Arsenic species excretion after dimercaptopropanesulfonic acid (DMPS) treatment of an acute arsenic trioxide poisoning.** *Arch Toxicol.* 2003; **77**(2): 63–68.
PubMed Abstract | Publisher Full Text

33. Maiorino RM, Dart RC, Carter DE, *et al.*: **Determination and Metabolism of Dithiol Chelating Agents. XII. Metabolism and Pharmacokinetics of Sodium 2,3-Dimercaptopropane-1-Sulfonate in Humans.** *J Pharmacol Exp Ther.* 1991; **259**(2): 808–14.
PubMed Abstract

34. Maiorino RM, Xu ZF, Aposhian HV: **Determination and metabolism of dithiol chelating agents. XVII. In humans, sodium 2,3-dimercapto-1-propanesulfonate is bound to plasma albumin via mixed disulfide formation and is found in the urine as cyclic polymeric disulfides.** *J Pharmacol Exp Ther.* 1996; **277**(1): 375–384.
PubMed Abstract

35. Maiorino RM, Gonzalez-Ramirez D, Zuniga-Charles M, *et al.*: **Sodium 2,3-dimercaptopropane-1-sulfonate challenge test for mercury in humans. III. Urinary mercury after exposure to mercurous chloride.** *J Pharmacol Exp Ther.* 1996; **277**(2): 938–944.
PubMed Abstract

36. Scott JA, Bauni E, Moisi JC, *et al.*: **Profile: The Kilifi Health and Demographic Surveillance System (KHDSS).** *Int J Epidemiol.* 2012; **41**(3): 650–657.
PubMed Abstract | Publisher Full Text | Free Full Text

37. Heyl: **Dimaval Oral Capsules Summary of Product Characteristics**. 2008; [cited 8 Jul 2019].

38. Sällsten G, Barregård L, Schütz A: **Clearance half life of mercury in urine after the cessation of long term occupational exposure: influence of a chelating agent (DMPS) on excretion of mercury in urine.** *Occup Environ Med.* 1994; **51**(5): 337–342.
PubMed Abstract | Publisher Full Text | Free Full Text

39. Ovaska H, Wood DM, House I, *et al.*: **Severe iatrogenic bismuth poisoning with bismuth iodoform paraffin paste treated with DMPS chelation.** *Clin Toxicol (Phila).* 2008; **46**(9): 855–857.
PubMed Abstract | Publisher Full Text

40. **The Association of the British Pharmaceutical Industry.** Guidelines for Phase I clinical trials 2018 edition. [cited 20 Nov 2019].
Reference Source

41. Abouyannis M: **TRUE-1: Trial of Repurposed Unithiol for snakebite Envenoming Phase 1 (Safety, Tolerability, Pharmacokinetics and Pharmacodynamics in Healthy Kenyan Adults).** Harvard Dataverse, V2, 2022.
http://www.doi.org/10.7910/DVN/LJOYSO

42. Division of AIDS: **Division of AIDS (DAIDS) Table for Grading the Severity of Adult and Pediatric Adverse Events**. 2017; [cited 31 Jan 2022].
Reference Source

43. Albulescu LO, Xie C, Ainsworth S, *et al.*: **A therapeutic combination of two small molecule toxin inhibitors provides broad preclinical efficacy against viper snakebite.** *Nat Commun.* 2020; **11**(1): 6094.
PubMed Abstract | Publisher Full Text | Free Full Text

Open Peer Review

Current Peer Review Status: ✓ ✓ ✓

Version 1

Reviewer Report 31 March 2022

https://doi.org/10.21956/wellcomeopenres.19564.r49209

© **2022 Coldiron M.** This is an open access peer review report distributed under the terms of the Creative Commons Attribution License, which permits unrestricted use, distribution, and reproduction in any medium, provided the original work is properly cited.

✓ **Matthew Coldiron**
Epicentre - Médicins Sans Frontières, Paris, France

The protocol of this trial is well-written and well-conceived, and I have no concerns about its design. I have no questions about the relevance of the study question, and I wish the investigators luck in carrying out this important trial. I only have a few minor points that the investigators might wish to consider, either now, or as they eventually report the results of their study.

- In the introduction, when describing the limitations of antivenom – and this is a minor point – it is true that allergic and other reactions vary by antivenom used (and even between production batches of the same commercial product), and in many cases this is likely due to intrinsic properties of the antivenom product. But some of the observed differences are also likely explained by a lack of standardization of classification/reporting in the few, mostly observational, studies that have been published.

- Randomisation – I am confused about the four randomly selected stand-by participants for each dosing cohort – do these come from the pool of screen-positives offered enrolment in the month prior to dosing, or is it an independent group?

- It might be worth considering a repeat EKG at the time of admission to the trial facility (Day -1). There are unlikely to be major changes from the screening done in the previous month, but given that all other clinical evaluations are being repeated (many of which are just as unlikely to change) at Day -1, why not do the EKG, too?

- On-site processing of clinical samples, as well as storage, are surely detailed in an LAP and study-specific SOPs, but it would be helpful to have a few basic elements described in the protocol – especially regarding the PK/PD samples – type of tube used for collection, target time from collection to intermediate/definitive processing, storage conditions, etc.

- For solicited AEs, even though potential participants with known asthma will be excluded, given the reports of asthma exacerbation with IV dosing of DMPS, perhaps it would be prudent to include wheeze or another asthma symptom on the list of solicited AEs in case participants with undiagnosed/unrecognized asthma are inadvertently included?

- Another small detail about safety is in the section on reporting pregnancies. There is a parenthetical remark "(either the subject or the subject's partner)" suggesting that there will be follow-up of children born to male participants during the study period. The protocol contains extensive discussion of the need for female participants to use effective contraception but makes no mention of the need for male participants to use contraceptive methods (or to ensure that their female partners are using contraception). This seems to be misaligned – either male participants should also be counseled to use contraceptive methods, or following up male participants' female partners' pregnancies could be removed, particularly as these women would not have given consent to participate in the study.

Is the rationale for, and objectives of, the study clearly described?
Yes

Is the study design appropriate for the research question?
Yes

Are sufficient details of the methods provided to allow replication by others?
Yes

Are the datasets clearly presented in a useable and accessible format?
Not applicable

Competing Interests: I participated (without compensation) in a Wellcome-funded process to develop a Core Outcome Set for clinical trials dealing with snake envenoming that was led by some of the investigators of this trial.

Reviewer Expertise: Design and conduct of clinical trials in sub-Saharan Africa

I confirm that I have read this submission and believe that I have an appropriate level of expertise to confirm that it is of an acceptable scientific standard.

Reviewer Report 28 March 2022

https://doi.org/10.21956/wellcomeopenres.19564.r49206

© 2022 Lewin M. This is an open access peer review report distributed under the terms of the Creative Commons Attribution License, which permits unrestricted use, distribution, and reproduction in any medium, provided the original work is properly cited.

Matthew Lewin
California Academy of Sciences, San Francisco, CA, USA

The paper is well written. I don't see any major issue to be addressed. It is a very important step towards the modernization of snakebite envenoming treatment. The investigators are taking a

disciplined, safety oriented, approach to evaluating a drug that has been around for some time. They are not taking any shortcuts.

Very minor comments/questions:

1. It is possibly worth mentioning the safety of metal chelation therapies in patients with sickle cell disease/trait patients. This has been studied. There are no major issues I am aware of (studies seem to indicate good safety) but seems worth noting.

2. CIV should be defined in the table subtext (I had to go back to the text to remind myself) and could consider a hyphen. CIV stands for a lot of things other than "cohort IV"...chemical injection valve, but more importantly "continuous IV infusion". Some people scan or read tables first to get a generic view of a paper before reading--or go back to the tables after reading but don't remember the abbreviations. Truly minor comment.

Two more observations - neither are major:

1. It is not immediately clear to me exactly how you will decide if it is 2 days vs. 3 days. Also, I am looking at the 3rd paragraph of the section titled, "Justification for the proposed dose, route of administration, dosage regimen and treatment period," and I don't see a clear justification for 2-3 days vs. a longer period of treatment. Maybe it's there, I just don't see it clearly explained.

2. The organization of Table 2 is helpful but there is a part I don't get. The last row is the single IV dose of 5-10 mg/kg. Not sure why this goes in the Multiple Dose section of the table. You do say (and CIV2) in the subheading but it was not easy for me as an outside reader to organize the groups. Why not just make it a separate bubble with a simple explanation (e.g. If x then y)?

Is the rationale for, and objectives of, the study clearly described?

Yes

Is the study design appropriate for the research question?

Yes

Are sufficient details of the methods provided to allow replication by others?

Yes

Are the datasets clearly presented in a useable and accessible format?

Yes

Competing Interests: My group is developing small molecule inhibitors of sPLA2 and metalloprotease inhibitors that might be construed as competing interest. I appreciated the trust and opportunity to review this in spite of that known potential competing interest. This is a very thoughtfully assembled and important study. If the drug ultimately works as hoped, it will be an important contribution to the treatment of patients suffering from snakebite envenoming.

Reviewer Expertise: Basic research, clinical medicine

I confirm that I have read this submission and believe that I have an appropriate level of expertise to confirm that it is of an acceptable scientific standard.

Reviewer Report 28 March 2022

https://doi.org/10.21956/wellcomeopenres.19564.r49208

© 2022 Lavonas E. This is an open access peer review report distributed under the terms of the Creative Commons Attribution License, which permits unrestricted use, distribution, and reproduction in any medium, provided the original work is properly cited.

Eric Lavonas
[1] Department of Emergency Medicine, Denver Health, Denver, CO, USA
[2] Rocky Mountain Poison and Drug Safety, Denver Health, Denver, CO, USA
[3] Department of Emergency Medicine, University of Colorado School of Medicine, Aurora, CO, USA

This is a thorough, clear, well-thought through clinical trial protocol. My suggestions are small and, I think, completely optional.

- Re: Timing of follow-up safety assessments: I think an additional assessment at 7 and/or 14 days would enhance safety. However, given the short half-life of unithiol, I see no need to have a follow up beyond the 42-day assessment. The 6-month assessment is likely to find a log of unrelated health events with little to no chance of finding clinically relevant safety information.

- Re: Exclusion criteria. I agree it is appropriate to exclude children, the elderly, and patients with asthma in this phase 1 trial. However, these patient populations do suffer snake envenoming. Can we comment on a plan to include them in future research, if unithiol proves safe in the current trial population?

- Re: Lab testing: I suggest also measuring serum/plasma concentrations of calcium, magnesium, and zinc.

- Re: Lab testing. I understand that unithiol is not metabolized by the CYP450 enzyme system. However, I don't think the current data exclude the possibility of a clinically important minor metabolite from one/more of these pathways. Suggest you obtain and hold blood for CYP450 and other relevant genotyping. That way, if a patient has a SAE, you have the samples you need to do genotyping in a case-control fashion and understand what happened.

- Re: Stability of lab samples: I do have some concern that unithiol / metabolites may be unstable in stored serum / plasma samples, even if frozen. Degradation during storage and transport to the UK and/or artifact from the freezing and storing process is a major threat to the study. I suggest you obtain multiple serum / plasma tubes at a few time points, run duplicate analyses, and measure the variance/reliability of your assay results. Of course, it would be better to run samples on-site, but I understand this might not be possible.

- Re: Multiple dosing: Please clarify dosing frequency in the multiple dosing arm. Are subjects receiving unithiol daily, or (as suggested on page 9, column 1, bottom) 4 times a day?
- Re: No placebo group. While I think this is ethical and perfectly OK for a phase 1 trial, please consider that there are known changes in liver function that occur when subjects are admitted to a clinical trials unit. These are likely related to diet and exercise (subjects eat more and exercise less when housed in a CTU). The changes can be hard to distinguish from early signs of liver injury. I apologize that I can't find the supporting reference for this, and it may be a non-issue in such a short protocol, but the trial would be cleaner if it included at least a few placebo subjects. Interestingly, there also appear to be systematic changes in liver test results placebo-group subjects in non-residential trials (see: https://journals.physiology.org/doi/full/10.1152/ajpgi.00051.2018).[1]
- Re: Vital signs: Axillary temperature is quite inaccurate. I suggest you use oral temperature. If there's a reason oral is not appropriate, then most studies show temporal artery temperature measurements are more accurate than axillary.
- Re: Follow up on infants born to exposed mothers: The plan as described is standard and probably adequate given the safeguards in place in this study, but it's worth noting that even major neurodevelopmental outcomes may not be apparent at 6 months of age. Additional assessments (even telephonic) at 12 and 24 months would improve safety surveillance for infants born to mothers in this study. Again, I think the risk is low, safety plans are thorough, and women who are or could become pregnant are a relevant population to snake envenoming, so I am not criticizing the plan.
- Re: PK calculations: Before calculating the elimination half-life, it is worth assessing the elimination for fit. With enough subjects and sampling, you may find mixed kinetics.
- Re: Elimination: Should the study also assess for the fate of metabolites?
- Re: Pharmacodynamic outputs: This is an area I know very little about, and can provide no expertise. Please ensure the protocol is reviewed by someone with a good working familiarity with the methods proposed here.

Thank you for inviting me to review this excellent protocol. The work proposed is important and will be useful to benefit patients with snake envenoming.

References

1. Nunez D, Alexander M, Yerges-Armstrong L, Singh G, et al.: Factors influencing longitudinal changes of circulating liver enzyme concentrations in subjects randomized to placebo in four clinical trials. *American Journal of Physiology-Gastrointestinal and Liver Physiology*. 2019; **316** (3): G372-G386 Publisher Full Text

Is the rationale for, and objectives of, the study clearly described?

Yes

Is the study design appropriate for the research question?

Yes

Are sufficient details of the methods provided to allow replication by others?
Yes

Are the datasets clearly presented in a useable and accessible format?
Yes

Competing Interests: Dr. Lavonas served, without compensation, on a Wellcome Trust working group to develop a core outcome dataset for snake envenoming trials. Several of the current study authors were also members of this group.

Reviewer Expertise: Snake envenomation, clinical trials, resuscitation

I confirm that I have read this submission and believe that I have an appropriate level of expertise to confirm that it is of an acceptable scientific standard.
